# Exploration of acetylation-related biomarkers in osteoarthritis through bioinformatics analysis

**DOI:** 10.1371/journal.pone.0357231

**Published:** 2026-08-28

**Authors:** Shuchang Li, Jiefeng Yin, Jie Huang, Xifan Zheng, Jun Yao

**Affiliations:** 1 Bone and Joint Surgery, The First Affiliated Hospital of Guangxi Medical University, Nanning, Guangxi, China; 2 Department of Surgery II, Wuxiang Hospital, The Third Affiliated Hospital of Guangxi Medical University, Nanning, Guangxi, China; The University of Sheffield, UNITED KINGDOM OF GREAT BRITAIN AND NORTHERN IRELAND

## Abstract

Osteoarthritis (OA) is characterized as a chronic degenerative disorder affecting the joints. A growing body of evidence indicates that acetylation may play a role in the disease’s pathogenesis. However, the underlying molecular mechanisms remain largely undefined. The objective of this study was to explore potential biomarkers linked to acetylation in OA through a comprehensive bioinformatics analysis. We utilized datasets GSE55235, GSE55457, and GSE12021 from the Gene Expression Omnibus (GEO) to identify differentially expressed genes (DEGs) by employing the limma package, followed by functional enrichment analyses. By implementing weighted gene co‑expression network analysis (WGCNA), we identified key modules and subsequently recognized acetylation-related differentially expressed genes (ACEDEGs). A protein-protein interaction (PPI) network was constructed for these ACEDEGs, and machine learning algorithms were applied to discover potential biomarkers. We established and validated a diagnostic prediction model demonstrating significant diagnostic efficacy (AUC: 0.983 for training and 0.743 for validation). Furthermore, analyses indicated notable alterations in immune cell infiltration through CIBERSORT. Additionally, qRT-PCR and Western blotting corroborated the down-regulation of biomarkers JUN and MYC in OA. In summary, JUN and MYC were identified as novel acetylation-related biomarkers in OA. These findings offer valuable insights into the disease’s pathophysiology and suggest new pathways for diagnostic and therapeutic strategies.

## Introduction

Osteoarthritis (OA) is a prevalent degenerative joint disease characterized by the progressive degradation of articular cartilage, synovial inflammation, and alterations in subchondral bone structure [1]. The disease primarily affects older adults, contributing significantly to chronic pain, functional disability, and an increased burden on healthcare systems globally [2,3]. Despite the high prevalence and substantial economic impact of OA, no effective disease-modifying treatment is currently available, with existing therapeutic options primarily focused on symptom management, such as pain relief and physical rehabilitation [4,5]. This gap in effective management highlights the pressing need for innovative approaches to enhance OA diagnosis and treatment, particularly in identifying reliable biomarkers that can facilitate early intervention and disease monitoring [6].

Recent advancements in molecular biology and genomics have highlighted the critical role of various cellular processes in the pathogenesis of OA [7,8]. Among these, post-translational modifications, such as acetylation, have emerged as significant contributors to the regulation of gene expression and cellular function. Dysregulation of acetylation has been related to several pathological processes associated with OA, including inflammation and cartilage degradation [9]. However, the specific mechanisms by which acetylation influences OA remain poorly understood, indicating a knowledge gap that warrants further exploration [10,11]. Previous studies have suggested that the identification of acetylation-related biomarkers may yield valuable insights into OA pathology and potential therapeutic targets [12,13].

To address this knowledge gap, our study aimed to explore the role of acetylation-related genes in OA pathogenesis. Utilizing advanced computational techniques, we aimed identify key acetylation-related differentially expressed genes (DEGs) that may serve as robust biomarkers for OA. This approach not only enhances our understanding of the molecular foundations of OA but also enables the identification of potential therapeutic targets for clinical application [14,15]. The integration of bioinformatics tools, such as weighted gene co-expression network analysis (WGCNA) and protein–protein interaction (PPI) networks, combined with experimental validation methods like quantitative reverse transcription polymerase chain reaction (qRT-PCR) and Western blotting (WB), provides a comprehensive framework for our investigation [16,17].

Our primary objective is to identify acetylation-related biomarkers and evaluate their diagnostic utility and interactions within the immune microenvironment of OA. By elucidating the roles of these genes in OA progression and their potential as biomarkers, this research could facilitate the development of more effective diagnostic and therapeutic strategies [18]. Additionally, we aimed to construct a predictive nomogram based on our findings to facilitate risk stratification and early diagnosis of OA, thereby improving patient outcomes and informing clinical decision-making [19–21].

In summary, this study seeks to address the deficiencies in OA research by investigating the role of acetylation-related genes and their potential as biomarkers for disease diagnosis and treatment. Through a combination of computational and experimental methodologies, we aim to provide new insights into the molecular mechanisms of OA, ultimately contributing to the development of highly effective therapeutic interventions and improving the quality of life for affected patients [22]. The anticipated identification of reliable biomarkers may foster the establishment of targeted therapies that address the underlying pathophysiological processes of OA, thereby significantly advancing OA research [23,24].

## Materials and methods

### Data acquisition and preprocessing

The GSE55235, GSE55457, and GSE12021 datasets containing data on the synovial membrane were obtained from the Gene Expression Omnibus (GEO) database (https://www.ncbi.nlm.nih.gov/geo/). Probe names were converted to gene names using Perl and with the help of platform annotation files. Background correction and normalization of each dataset were performed using the R package limma, and the three synovial datasets from the same platform were integrated using the R package sva to remove batch effects [25]. For the subsequent analyses, the integrated dataset was utilized as the training cohort,which contained 29 OA groups and 29 control groups. The GSE82107 dataset was designated as the validation cohort, which included 10 OA groups and 7 control groups. The genes associated with Acetylation were obtained from the GeneCards database (https://www.genecards.org/, (GeneCards Version 5.25 Updated: Jul 16, 2025)). A total of 4,405 acetylation-related genes were obtained by applying a relevance score threshold of greater than 2 as a criterion for selection.

### Identification of DEGs and enrichment analysis

In this study, the R limma package was utilized to identify DEGs within the processed microarray data, using the following filtering criteria: |log_2_ Fold Change (FC)| > 0.5 and p value < 0.05. Genes that satisfied these conditions were classified as DEGs. Following this, Gene Ontology (GO) analysis and Kyoto Encyclopedia of Genes and Genomes (KEGG) analysis were conducted to further investigate the functional annotations and pathways correlated with the identified DEGs [26].

### Weighted gene co‑expression network analysis (WGCNA)

WGCNA was employed to construct modules associated with OA by utilizing gene expression profiles. Following the computation of the Spearman correlation coefficient, a similarity matrix was established [27]. A soft threshold of 14 was selected, which facilitated the conversion of the similarity matrix into an adjacency matrix, which was transformed into a topological overlap matrix (TOM). The TOM was then utilized for average-linkage hierarchical clustering, allowing for the classification of gene modules, each of which comprised a minimum of 70 genes. Upon merging analogous gene modules, five distinct modules were identified, with the red module recognized as the primary module of interest.

### Selection of ACEDEGs and construction of PPI network

The “VennDiagram” software package was used to illustrate the shared genes among the core module genes, acetylation-related genes, and DEGs. The genes within this intersection were designated as differentially expressed genes related to Acetylation-related genes (ACEDEGs). The PPI network of ACEDEGs was constructed through the STRING online platform (https://david.ncifcrf.gov/). We then set the network to have a minimum required interaction score greater than 0.4 based on the STRING online database,. Subsequently, The PPI network was imported into Cytoscape (version 3.9.1), and the top 13 genes were screened for subsequent analysis based on the MCC algorithm of the cytoHubba plugin.

### Screening for hub genes via machine learning algorithms

Three machine learning algorithms, namely, Least Absolute Shrinkage and Selection Operator (LASSO), Random Forests (RF), and eXtreme Gradient Boosting (XGBoost) were used to screen OA hub genes. The “glmnet” package was used for LASSO regression analysis, tenfold cross-validation was adopted, and the minimum lambda value was identified as the optimal solution. We performed RF using the randomForest package and XGBoost using the XGBoost package.

### Nomogram construction and receiver operating characteristic (ROC) evaluation

Univariate and multivariate regression analyses were conducted on three hub genes, and a predictive nomogram that incorporates the expression levels of JUN and MYC was developed utilizing the rms package (version 6.7−1). Furthermore, decision curve analysis (DCA) was employed to assess clinical utility over a range of threshold probabilities (0–1.0), revealing a net benefit close to 0.2 compared with alternative strategies. The “pROC” R package was utilized to visualize the area under the ROC curve (AUC) for evaluating the diagnostic model and identifying the two diagnostic genes. Finally, ROC curve analysis was conducted on the GSE82107 dataset to confirm the diagnostic efficacy of the proposed model.

### Evaluation and correlation analysis of infiltration‑related immune cells

The CIBERSORT algorithm (https://cibersortx.stanford.edu/) was employed to examine the patterns of immune cell infiltration within the OA and control groups [28]. This algorithm processed the tissue expression matrix utilizing linear support vector regression, thereby facilitating an accurate quantification of 22 distinct immune cell types present in each sample [29]. The analysis was conducted using the “CIBERSORT” package, where “perm” was set to 100 and the “QN argument” was specified as “TRUE.” Furthermore, the “ggplot2” package was utilized to effectively illustrate the correlation between the expression of diagnostic genes and immune cell infiltration. Enrichment scores for the control and OA samples across 28 immune cell types were computed utilizing the R package GSVA, and the findings were subsequently visualized. Additionally, Spearman correlation analysis was performed to assess the relationship between biomarkers and immune cell populations.

### Gene set enrichment analysis (GSEA) analysis

GSEA was performed using the enrichR package in R. We divided each biomarker into two groups based on expression levels: the top 50% was the high expression group, and the bottom 50% was the low expression group. Subsequently, we used the GSEA algorithm to calculate the enrichment scores and p-values for each gene set.

### Isolation and cultivation of rat articular chondrocytes and development of an IL-1β-induced OA cell model

The experiments in this article received approval from the Ethics Committee of the First Affiliated Hospital of Guangxi Medical University, which provided a written ethics certificate (Ethics Application (2025-E0960)). All experimental methods followed the relevant guidelines and regulations, as well as the ARRIVE guidelines. Two 7-day-old healthy Sprague–Dawley (SD) rats, weighing 10.2 and 11.8 g respectively, were purchased from the animal experimentation center of Guangxi Medical University. The rats received intraperitoneal injections of pentobarbital sodium at a doses of 40 mg/kg to induce anesthesia, which caused them to lose consciousness. Subsequently, the rats were then euthanized by cervical dislocation, and cartilage tissues were extracted, washed, minced, and digested for chondrocyte culture in Dulbecco’s Modified Eagle Medium (DMEM) supplemented with serum and antibiotics. To induce inflammation for the OA model, we treated with IL-1β, while a control group was maintained without stimulation.

### Quantitative real-time polymerase chain reaction (qRT-PCR)

The total RNA in the tissue was extracted using the kit method (R0032, Beyotime, China). Subsequently, The RNA was reverse-transcribed into cDNA using a PrimeScript^TM^ RT Master Mix (TakaRa, China). Thereafter, PCR experiments were conducted using the HotStart^TM^ Universal 2X SYBR Green qPCR Master Mix (K1170, APExBIO, USA). The mRNA expression level of the biomarkers was computed using the 2^−ΔΔCt^ method and normalized against GAPDH. The primer sequences utilized in the study were as follows:JUN (forward primer), 5′ -CAGCCGCCGCACCACTTG – 3′ and (reverse primer) 5′- TGATCCGCTCCTGAGACTCCATG – 3′; MYC (forward primer), 5′ - AGTGCTGCATGAGGAGACAC – 3′ and (reverse primer) 5′ - GCCT CTTC TCCA CAGA CACC- 3′.

### Protein extraction and WB analysis

We used WB to assess the protein expression levels of the biomarkers. Total protein was extracted from the tissues using RIPA buffer (strong) (K1020, APExBIO, USA) containing protease and phosphatase inhibitors, separated by SDS-PAGE, and electrotransferred onto polyvinylidene fluoride membranes. Following this, the membranes were incubated with an anti-JUN primary antibody (WB 1:1000, Zenbio, 380397, China) and an anti-MYC primary antibody (WB 1:1000, Zenbio, R380784, China), then with the corresponding secondary antibody (31460, Invitrogen, USA, 1:10,000). GAPDH Rabbit mAb (AF7018, Affnity, USA, 1:20,000) and α-tubulin Rabbit mAb (11224–1-AP, Proteintech, China, 1:5,000) served as the controls. Thereafter, the membranes were visualized using a chemiluminescence reagent (K1129, APExbio, USA) and analyzed quantitatively using GraphPad 9.5 software (GraphPad, USA).

### Statistical analysis

Bioinformatics analyses were performed using R software version 4.3.3, with significance set at p < 0.05. Comparisons between the two groups were evaluated using Student’s t-test. Additionally, correlations were assessed using Spearman’s coefficient. Visual data representations were created using R and GraphPad 9.5. Statistical significance was set at ^*^p < 0.05, ^**^p < 0.01, and ^***^p < 0.001.

## Results

### Identification of DEGs

**Fig 1:**
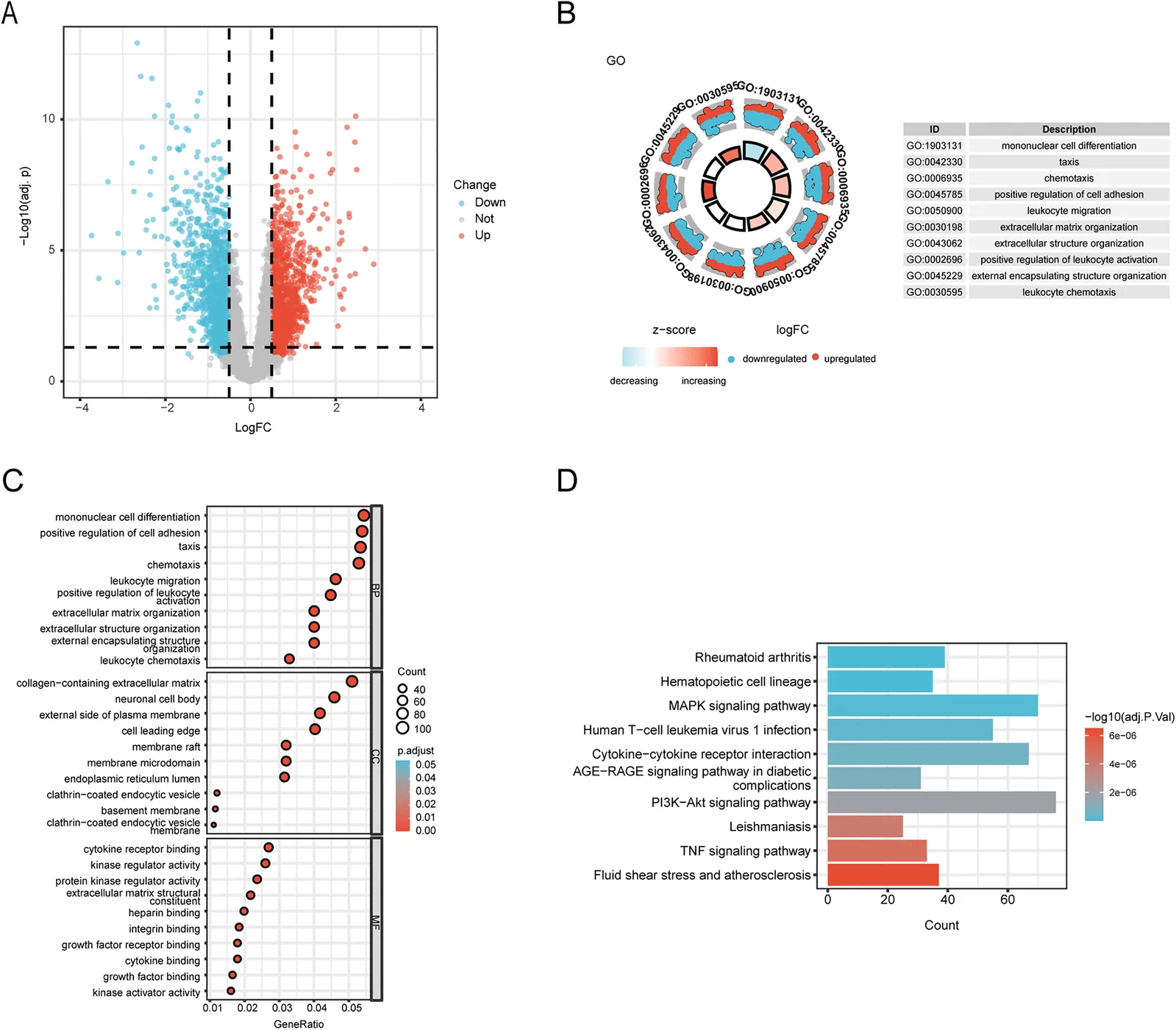
DEGs of OA were identified and functional enrichment analysis was performed. (**A**) The volcano plot showed 2,402 DEGs between the OA and control groups in the combined dataset. (**B**) and (**C**) GO analysis of DEGs. (**D**) KEGG pathway analysis of DEGs.

A comparative analysis between the OA and control groups revealed a total of 2,402 DEGs, comprising 1,185 that were upregulated and 1,217 that were downregulated. For detailed information, see S1 File. The volcano plot effectively showcased the significant DEGs (Fig 1A), and GO results were combined with the logFC circle diagram (Fig 1B). To enhance comprehension of the biological roles of the DEGs, we performed GO and KEGG enrichment analyses. GO analysis demonstrated that these DEGs were correlated with various biological processes including mononuclear cell differentiation, positive regulation of cell adhesion, leukocyte migration, and extracellular matrix organization. In terms of cell components, DEGs were primarily associated with collagen-containing extracellular matrix, neuronal cell body, external side of plasma membrane, membrane microdomains, and cell’s leading edge. The most enriched terms for molecular function included cytokine receptor binding, kinase regulator activity, protein kinase regulator activity, extracellular matrix structural constituent and growth factor receptor binding (Fig 1C). KEGG enrichment analysis revealed that the DEGs were primarily linked to the MAPK signaling pathway, PI3K-Akt signaling pathway, TNF signaling pathway, Human T-cell leukemia virus 1 infection, and cytokine-cytokine receptor interaction (Fig 1D). Detailed results of GO and KEGG enrichment can be found in S2 File.

### Construction of co-expression networks and module screening

**Fig 2:**
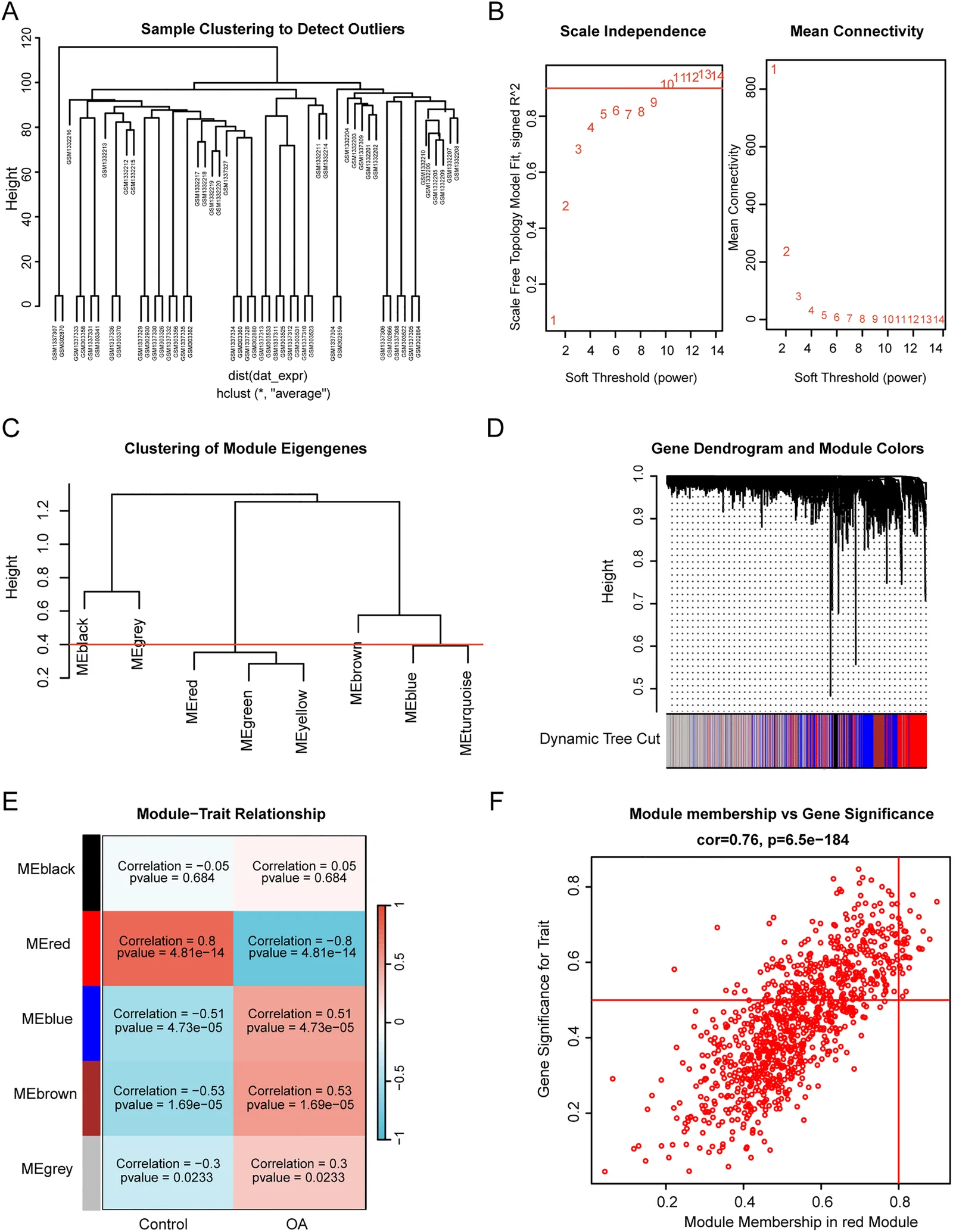
Construction of weighted co-expression network-related datasets in OA. (**A**) A cluster tree of 58 samples. (**B**) Analysis of network topology for various soft thresholds (β). (**C**) Clustering dendrogram of genes. (**D**) Gene dendrograms from average linkage hierarchical clustering. (**E**) Module-trait relationships. (**F**) A scatterplot of gene significance for OA vs module membership in the red module.

To define co-expressed genes, the “average correlation degree” alongside “Spearman correlation values” was computed to cluster the samples within the combined dataset, excluding outliers (Fig 2A). Subsequently, with a scale independence threshold of > 0.9, we determined 14 as the “soft threshold power β” to facilitate the establishment of biologically significant scale-free networks (Fig 2B). Hierarchical clustering analysis and dynamic TreeCut identified eight distinct modules (Fig 2C and 2D). The results from the module trait correlation analysis demonstrated a significant positive correlation between the red module and OA (cor = 0.8, pvalue = 4.81e-14), leading to its selection for further analysis (Fig 2E). Within the red module 973 genes were identified, with a scatter plot illustrating a robust correlation between these genes and OA (cor = 0.76, p = 6.5e − 184; Fig 2F). For detailed information, see S3 File.

### Identification of ACEDEGs and PPI network analysis

**Fig 3:**
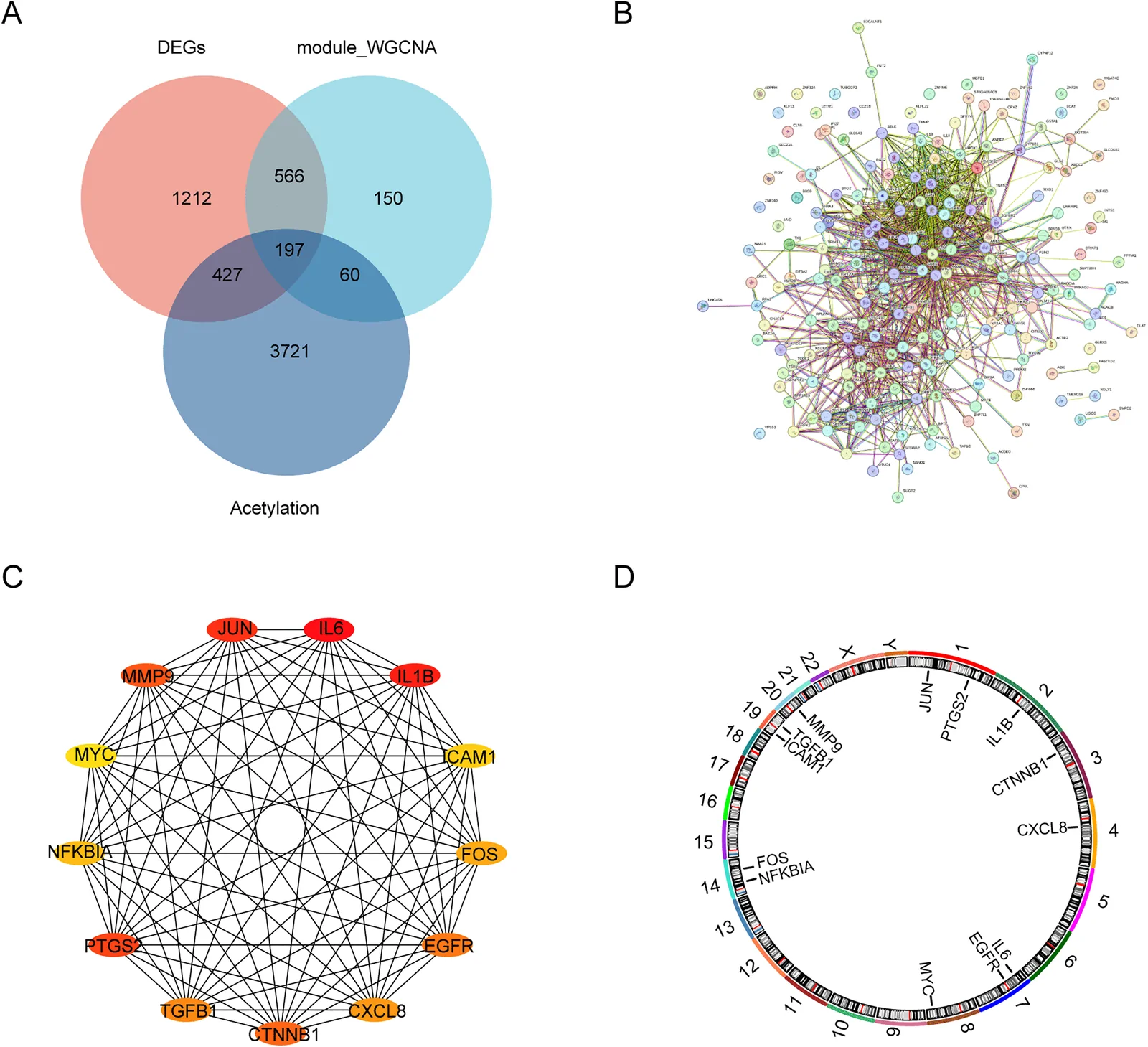
Selecting ACEDEGs and PPI network construction. (**A**) Venn plot of the 197 ACEDEGs. (**B**) PPI network analysis among 197 ACEDEGs. (**C**) Cytoscape network visualization of the 13 ACEDEGs. (**D**) Circular plot showing the chromosomal locations of the 13 ACEDEGs.

The intersection of red module genes, acetylation-related genes and DEGs yielded 197 ACEDEGs (Fig 3A). The obtained PPI network had 197 nodes, 1,068 edges, and an average node degree of 10.8 (Fig 3B). The top 13 genes with the highest scores were identified using the Cytoscape CytoHubba plugin (MCC algorithm) (Fig 3C). For detailed information, see S4 File. Subsequently, we examined the locations of 13 ACEDEGs on human chromosomes (Fig 3D). The results showed that JUN was located on chromosome 1 and MYC was on chromosome 8. For detailed information, see S5 File.

### Machine learning

**Fig 4:**
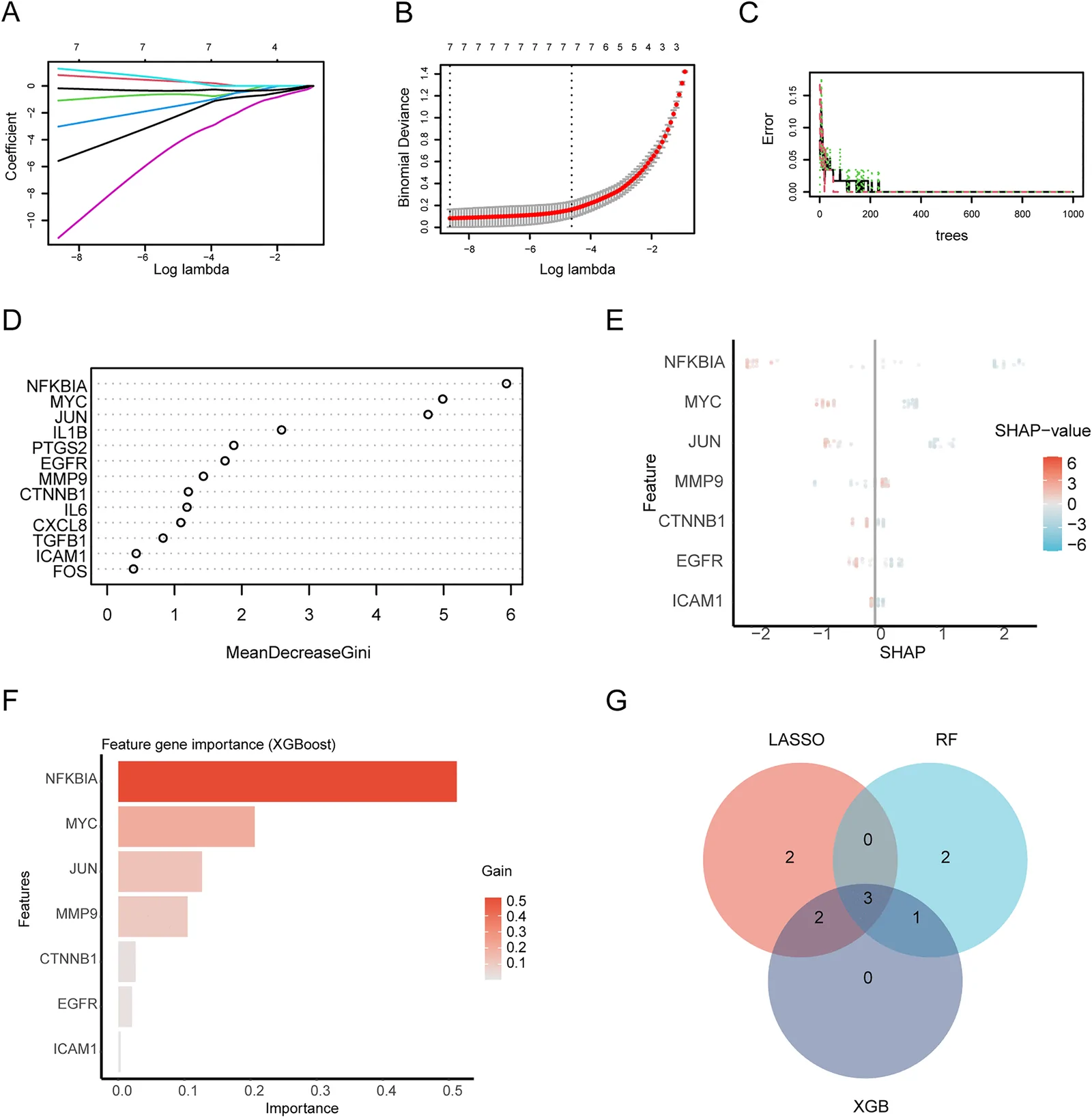
Detection of hub genes using machine-learning algorithms. (**A**, **B**) Based on LASSO regression algorithm to screen hub genes. (**C**, **D**) Based on RF algorithm to screen hub genes. (**E, F**) The implementation of the XGBoost algorithm for the identification of hub genes. (**G**) Venn diagram showcased three hub genes shared among LASSO, RF and XGBoost.

Three distinct machine-learning algorithms, namely LASSO, RF, and XGBoost were utilized to discover potential hub genes for OA. The LASSO regression algorithm was utilized to construct a model, resulting in the selection of seven genes (Fig 4A and 4B). Similarly, the RF algorithm was employed to construct a model, leading to the selection of six genes (Fig 4C and 4D). Furthermore, the XGBoost algorithm was employed to create a model that led to the selection of six genes (illustrated in Fig 4E and 4F). Finally, by intersecting the results derived from these three machine learning methodologies, three hub genes were identified (Fig 4G). For detailed information, see S6 File.

### Construction and validation of a predictive nomogram

**Fig 5:**
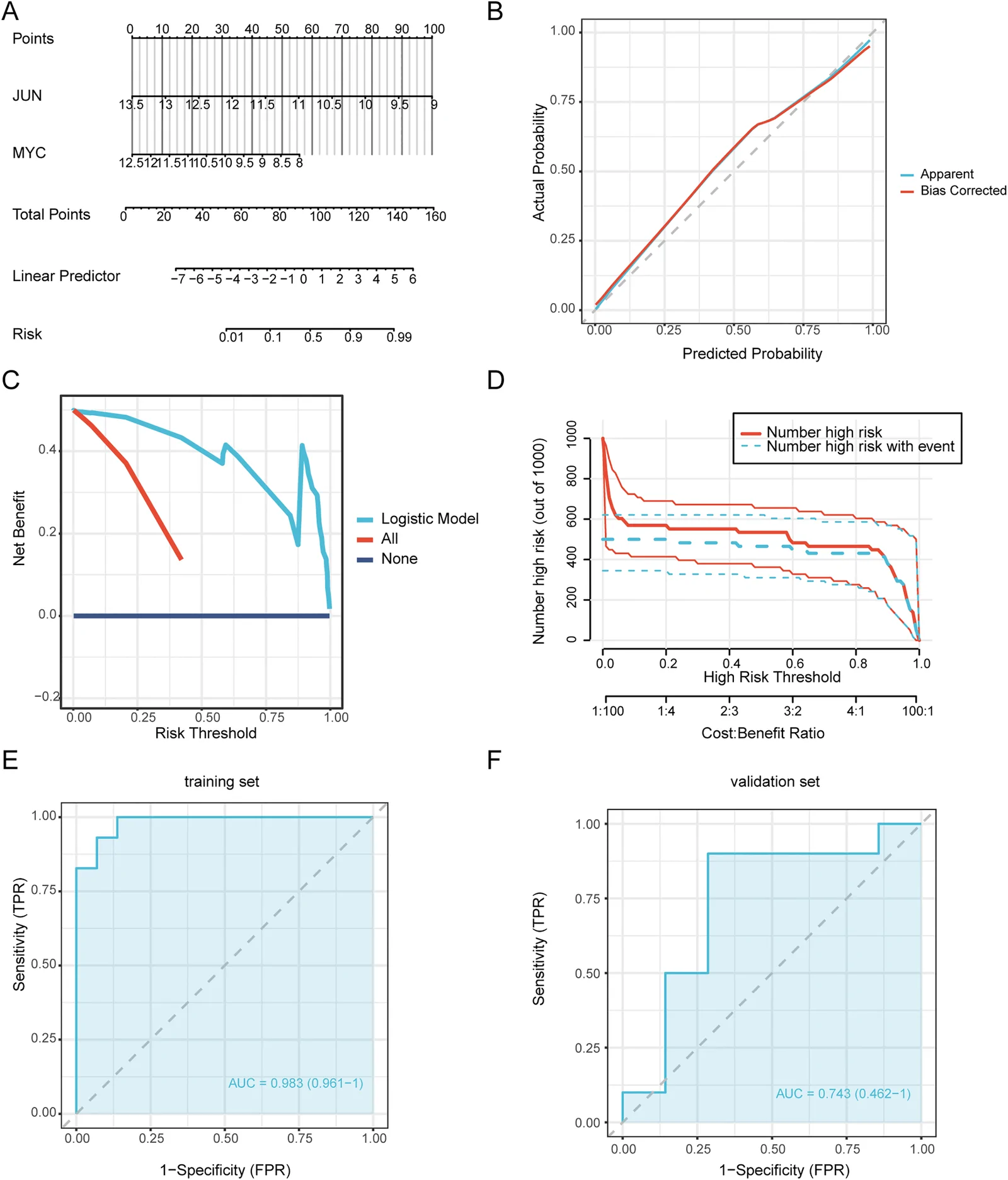
Construction and validation of nomogram model. (**A**) A nomogram was constructed to predict the prevalence of OA based on JUN and MYC. (**B**) The calibration curve showed the predictive accuracy of the nomogram model. (**C**) The DCA curve showed the benefits for patients of the nomogram model. (**D**) The clinical impact curve showed the clinical impact of the predictive model. (**E**) The ROC curve showed the predictive accuracy of the nomogram model in the training set. (**F**) The ROC curve showed the predictive accuracy of the nomogram model in the validation set (GSE82107).

Univariate regression analysis was performed on three hub genes (JUN, MYC, and NFKBIA), followed by multivariate regression analysis on the genes with p value < 0.1. Finally, JUN and MYC were determined as independent influencing factors. To assess the risk of OA, we employed the “rms” R package to develop a nomogram model utilizing two biomarkers: JUN and MYC (Fig 5A). The calibration curve served as a visual tool for evaluating the alignment between predicted outcomes and actual occurrences, revealing that the nomogram model displayed robust calibration, with the predicted probability of OA correlating well with the observed risk (Fig 5B). DCA indicated that within the threshold range of 0.1–1.0, patients utilizing this model experienced greater benefits than those who received no intervention or complete intervention (Fig 5C). Building upon the insights from the DCA, we conducted an additional assessment using the clinical impact curve. Within the high-risk threshold of 0–1.0, the “high-risk number” curve closely aligned with the “high-risk with event number” curve (Fig 5D), signifying that the nomogram model demonstrates significant predictive capability. Lastly, we generated ROC curves from both the training set and validation set (GSE82107) to evaluate the model’s predictive efficacy. An area under the ROC curve exceeding 0.7 indicates that the model has notable discriminative power and accuracy in predicting OA (Fig 5E and 5F). Consequently, the model demonstrates a strong potential for predicting OA. For detailed information, see S7 File.

### Immune microenvironment and correlation analysis

**Fig 6:**
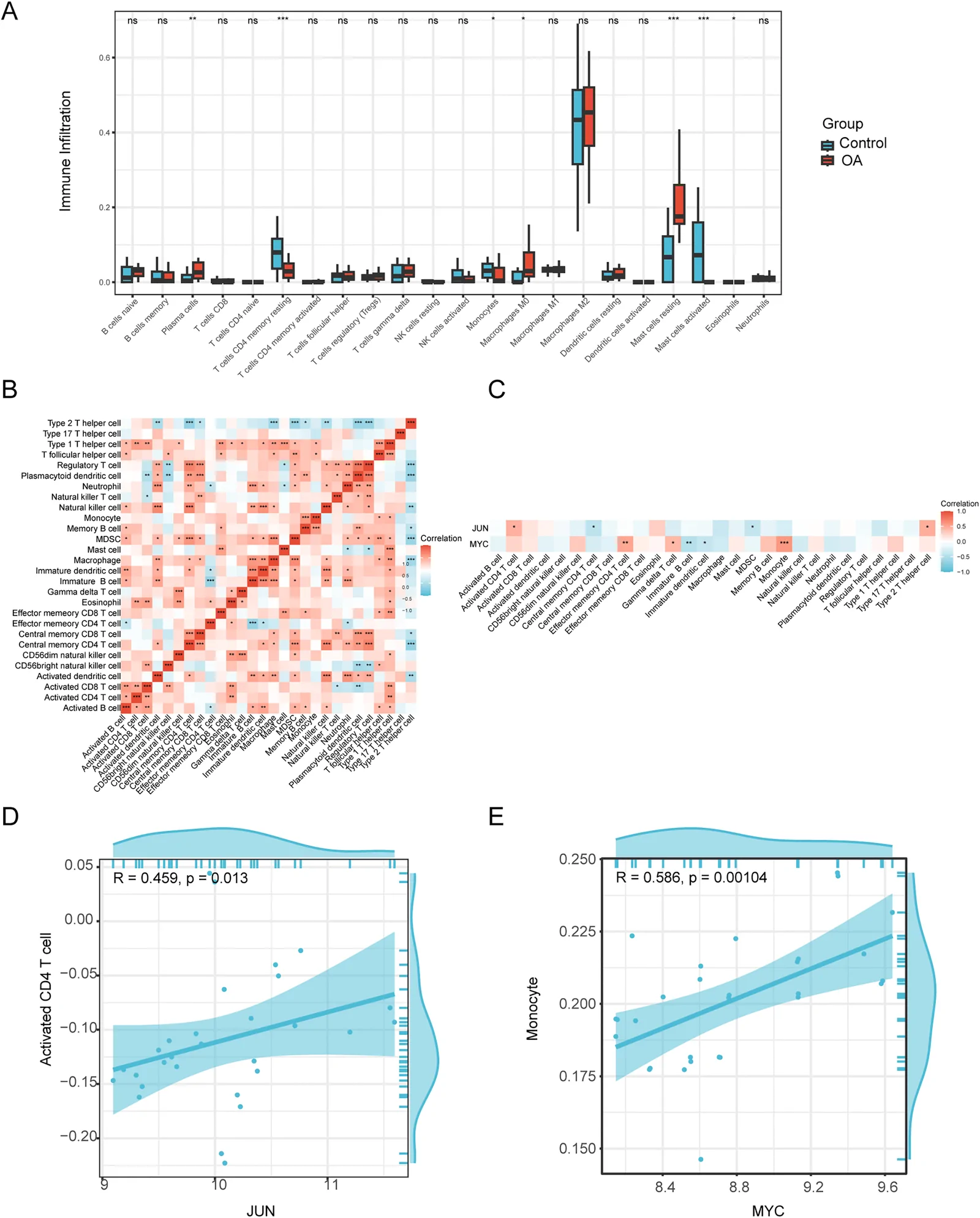
Immune cell infltration analysis and correlation analysis. (**A**) The CIBERSORT-based boxplot demonstrated a signifcant diference in immune infltration patterns between the OA and control groups. (**B**) Correlation analysis between 28 immune cell species. (**C**) The connection between immune cells and JUN, MYC. (**D**) Correlation between Activated CD4 T cell and JUN. (**E**) Correlation between Monocyte and MYC. ns, not significant, ^*^p < 0.05, ^**^p < 0.01, ^***^p < 0.001.

In addition, we employed the “CIBERSORT” algorithm to examine the variations in immune cell infiltration between the OA and control groups. The results of this analysis were illustrated through box plot (Fig 6A). The computation shows patterns of immune infiltration that are consistent with upstream analyses, our findings revealed that the OA group exhibited increased infiltration of plasma cells, M0 macrophages, and resting mast cells compared with the control group but a decrease in the infiltration of resting CD4 memory T cells, monocytes, and activated mast cells (Fig 6A). For detailed information, see S8 File. The correlation analysis results of 28 immune cells are presented in Fig 6B. To further investigate the correlation between immune infiltrating cells and biomarkers, Spearman’s correlation analysis with p < 0.05 was applied. The statistical correlations were summarized in (Fig 6C). The scatter plot revealed that JUN was positively associated with activated CD4 T cells (Fig 6D), whereas MYC was positively correlated with monocyte (Fig 6E). For detailed information, see S9 File.

### GSEA

**Fig 7:**
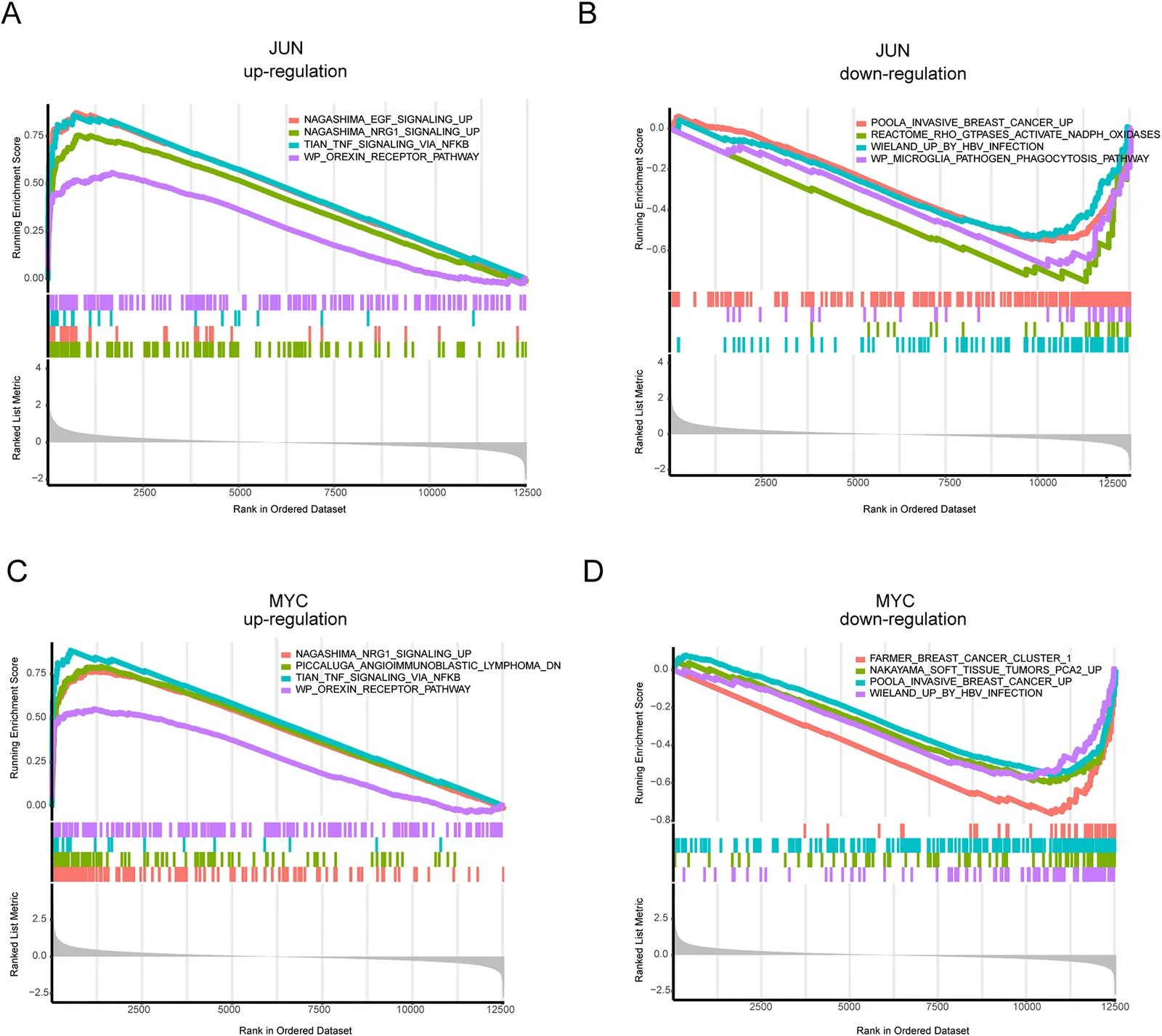
Pathway and function analysis of two biomarkers. (**A)** The four significant pathways positively associated with JUN. (**B**) The four significant pathways negatively associated with JUN. (**C**) The four significant pathways positively associated with MYC. (D) The four significant pathways negatively associated with MYC.

We applied single-gene GSEA to investigate the biological function and pathways associated with two biomarkers. According to the NES value, the four most significant down-regulated items and four up-regulated items for each biomarkers were exhibited in (Fig 7A and 7D). The results suggested that the two biomarkers might be involved in nagashima NRG1 signaling up, tian TNF signaling via NFKB, wp orexin receptor pathway and poola invasive breast cancer up. For detailed information, see S10 File.

### Experimental results

**Fig 8:**
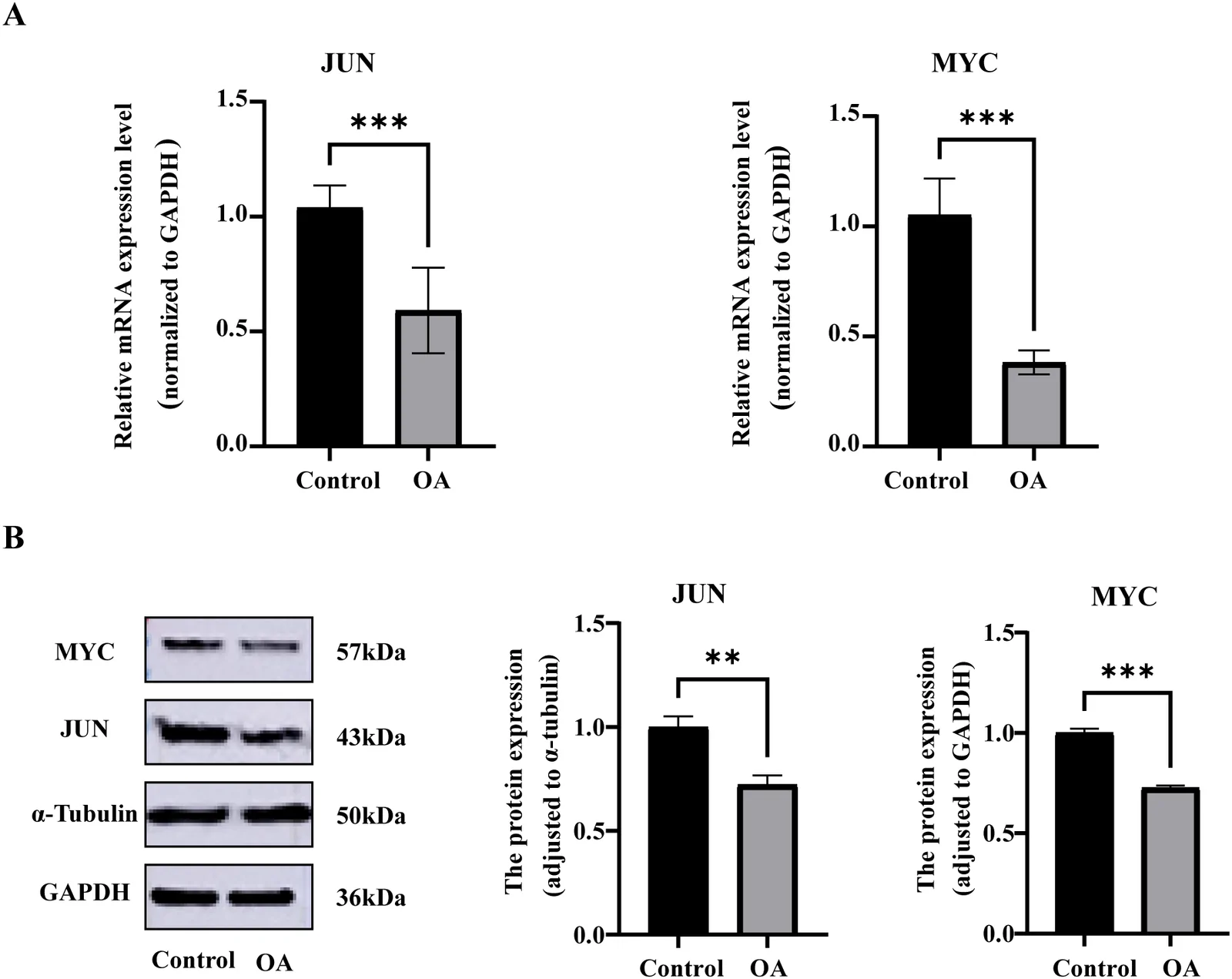
Expression levels of two biomarkers by qRT-PCR and Western blotting. (**A**) Gene expression levels of JUN and MYC by qRT-PCR. (**B**) The protein expression levels of JUN and MYC by Western blotting.

The results obtained from RT-qPCR and WB assay revealed a notable decrease in the relative expression levels of JUN and MYC in cells derived from OA, in contrast to normal chondrocytes(Fig 8A and 8B). For detailed information, see S11 File.

## Discussion

OA is a complex chronic disorder frequently characterized by synovitis and cartilage destruction [30]. However, the relationship between OA and acetylation remains unclear. Therefore, studying the expression of acetylation-related genes (For detailed information, see S12 File) and their role in the pathogenesis of OA is crucial for elucidating the pathogenic mechanisms and discovering novel biomarkers. In this study, we identified 197 ACEDEGs through bioinformatics analysis and determined MYC and JUN as candidate hub genes with potential diagnostic value for OA. These hub genes may play critical roles in the immune response and disease progression. This research not only enhances our understanding of OA pathophysiology but also provides a foundation for developing innovative diagnostic tools and targeted therapies [31,32].

The integration of bioinformatics and experimental validation in this study has unveiled significant insights into the molecular landscape of OA, particularly through the identification of acetylation-related DEGs [33]. The comprehensive analysis revealed 2,402 DEGs, with 1,185 upregulated and 1,217 downregulated genes, highlighting the complexity of OA at the gene expression level. The GO and KEGG analyses demonstrated that these DEGs are enriched in critical pathways such as MAPK, PI3K-Akt, and TNF, which are pivotal in inflammatory processes associated with OA pathology. This enrichment pattern is consistent with previous transcriptomic studies in OA, thereby supporting the robustness of our findings [34]; Meanwhile, previous studies have established the involvement of JUN and MYC in OA pathophysiology. As an example, JUN protein is a member of the AP-1 transcription factor complex and is associated with the apoptosis of chondrocytes and destruction of extracellular matrix in the cartilage of OA [35]. Similarly, it has been found that MYC controls chondrocyte proliferation and differentiation and that its aberrant expression has been linked to OA progression [36]. Notably, both JUN and MYC are subject to acetylation modifications that modulate their transcriptional activity, thereby influencing downstream target genes involved in inflammation and matrix remodeling. Consistent with our findings, several bioinformatics studies have reported downregulation of JUN and MYC in OA synovial tissue or cartilage compared to normal controls [37]. Our qRT-PCR and Western blotting results further corroborated their reduced expression in OA, aligning with these earlier observations. Furthermore, The ACEDEGs identified by us, such as FOS, EGR1, and ATF3, have been shown to be connected with OA pathology. As an illustration, FOS and EGR1 are involved in reactions of inflammation and hypertrophy of chondrocytes and ATF3 has been found to participate in synovial inflammation and cytokine synthesis [38]. These overlaps improve our confidence in our bioinformatics pipeline.

Furthermore, the study’s findings on immune cell infiltration patterns provide crucial insights into the role of the immune microenvironment in OA progression. The observed infiltration pattern of plasma cells, M0 macrophages, and resting mast cells, alongside the relative decrease in activated CD4 T cells and monocytes, indicates an inflammatory pattern rather than a direct measure of increased inflammation [39]. This pattern suggests a complex humoral and innate immune response within OA-affected joints, where plasma cells may contribute through antibody-mediated mechanisms [40]. Plasma cells may exert their functions by locally secreting specific antibodies; M0 macrophages are in a non-polarized resting state and can promote pro-inflammatory reactions after specific induction; Activated mast cells in a resting state will release various inflammatory mediators, further aggravating the inflammatory damage at the synovial site. Compared with the previous conclusions from studies in the same field, the above-mentioned immune cell action patterns are completely consistent with the characteristic chronic low-intensity immune phenotype of OA, and there is a clear difference from the common acute inflammatory response found in patients with rheumatoid arthritis [41].

Notably, the correlation of biomarkers JUN and MYC with specific immune cell populations indicates their potential roles in modulating immune responses, thereby presenting opportunities for immunomodulatory strategies as novel therapeutic approaches for OA management. These observations highlight the necessity of integrating immune profiling in future OA research to enhance the understanding of disease mechanisms and improve treatment outcomes [42,43].

The validation of the predictive nomogram model incorporating JUN and MYC, with an AUC exceeding 0.7, suggests its potential clinical possibility for early OA diagnosis and risk stratification. this model demonstrates robust predictive power, suggesting its potential utility in identifying at-risk populations who may benefit from early interventions [44,45]. The comparative performance of LASSO, RF, and XGBoost algorithms in selecting hub genes further emphasizes the strength of machine learning approaches in biomarker discovery [46]. The integration of these predictive models into clinical workflows necessitates prospective validation in diverse populations to ensure their reliability and generalizability in real-world settings [47].

Lastly, the GSEA linking JUN and MYC to pathways such as NRG1 signaling and TNF/NF-KB highlights the interconnectedness of OA with other pathological conditions, including cancer. The retention of cross-disease pathways suggests that existing therapies targeting these pathways may be worth investigating for repurposing in OA treatment, potentially accelerating the use of research findings into clinical practice [48,49].

In summary, this study’s findings not only identify JUN and MYC as key biomarkers in OA but also establish a foundation for future investigations into their functional roles in cartilage homeostasis and immune regulation [37,50,51]. The discrepancies observed between mRNA and protein levels may arise from several regulatory mechanisms, including translational efficiency, protein degradation, and post-translational modifications. These differences may reflect context-dependent regulation in OA pathogenesis, such as altered protein stability or activity under inflammatory conditions [52]. Moreover, exploring the therapeutic potential of targeting these hub genes may unveil new pathways for disease-modifying treatments that could alleviate the burden of OA on patients and healthcare systems alike [53]. The need for large validation cohorts and mechanistic studies is imperative to effectively translate these findings into clinical practice.

This study, while providing significant insights into the role of acetylation-related genes in OA, is not without its limitations. The absence of in vivo functional validation for the identified biomarkers, JUN and MYC, limits the ability to draw definitive conclusions about their mechanistic roles in OA pathogenesis. Furthermore, the sample size for validation, particularly in the GSE82107 cohort, was relatively small, comprising only 10 patients with OA and 7 controls, which may affect the generalizability of the findings. The possibility of batch effects from the integration of multiple datasets necessitates careful interpretation of the results. Future research should aim to address these limitations through large, well-controlled studies and functional assays to substantiate the biological relevance of the identified biomarkers.

## Conclusion

In conclusion, we identified JUN and MYC as novel acetylation-related biomarkers in OA via bioinformatics, highlighting the role of acetylation in OA pathogenesis. Our diagnostic model showed high diagnostic performance with an AUC of 0.983 in training and 0.743 in validation, indicating clinical reliability. Significant changes in immune cell infiltration were observed, contributing to the inflammatory processes in OA. Validation through qRT-PCR and WB supported our findings, suggesting therapeutic targets and future research on the roles of JUN and MYC in OA progression and immune dynamics. This study enhances the understanding of OA and aims to integrate biomarkers into clinical practice for improved management.

## Supporting information

S1 File Dataset curation and DEG analysis. (ZIP)

S2 File GO and KEGG enrichment analysis. (ZIP)

S3 File WGCNA analysis. (ZIP)

S4 File Screening ACEDEGs and PPI analysis. (ZIP)

S5 File 13 ACEDEGs chromosome mapping. (ZIP)

S6 File Machine-learning algorithm screening for hub genes. (ZIP)

S7 File Nomogram construction. (ZIP)

S8 File Immune microenvironment analysis. (ZIP)

S9 File Correlation analysis. (ZIP)

S10 File Gene set enrichment analysis. (ZIP)

S11 File Western blot raw images. (DOCX)

S12 File Gene_acetylation. (CSV)

## References

[1] Chen J, Deng M, Wang J, Liu Y, Hu Z, Luan F, et al. Recent advances in injectable hydrogels for osteoarthritis treatments. Front Bioeng Biotechnol. 2025;13:1644222. doi: 10.3389/fbioe.2025.1644222 40843442 PMC12364895

[2] Hu Y, Chen X, Wang S, Jing Y, Su J. Subchondral bone microenvironment in osteoarthritis and pain. Bone Res. 2021;9(1):20. doi: 10.1038/s41413-021-00147-z 33731688 PMC7969608

[3] Goudarzi R, Dehpour AR, Partoazar A. Nanomedicine and regenerative medicine approaches in osteoarthritis therapy. Aging Clin Exp Res. 2022;34(10):2305–15. doi: 10.1007/s40520-022-02199-5 35867240

[4] Kloppenburg M, Namane M, Cicuttini F. The osteoarthritis. Lancet. 2025;405(10472):71–85. doi: 10.1016/S0140-6736(24)02322-5 39755397

[5] Barnes EV, Edwards NL. Treatment of osteoarthritis. South Med J. 2005;98(2):205–9. doi: 10.1097/01.SMJ.0000153116.71823.24 15759951

[6] Nguyen LT, Sharma AR, Chakraborty C, Saibaba B, Ahn M-E, Lee S-S. Review of prospects of biological fluid biomarkers in osteoarthritis. Int J Mol Sci. 2017;18(3):601. doi: 10.3390/ijms18030601 28287489 PMC5372617

[7] Deng M, Tang C, Yin L, Jiang Y, Huang Y, Feng Y, et al. Clinical and omics biomarkers in osteoarthritis diagnosis and treatment. J Orthop Translat. 2025;50:295–305. doi: 10.1016/j.jot.2024.12.007 39911590 PMC11795539

[8] Ramos YFM, Rice SJ, Ali SA, Pastrello C, Jurisica I, Rai MF, et al. Evolution and advancements in genomics and epigenomics in OA research: how far we have come. Osteoarthritis Cartilage. 2024;32(7):858–68. doi: 10.1016/j.joca.2024.02.656 38428513

[9] Chen L-Y, Lotz M, Terkeltaub R, Liu-Bryan R. Modulation of matrix metabolism by ATP-citrate lyase in articular chondrocytes. J Biol Chem. 2018;293(31):12259–70. doi: 10.1074/jbc.RA118.002261 29929979 PMC6078460

[10] He Q, Chen Y, Huang C, Cong X, Li Y, Xiao D, et al. Exploring the potential mechanisms of acetyl tributyl citrate exposure on osteoarthritis based on novel network toxicology. Sci Rep. 2025;15(1):29363. doi: 10.1038/s41598-025-11178-5 40790309 PMC12339750

[11] Jahr H. HDACi and Nrf2: not from alpha to omega but from acetylation to OA. Arthritis Res Ther. 2015;17:381. doi: 10.1186/s13075-015-0885-x 26714479 PMC4718044

[12] Tufts L, Shet Vishnudas K, Fu E, Kurhanewicz J, Ries M, Alliston T, et al. Correlating high-resolution magic angle spinning NMR spectroscopy and gene analysis in osteoarthritic cartilage. NMR Biomed. 2015;28(5):523–8. doi: 10.1002/nbm.3285 25761416 PMC4400260

[13] Liu S, Li G, Zhu Y, Xu C, Yang Q, Xiong A, et al. Analysis of gut microbiome composition, function, and phenotype in patients with osteoarthritis. Front Microbiol. 2022;13:980591. doi: 10.3389/fmicb.2022.980591 36504782 PMC9732244

[14] Bi S, Han B, Fan H, Liu Y, Cui X. Mitochondria-related gene MAOB is a key biomarker of osteoarthritis and inhibition of its expression reduces LPS-induced chondrocyte damage. Biochem Genet. 2024;62(3):2314–31. doi: 10.1007/s10528-023-10486-7 37651071

[15] Sun X, Duan H, Xiao L, Yao S, He Q, Chen X, et al. Identification of key genes in osteoarthritis using bioinformatics, principal component analysis and meta-analysis. Exp Ther Med. 2021;21(1):18. doi: 10.3892/etm.2020.9450 33235627 PMC7678638

[16] Xie B, Zhou X, Qiu J. Identification of novel potential biomarkers in infantile hemangioma via weighted gene co-expression network analysis. BMC Pediatr. 2022;22(1):239. doi: 10.1186/s12887-022-03306-1 35501731 PMC9063075

[17] Cao F, Fan YC, Yu YH, Yang GH, Zhong H. Dissecting prognosis modules and biomarkers in glioblastoma based on weighted gene co-expression network analysis. Cancer Manag Res. 2021;13:5477–89. doi: 10.2147/Cmar.S310346 34267555 PMC8276137

[18] Okuyan HM, Coşkun A, Begen MA. Current status, opportunities, and challenges of exosomes in diagnosis and treatment of osteoarthritis. Life Sci. 2025;362:123365. doi: 10.1016/j.lfs.2024.123365 39761740

[19] Weber AE, Bolia IK, Trasolini NA. Biological strategies for osteoarthritis: from early diagnosis to treatment. Int Orthop. 2021;45(2):335–44. doi: 10.1007/s00264-020-04838-w 33078204

[20] Arnscheidt C, Meder A, Rolauffs B. Early diagnosis of osteoarthritis: clinical reality and promising experimental techniques. Z Orthop Unfall. 2016;154(3):254–68. doi: 10.1055/s-0042-100478 26894867

[21] Jamshidi A, Pelletier J-P, Martel-Pelletier J. Machine-learning-based patient-specific prediction models for knee osteoarthritis. Nat Rev Rheumatol. 2019;15(1):49–60. doi: 10.1038/s41584-018-0130-5 30523334

[22] Segarra-Queralt M, Neidlin M, Tio L, Monfort J, Monllau JC, González Ballester MÁ, et al. Regulatory network-based model to simulate the biochemical regulation of chondrocytes in healthy and osteoarthritic environments. Sci Rep. 2022;12(1):3856. doi: 10.1038/s41598-022-07776-2 35264634 PMC8907219

[23] Mobasheri A, Thudium CS, Bay-Jensen A-C, Maleitzke T, Geissler S, Duda GN, et al. Biomarkers for osteoarthritis: Current status and future prospects. Best Pract Res Clin Rheumatol. 2023;37(2):101852. doi: 10.1016/j.berh.2023.101852 37620236

[24] Musumeci G, Castrogiovanni P, Trovato FM, Weinberg AM, Al-Wasiyah MK, Alqahtani MH, et al. Biomarkers of chondrocyte apoptosis and autophagy in osteoarthritis. Int J Mol Sci. 2015;16(9):20560–75. doi: 10.3390/ijms160920560 26334269 PMC4613218

[25] Liao C-S, He F-Z, Li X-Y, Zhang Y, Han P-F. Analysis of common differential gene expression in synovial cells of osteoarthritis and rheumatoid arthritis. PLoS One. 2024;19(5):e0303506. doi: 10.1371/journal.pone.0303506 38771826 PMC11108184

[26] Zhang Y, Zhu T, He F, Chen AC, Yang H, Zhu X. Identification of key genes and pathways in osteoarthritis via bioinformatic tools: an updated analysis. Cartilage. 2021;13(1_suppl):1457S–1464S. doi: 10.1177/19476035211008975 33855867 PMC8808887

[27] Hu H-J, Kuang C, Deng R-L, Zheng Z-J, Liu K-Y, Wei X-X. Identification of susceptibility modules and characteristic genes to osteoarthritis by WGCNA. Ann Biol Clin (Paris). 2024;82(4):423–37. doi: 10.1684/abc.2024.1913 39297544

[28] Wang Y, Li Z, Xu X, Li X, Huang R, Wu G. Construction and validation of a senescence-related gene signature for early prediction and treatment of osteoarthritis based on bioinformatics analysis. Sci Rep. 2024;14(1):31862. doi: 10.1038/s41598-024-83268-9 39738612 PMC11686076

[29] Wu Z-Y, Du G, Lin Y-C. Identifying hub genes and immune infiltration of osteoarthritis using comprehensive bioinformatics analysis. J Orthop Surg Res. 2021;16(1):630. doi: 10.1186/s13018-021-02796-6 34670585 PMC8527722

[30] Dinç M, Bayrak HÇ, Karasu R, Aykaç B, Soydemir ÖC, Saricetin A. Intra-articular N-acetylcysteine reduces synovitis without preventing cartilage degeneration in experimental osteoarthritis. Biomedicines. 2025;14(1):86. doi: 10.3390/biomedicines14010086 41595622 PMC12839232

[31] Sun C, Teng F, Xia Y. Extracellular vesicles in osteoarthritis: mechanisms, therapeutic potential, and diagnostic applications. Front Immunol. 2025;16:1595095. doi: 10.3389/fimmu.2025.1595095 40881706 PMC12380537

[32] Shakeri M, Aminian A, Mokhtari K, Bahaeddini M, Tabrizian P, Farahani N, et al. Unraveling the molecular landscape of osteoarthritis: a comprehensive review focused on the role of non-coding RNAs. Pathol Res Pract. 2024;260:155446. doi: 10.1016/j.prp.2024.155446 39004001

[33] Cao S, Wei Y, Yue Y, Wang D, Xiong A, Yang J, et al. Bioinformatics identification and experimental verification of disulfidptosis-related genes in the progression of osteoarthritis. Biomedicines. 2024;12(8):1840. doi: 10.3390/biomedicines12081840 39200304 PMC11351109

[34] Peng H, Lin H. Integrative analysis of microRNA-320a-related genes in osteoarthritis cartilage. Front Surg. 2023;9:1005243. doi: 10.3389/fsurg.2022.1005243 36700022 PMC9869261

[35] Shi L, Xu X, Meng B, He K, Sun Y, Tong J, et al. Neuregulin 4 attenuates osteoarthritis progression by inhibiting inflammation and apoptosis of chondrocytes in mice. Calcif Tissue Int. 2022;110(1):131–42. doi: 10.1007/s00223-021-00897-2 34383111

[36] Jiang K, Jiang T, Chen Y, Mao X. Mesenchymal stem cell-derived exosomes modulate chondrocyte glutamine metabolism to alleviate osteoarthritis progression. Mediators Inflamm. 2021;2021:2979124. doi: 10.1155/2021/2979124 34992497 PMC8724850

[37] Xu W, Wang X, Liu D, Lin X, Wang B, Xi C, et al. Identification and validation of hub genes and potential drugs involved in osteoarthritis through bioinformatics analysis. Front Genet. 2023;14:1117713. doi: 10.3389/fgene.2023.1117713 36845391 PMC9947480

[38] Sanchez C, Hemmer K, Krömmelbein N, Seilheimer B, Dubuc J-E, Antoine C, et al. Reduction of matrix metallopeptidase 13 and promotion of chondrogenesis by Zeel T in primary human osteoarthritic chondrocytes. Front Pharmacol. 2021;12:635034. doi: 10.3389/fphar.2021.635034 34045958 PMC8144641

[39] Zhang Q, Sun C, Liu X, Zhu C, Ma C, Feng R. Mechanism of immune infiltration in synovial tissue of osteoarthritis: a gene expression-based study. J Orthop Surg Res. 2023;18(1):58. doi: 10.1186/s13018-023-03541-x 36681837 PMC9862811

[40] Meng J, Du H, Lv H, Lu J, Li J, Yao J. Identification of the osteoarthritis signature gene PDK1 by machine learning and its regulatory mechanisms on chondrocyte autophagy and apoptosis. Front Immunol. 2023;13:1072526. doi: 10.3389/fimmu.2022.1072526 36685513 PMC9853447

[41] Zhang Z, Shao Z, Xu Z, Wang J. Similarities and differences between osteoarthritis and rheumatoid arthritis: insights from Mendelian randomization and transcriptome analysis. J Transl Med. 2024;22(1):851. doi: 10.1186/s12967-024-05643-4 39304950 PMC11414073

[42] Moulin D, Sellam J, Berenbaum F, Guicheux J, Boutet M-A. The role of the immune system in osteoarthritis: mechanisms, challenges and future directions. Nat Rev Rheumatol. 2025;21(4):221–36. doi: 10.1038/s41584-025-01223-y 40082724

[43] Giorgino R, Albano D, Fusco S, Peretti GM, Mangiavini L, Messina C. Knee osteoarthritis: epidemiology, pathogenesis, and mesenchymal stem cells: what else is new? An update. Int J Mol Sci. 2023;24(7):6405. doi: 10.3390/ijms24076405 37047377 PMC10094836

[44] Zhang M, Zhang J, Cui Y, Xing Z. Predictive power of lipid-related indicators for testosterone deficiency: a comparative analysis, NHANES 2011-2016. Int Urol Nephrol. 2024;56(6):1825–33. doi: 10.1007/s11255-023-03935-0 38280934

[45] Hallinan R, Ray J, Byrne A, Agho K, Attia J. Therapeutic thresholds in methadone maintenance treatment: a receiver operating characteristic analysis. Drug Alcohol Depend. 2006;81(2):129–36. doi: 10.1016/j.drugalcdep.2005.06.005 16026938

[46] Zhu Y, Liu J, Wang B. Integrated approach of machine learning, Mendelian randomization and experimental validation for biomarker discovery in diabetic nephropathy. Diabetes Obes Metab. 2024;26(12):5646–60. doi: 10.1111/dom.15933 39370621

[47] Gurumurthy G, Kisiel F, Reynolds L, Thomas W, Othman M, Arachchillage DJ. Machine learning in venous thromboembolism - why and what next? Semin Thromb Hemost. 2025. doi: 10.1055/a-2669-7933 40829629

[48] Lin Y, Wu J, Wang P, Lin X, Yang H. Identification of key genes related to lymphangiogenesis in venous thromboembolism through transcriptomics and verification by RT-qPCR. Front Med (Lausanne). 2025;12:1659881. doi: 10.3389/fmed.2025.1659881 41210882 PMC12592171

[49] He S, He L, Yan F, Li J, Liao X, Ling M, et al. Identification of hub genes associated with acute kidney injury induced by renal ischemia-reperfusion injury in mice. Front Physiol. 2022;13:951855. doi: 10.3389/fphys.2022.951855 36246123 PMC9557154

[50] Rong G, Zhang Z, Zhan W, Chen M, Ruan J, Shen C. VEGFA, MYC, and JUN are abnormally elevated in the synovial tissue of patients with advanced osteoarthritis. Sci Rep. 2025;15(1):2066. doi: 10.1038/s41598-024-80551-7 39814733 PMC11736073

[51] Chen H, Ni Q, Li B, Chen L. Identification of differentially expressed genes in synovial tissue of osteoarthritis based on a more robust integrative analysis method. Clin Rheumatol. 2021;40(9):3745–54. doi: 10.1007/s10067-021-05649-z 33677723

[52] Rai MF, Collins KH, Lang A, Maerz T, Geurts J, Ruiz-Romero C, et al. Three decades of advancements in osteoarthritis research: insights from transcriptomic, proteomic, and metabolomic studies. Osteoarthritis Cartilage. 2024;32(4):385–97. doi: 10.1016/j.joca.2023.11.019 38049029 PMC12239761

[53] Zhao Y, Ou Q, Cai Y, Ruan G, Zhang Y, Ding C. Shedding light on experimental intra-articular drugs for treating knee osteoarthritis. Expert Opin Investig Drugs. 2023;32(6):509–24. doi: 10.1080/13543784.2023.2225214 37310287

