# Supplementary material for: Exploration of acetylation-related biomarkers in osteoarthritis through bioinformatics analysis
Source: PLoS One. 2026 Aug 28;21(8):e0357231. doi: 10.1371/journal.pone.0357231 (PMC13524278; doi:10.1371/journal.pone.0357231)

The original, uncropped, minimally adjusted Western blot images for Fig8.

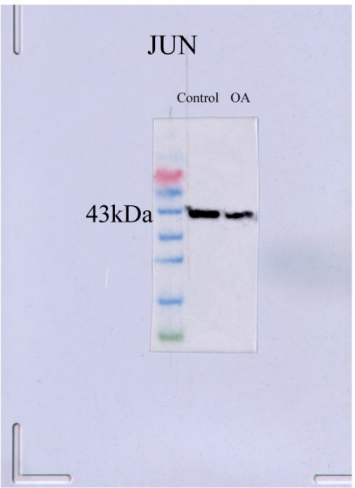

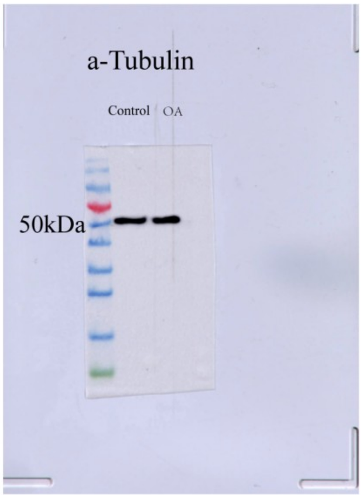

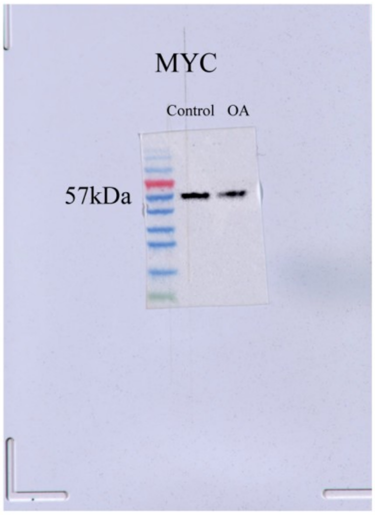

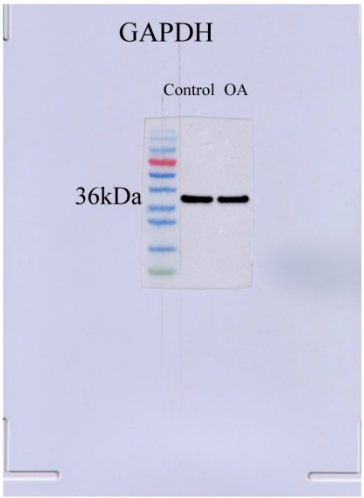

Supplement: Supplementary file 1 [file pone.0357231.s011.docx]
