# Supplementary material for: Exploration of acetylation-related biomarkers in osteoarthritis through bioinformatics analysis
Source: PLoS One. 2026 Aug 28;21(8):e0357231. doi: 10.1371/journal.pone.0357231 (PMC13524278; doi:10.1371/journal.pone.0357231)
Supplement: Supplementary file 3 [file pone.0357231.s014.docx]

Dear Editor,

Thank you very much for your careful evaluation of our revised manuscript and for the additional comments. We have thoroughly addressed each point raised by the Academic Editor. Below is a point-by-point response, and all revisions are marked in the revised manuscript using track changes.

Response to Journal Requirements

1. **If the reviewer comments include a recommendation to cite specific previously published works, please review and evaluate these publications to determine whether they are relevant and should be cited. There is no requirement to cite these works unless the editor has indicated otherwise.**

Author response: We have carefully reviewed the reviewer comments and confirm that no specific previously published works were recommended for citation. Nevertheless, we re-evaluated all references cited in our manuscript to ensure their relevance and accuracy.

2. **Please review your reference list to ensure that it is complete and correct. If you have cited papers that have been retracted, please include the rationale for doing so in the manuscript text, or remove these references and replace them with relevant current references. Any changes to the reference list should be mentioned in the rebuttal letter that accompanies your revised manuscript. If you need to cite a retracted article, indicate the article’s retracted status in the References list and also include a citation and full reference for the retraction notice.**

Author response: We have thoroughly verified the entire reference list. During this verification process, we identified that reference #25 required correction; we have accordingly replaced it with a more appropriate and relevant citation that directly supports the corresponding statement in the text. The change to the reference list are clearly marked in the revised manuscript using track changes.

**Additional Editor Comments**

**Concern 1: There are some repetitive parts/text in Discussion e.g., lines 287-288 & 290-291; Lines 298-299 & 301-04. Please remove any redundancy.**

Author response: We thank the Editor for identifying the redundant passages. We have carefully revised the Discussion to remove duplicate statements:

The overlapping text at lines 287–288 and 290–291 has been merged into one concise statement. The revised Discussion now presents each point only once, with improved clarity. These changes are located at lines 287–289 in the revised manuscript.

In the revised manuscript, we have deleted the redundant text at lines 301–304, as it repeated information already presented in lines 296–298.

**Concern 2: Please compare these DEGs with that of previous studies' was not fully addressed in my opinion; please discuss some further relevant studies on the shortlisted DEGs.**

Author response: Thank you for your constructive comment. We agree that a direct comparison between our identified DEGs and those from previous studies is essential to strengthen the manuscript. Accordingly, we have revised the Discussion section to further discuss relevant studies on the shortlisted DEGs (JUN and MYC).

Specifically, we now cite previous studies demonstrating the involvement of JUN in chondrocyte apoptosis and extracellular matrix degradation via the AP-1 transcription factor complex, and MYC in regulating chondrocyte proliferation and differentiation, with aberrant expression linked to OA progression. We also emphasize that both genes are subject to acetylation modifications, which adds an epigenetic layer to their regulation in OA. Importantly, we note that several bioinformatics studies have also reported downregulation of JUN and MYC in OA tissues, which is consistent with our qRT-PCR and Western blotting results. In addition, we now discuss other acetylation-related DEGs identified in our study (FOS, EGR1, and ATF3) that have been associated with OA pathology, reinforcing the reliability of our analytical pipeline.

These revisions are marked in the revised manuscript (lines 300–315). We hope this adequately addresses your concern.

**Concern #3: R scripts that are provided in separate suppl files could have been provided as a single file (or maybe on a GitHub or zenodo link) where a proper comment section is added for every step e.g. dataset curation, merging, processing, DGE analysis, and so on and please write complementary code comments in English. Though you can keep them as separate files, please provide comments in R scripts in English.**

Author response: Thank you for your valuable suggestion regarding the organization of our R scripts. We fully understand the importance of clear, reproducible code. However, given that our bioinformatics analysis consists of several relatively independent analytical modules , we have chosen to keep the scripts organized in separate folders corresponding to each analytical step. This modular structure allows readers to navigate and execute specific analyses more easily, without having to run the entire pipeline as a single block of code.

In accordance with the journal’s guidelines, we have ensured that all R scripts are now accompanied by detailed explanatory comments written in English.

Thank you again for this helpful comment. We hope the revised scripts are now acceptable.

**Concern #4: To ensure your figures meet our technical requirements, please review our figure guidelines: https://journals.plos.org/plosone/s/figures**

**You may also use PLOS’s free figure tool, NAAS, to help you prepare publication quality figures: https://journals.plos.org/plosone/s/figures#loc-tools-for-figure-preparation.**

**NAAS will assess whether your figures meet our technical requirements by comparing each figure against our figure specifications.**

Author response: We have carefully reviewed the PLOS ONE figure guidelines at the provided link. We have checked and adjusted all figures in our manuscript to meet the required technical specifications, including resolution (minimum 300 dpi for grayscale and color), file format (TIFF ), and proper labeling.

We have also utilized the PLOS’s NAAS tool as recommended. We uploaded all figures to NAAS, and based on its assessment, we made necessary corrections to ensure each figure meets the technical specifications. All figures now pass the NAAS quality check.

Sincerely,

Jun Yao, PhD

Bone and Joint Surgery, The First Affiliated Hospital of Guangxi Medical University, Nanning, Guangxi, China

E-mail:yaojun800524@126.com,

2026-8-5
