# Supplementary material for: Exploration of acetylation-related biomarkers in osteoarthritis through bioinformatics analysis
Source: PLoS One. 2026 Aug 28;21(8):e0357231. doi: 10.1371/journal.pone.0357231 (PMC13524278; doi:10.1371/journal.pone.0357231)
Supplement: Supplementary file 4 [file pone.0357231.s001.zip › S1. File. dataset curation and DEG analysis/output/vocano_GEO_limma.pdf]

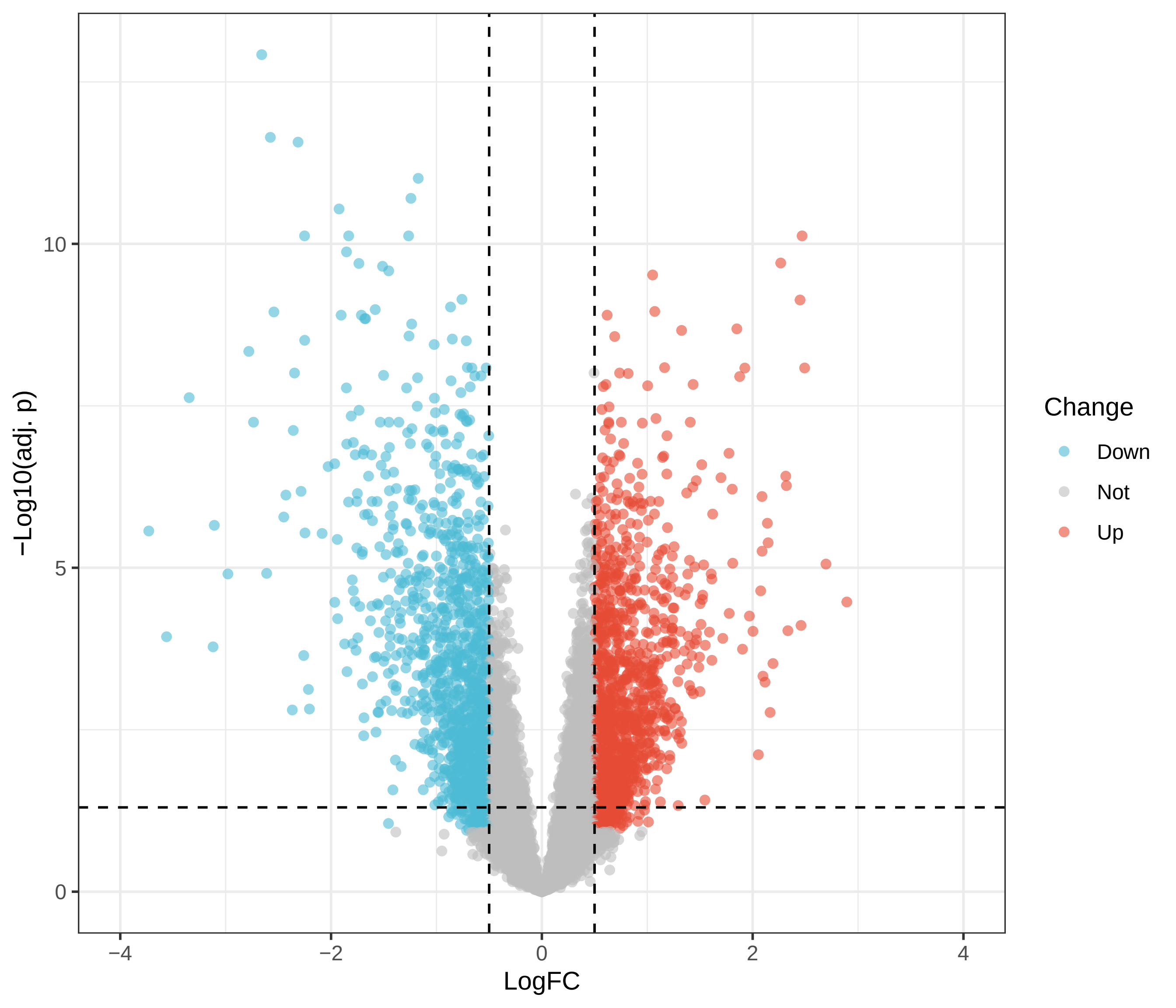

10
5
0
−Log10(adj. p)
−4
−2
0
2
4
LogFC
Change
Down
Not
Up
