# Supplementary material for: Exploration of acetylation-related biomarkers in osteoarthritis through bioinformatics analysis
Source: PLoS One. 2026 Aug 28;21(8):e0357231. doi: 10.1371/journal.pone.0357231 (PMC13524278; doi:10.1371/journal.pone.0357231)
Supplement: Supplementary file 5 [file pone.0357231.s002.zip › S2. File. GO and KEGG enrichment analysis/output/GO_dotplot.pdf]

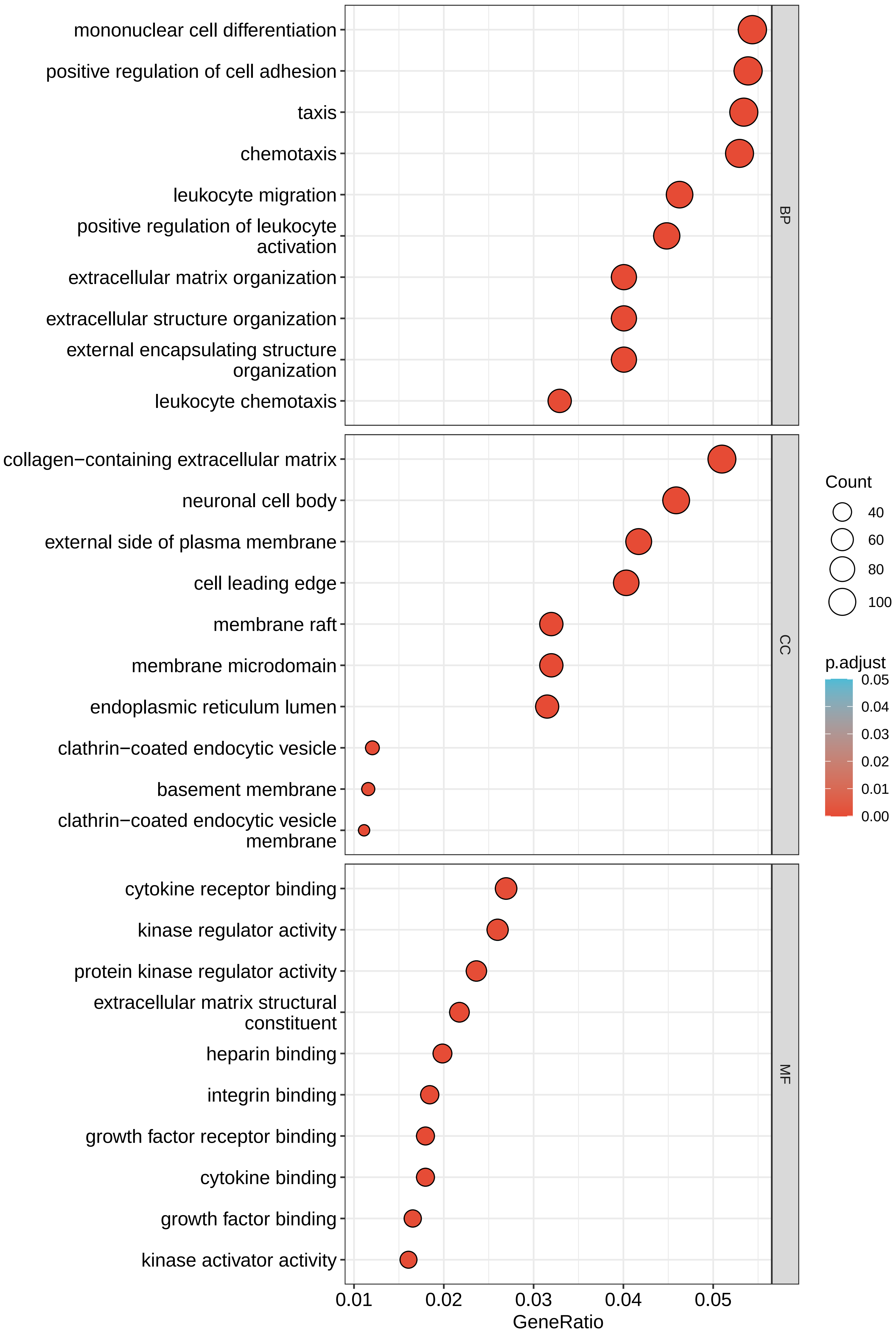

mononuclear cell differentiation
positive regulation of cell adhesion
taxis
chemotaxis
leukocyte migration
positive regulation of leukocyte activation
extracellular matrix organization
extracellular structure organization
external encapsulating structure organization
leukocyte chemotaxis
BP
collagen−containing extracellular matrix
neuronal cell body
external side of plasma membrane
cell leading edge
membrane raft
membrane microdomain
endoplasmic reticulum lumen
clathrin−coated endocytic vesicle
basement membrane
clathrin−coated endocytic vesicle membrane
CC
cytokine receptor binding
kinase regulator activity
protein kinase regulator activity
extracellular matrix structural constituent
heparin binding
integrin binding
growth factor receptor binding
cytokine binding
growth factor binding
kinase activator activity
MF
0.01
0.02
0.03
0.04
0.05
GeneRatio
Count
40
60
80
100
p.adjust
0.05
0.04
0.03
0.02
0.01
0.00
