# Supplementary material for: Exploration of acetylation-related biomarkers in osteoarthritis through bioinformatics analysis
Source: PLoS One. 2026 Aug 28;21(8):e0357231. doi: 10.1371/journal.pone.0357231 (PMC13524278; doi:10.1371/journal.pone.0357231)
Supplement: Supplementary file 5 [file pone.0357231.s002.zip › S2. File. GO and KEGG enrichment analysis/output/GO_logFC_circle.pdf]

GO

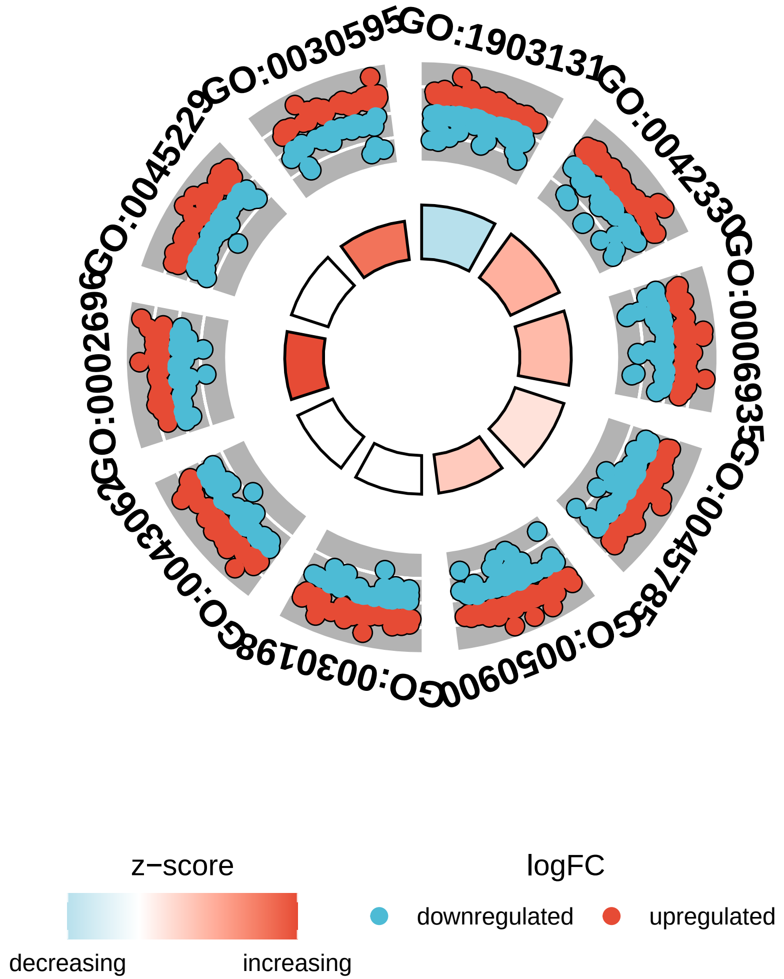

ID	Description
GO:1903131	mononuclear cell differentiation
GO:0042330	taxis
GO:0006935	chemotaxis
GO:0045785	positive regulation of cell adhesion
GO:0050900	leukocyte migration
GO:0030198	extracellular matrix organization
GO:0043062	extracellular structure organization
GO:0002696	positive regulation of leukocyte activation
GO:0045229	external encapsulating structure organization
GO:0030595	leukocyte chemotaxis
