# Supplementary material for: Exploration of acetylation-related biomarkers in osteoarthritis through bioinformatics analysis
Source: PLoS One. 2026 Aug 28;21(8):e0357231. doi: 10.1371/journal.pone.0357231 (PMC13524278; doi:10.1371/journal.pone.0357231)
Supplement: Supplementary file 5 [file pone.0357231.s002.zip › S2. File. GO and KEGG enrichment analysis/output/KEGG_barplot.pdf]

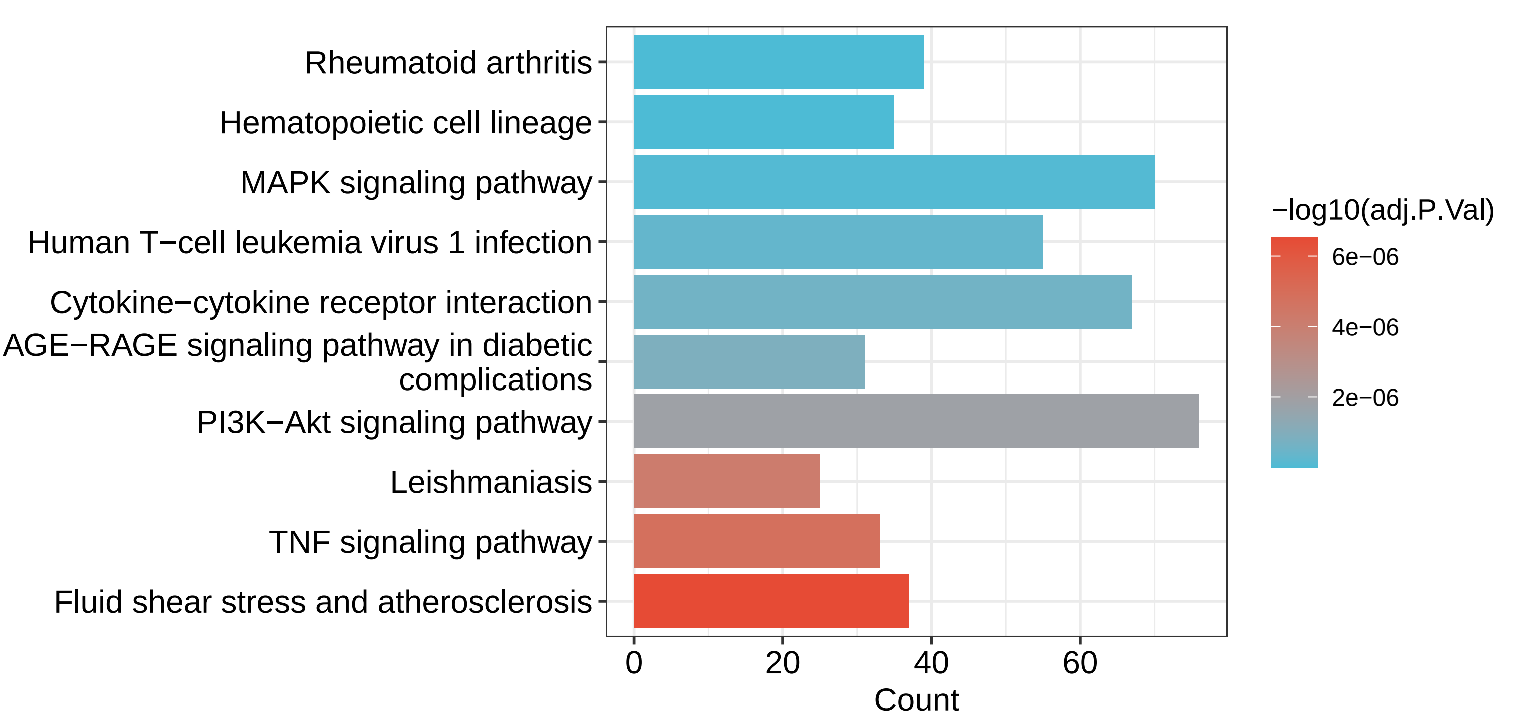

Rheumatoid arthritis
Hematopoietic cell lineage
MAPK signaling pathway
Human T−cell leukemia virus 1 infection
Cytokine−cytokine receptor interaction
AGE−RAGE signaling pathway in diabetic complications
PI3K−Akt signaling pathway
Leishmaniasis
TNF signaling pathway
Fluid shear stress and atherosclerosis
0
20
40
60
Count
−log10(adj.P.Val)
6e−06
4e−06
2e−06
