# Supplementary material for: Exploration of acetylation-related biomarkers in osteoarthritis through bioinformatics analysis
Source: PLoS One. 2026 Aug 28;21(8):e0357231. doi: 10.1371/journal.pone.0357231 (PMC13524278; doi:10.1371/journal.pone.0357231)
Supplement: Supplementary file 6 [file pone.0357231.s003.zip › S3. File. WGCNA analysis/output/WGCNA_eigengenes_cluster.pdf]

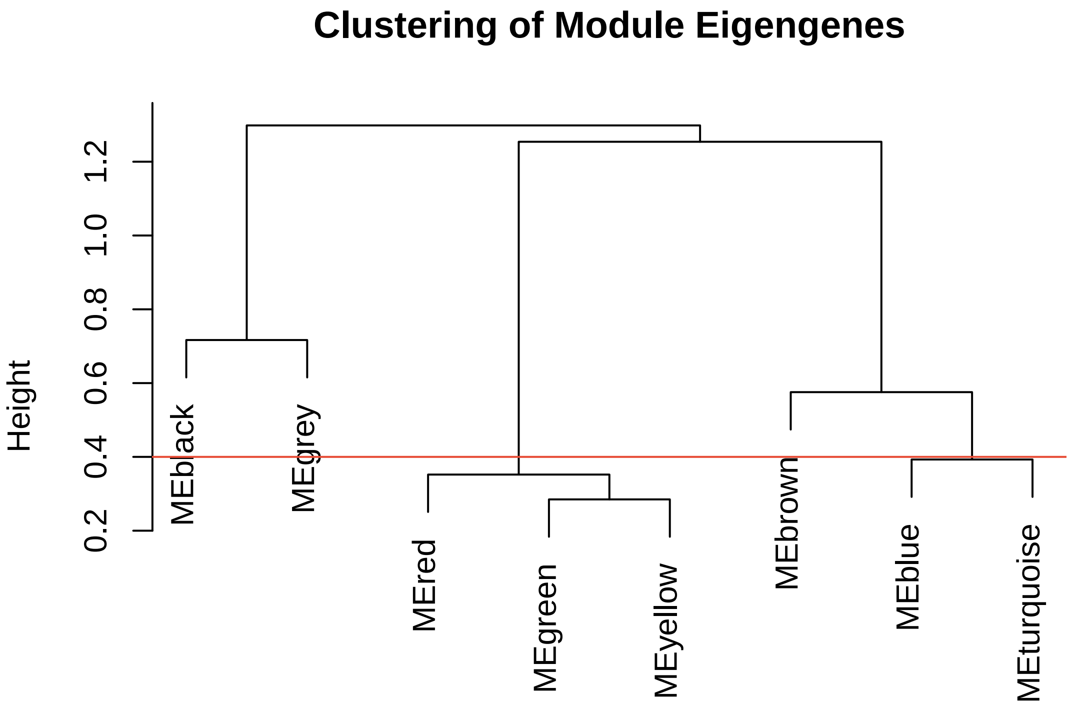

Clustering of Module Eigengenes
Height
0.2
0.4
0.6
0.8
1.0
1.2
MEblack
MEgrey
MEred
MEgreen
MEyellow
MEbrown
MEblue
MEturquoise
