# Supplementary material for: Exploration of acetylation-related biomarkers in osteoarthritis through bioinformatics analysis
Source: PLoS One. 2026 Aug 28;21(8):e0357231. doi: 10.1371/journal.pone.0357231 (PMC13524278; doi:10.1371/journal.pone.0357231)
Supplement: Supplementary file 6 [file pone.0357231.s003.zip › S3. File. WGCNA analysis/output/WGCNA_heatmap.pdf]

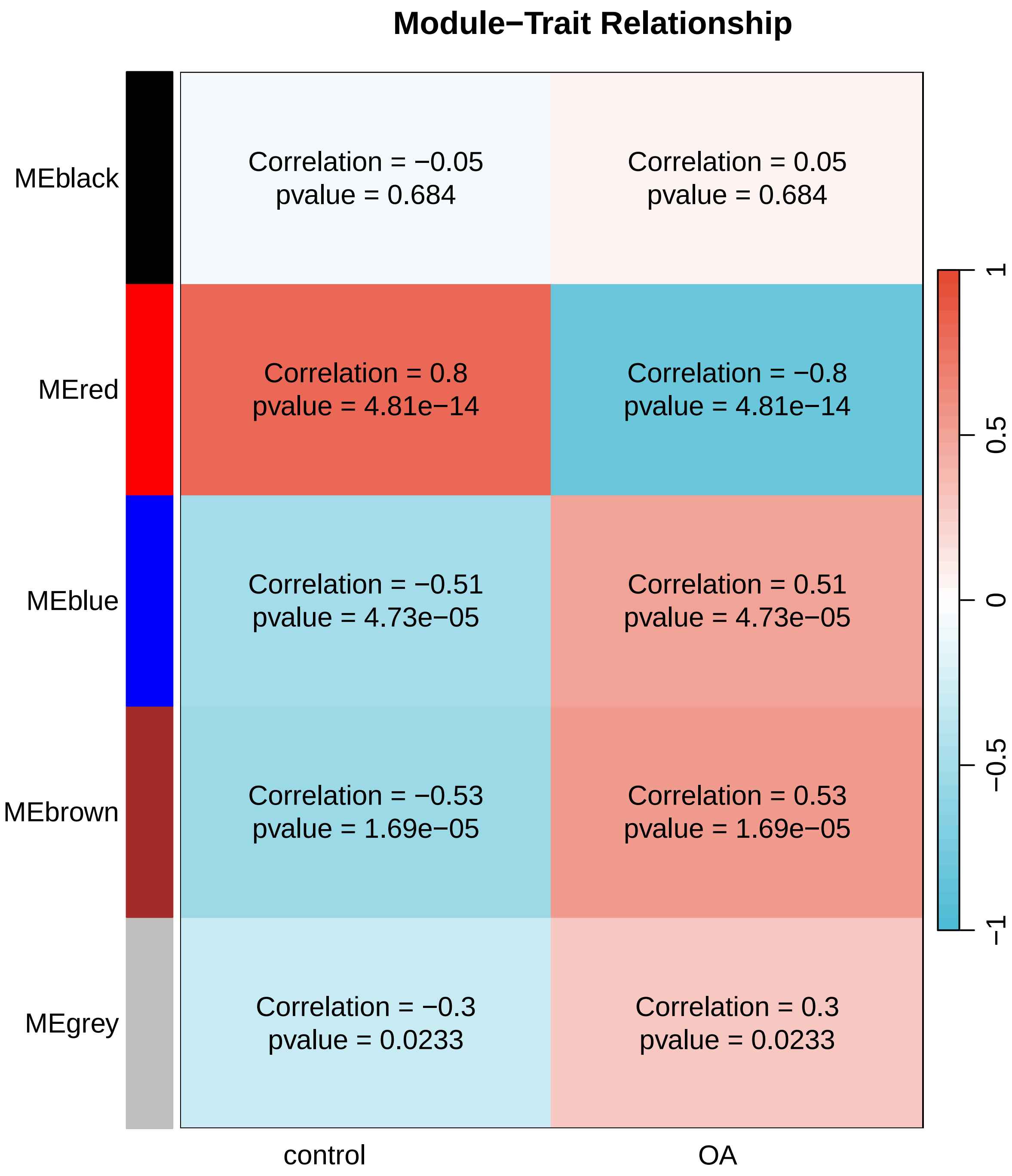
Module−Trait Relationship
MEblack
MEred
MEblue
MEbrown
MEgrey
Correlation = −0.05
pvalue = 0.684
Correlation = 0.05
pvalue = 0.684
Correlation = 0.8
pvalue = 4.81e−14
Correlation = −0.8
pvalue = 4.81e−14
Correlation = −0.51
pvalue = 4.73e−05
Correlation = 0.51
pvalue = 4.73e−05
Correlation = −0.53
pvalue = 1.69e−05
Correlation = 0.53
pvalue = 1.69e−05
Correlation = −0.3
pvalue = 0.0233
Correlation = 0.3
pvalue = 0.0233
control
OA
1
0.5
0
−0.5
−1
