# Supplementary material for: Exploration of acetylation-related biomarkers in osteoarthritis through bioinformatics analysis
Source: PLoS One. 2026 Aug 28;21(8):e0357231. doi: 10.1371/journal.pone.0357231 (PMC13524278; doi:10.1371/journal.pone.0357231)
Supplement: Supplementary file 6 [file pone.0357231.s003.zip › S3. File. WGCNA analysis/output/WGCNA_scatterplot.pdf]

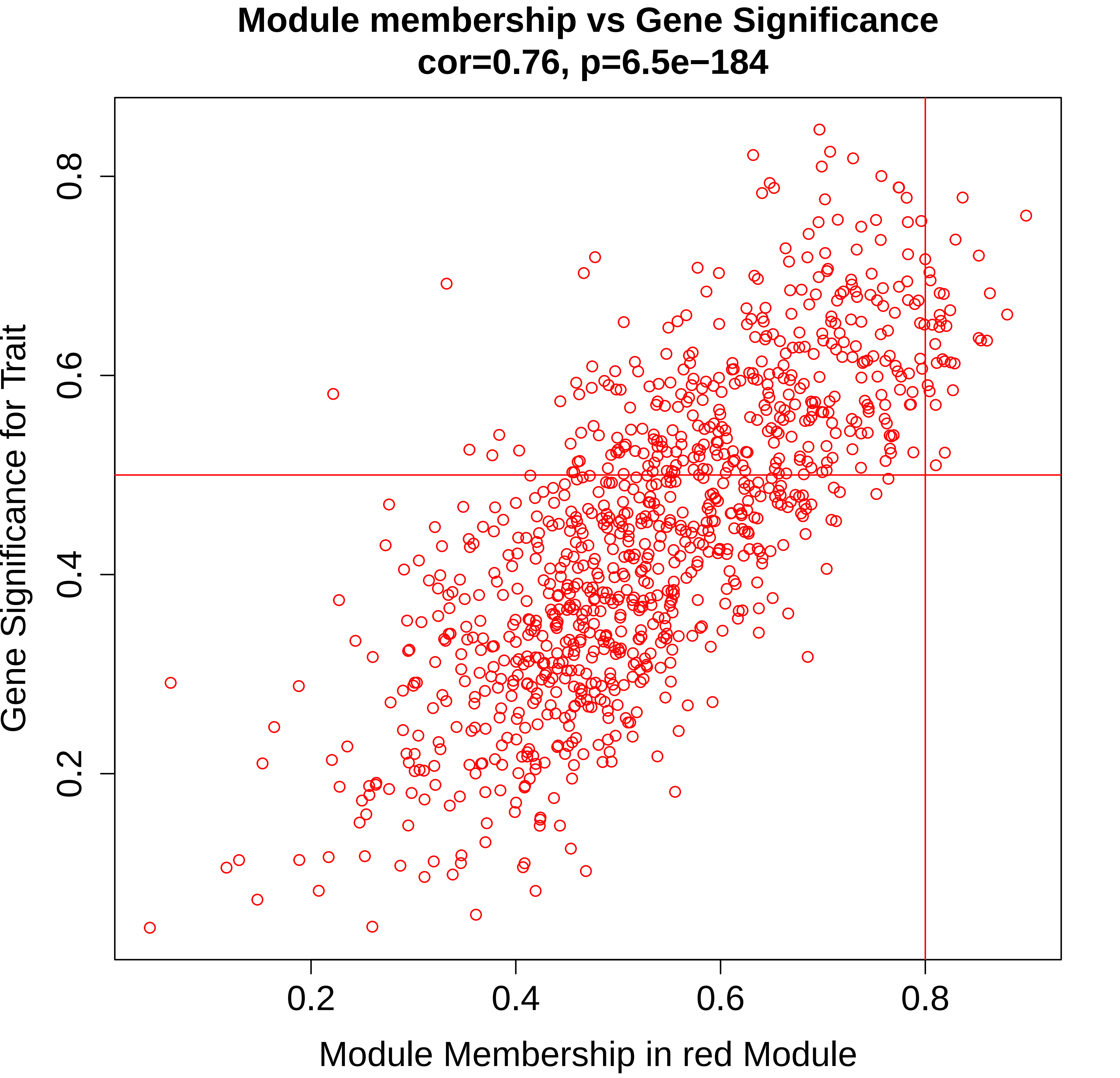

Module membership vs Gene Significance
cor=0.76, p=6.5e−184
Gene Significance for Trait
0.2
0.4
0.6
0.8
Module Membership in red Module
0.2
0.4
0.6
0.8
