# Supplementary material for: Exploration of acetylation-related biomarkers in osteoarthritis through bioinformatics analysis
Source: PLoS One. 2026 Aug 28;21(8):e0357231. doi: 10.1371/journal.pone.0357231 (PMC13524278; doi:10.1371/journal.pone.0357231)
Supplement: Supplementary file 6 [file pone.0357231.s003.zip › S3. File. WGCNA analysis/output/WGCNA_softpower.pdf]

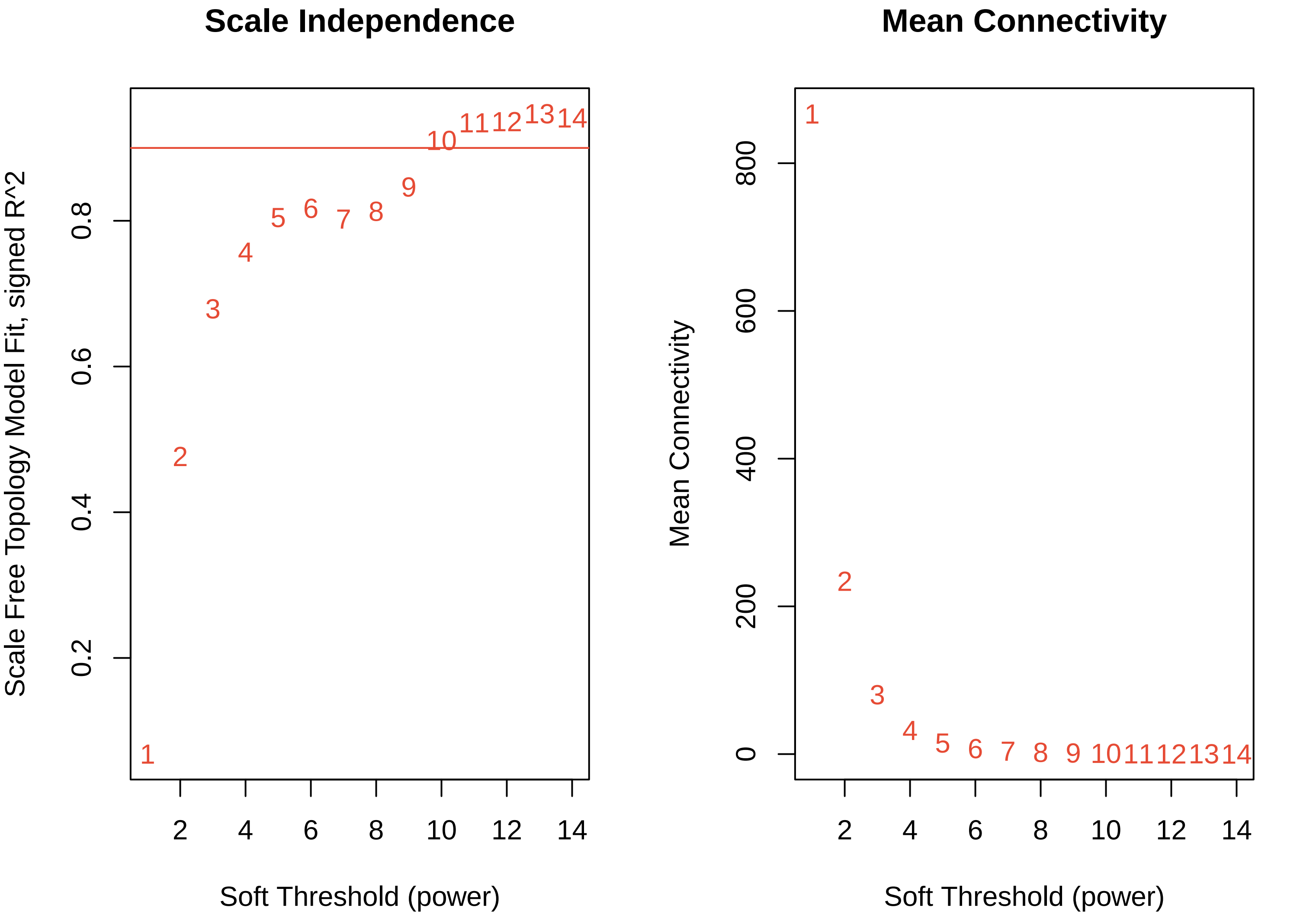
Scale Independence
Mean Connectivity
Scale Free Topology Model Fit, signed R^2
Mean Connectivity
Soft Threshold (power)
Soft Threshold (power)
0.2
0.4
0.6
0.8
0
200
400
600
800
2
4
6
8
10
12
14
