# Supplementary material for: Exploration of acetylation-related biomarkers in osteoarthritis through bioinformatics analysis
Source: PLoS One. 2026 Aug 28;21(8):e0357231. doi: 10.1371/journal.pone.0357231 (PMC13524278; doi:10.1371/journal.pone.0357231)
Supplement: Supplementary file 7 [file pone.0357231.s004.zip › S4. File. Screening ACEDEGs and PPI analysis/output/A_venn.pdf]

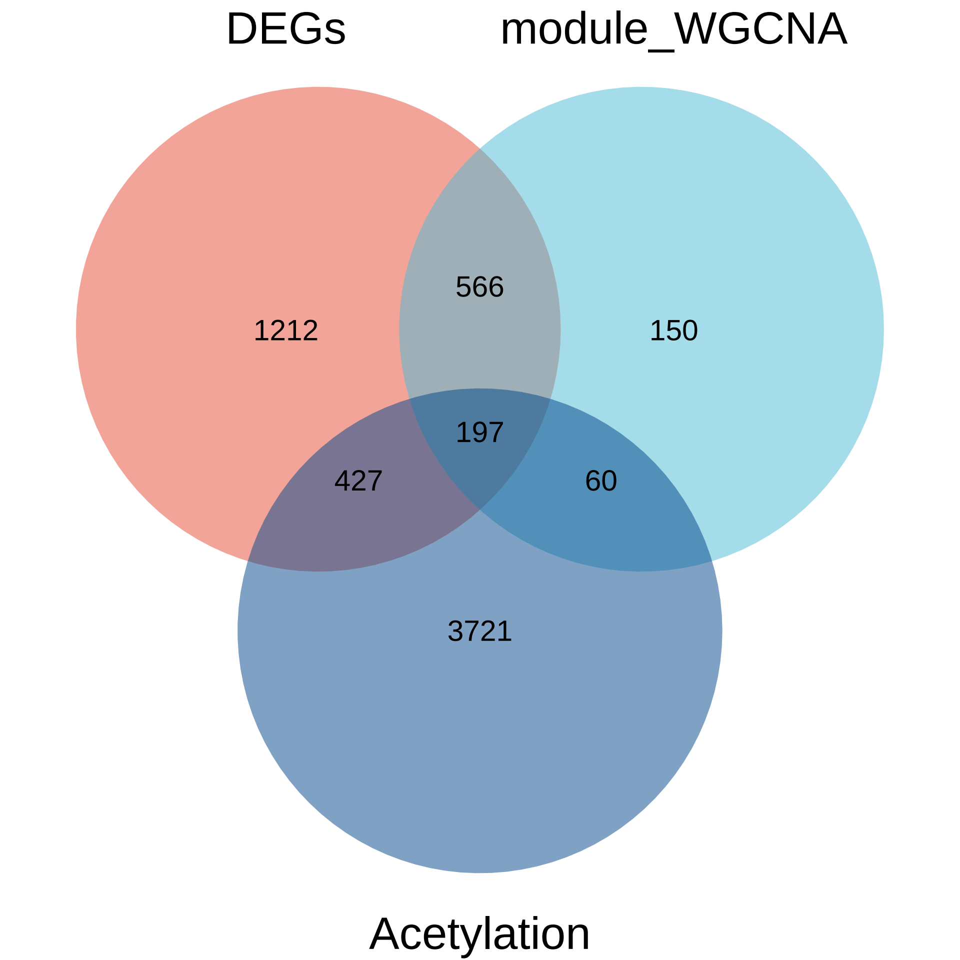
DEGs
module_WGCNA
1212
566
150
197
427
60
3721
Acetylation
