# Supplementary material for: Exploration of acetylation-related biomarkers in osteoarthritis through bioinformatics analysis
Source: PLoS One. 2026 Aug 28;21(8):e0357231. doi: 10.1371/journal.pone.0357231 (PMC13524278; doi:10.1371/journal.pone.0357231)
Supplement: Supplementary file 8 [file pone.0357231.s005.zip › S5. File. 13 ACEDEGs chromosome mapping/ACEDEGs_chromosome_mapping.pdf]

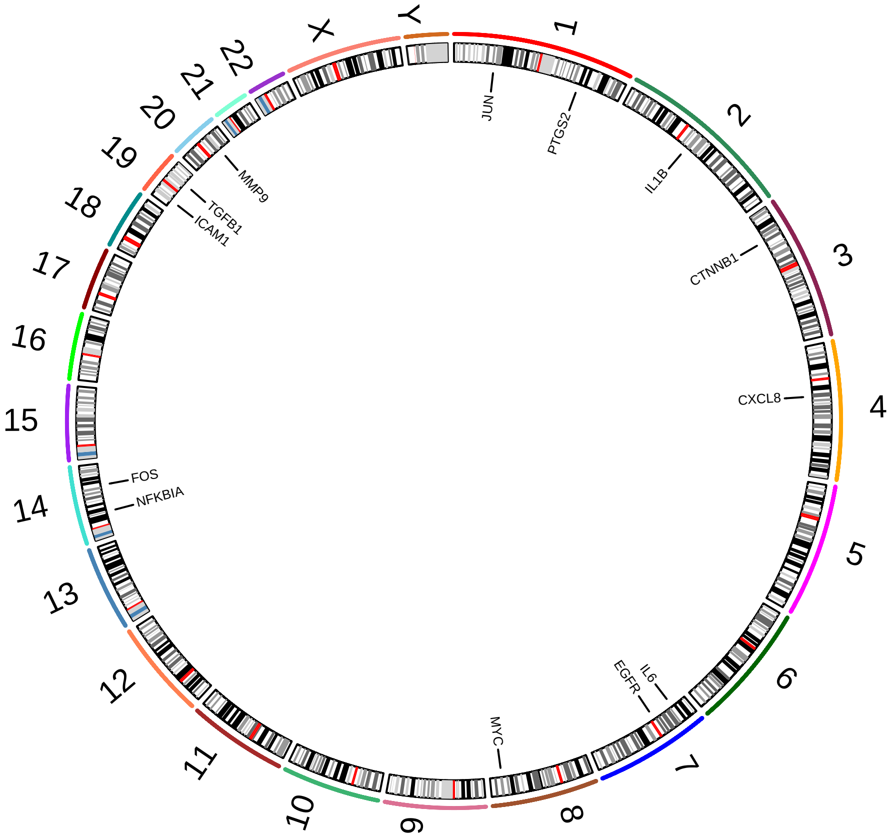
1
2
3
4
5
6
7
8
9
10
11
12
13
14
15
16
17
18
19
20
21
22
X
Y
JUN
PTGS2
IL1B
CTNNB1
CXCL8
IL6
EGFR
MYC
FOS
NFKBIA
ICAM1
TGFB1
MMP9
