# Supplementary material for: Exploration of acetylation-related biomarkers in osteoarthritis through bioinformatics analysis
Source: PLoS One. 2026 Aug 28;21(8):e0357231. doi: 10.1371/journal.pone.0357231 (PMC13524278; doi:10.1371/journal.pone.0357231)
Supplement: Supplementary file 9 [file pone.0357231.s006.zip › S6. File. Machine-learning algorithm screening for hub genes/output/LASSO_coef.pdf]

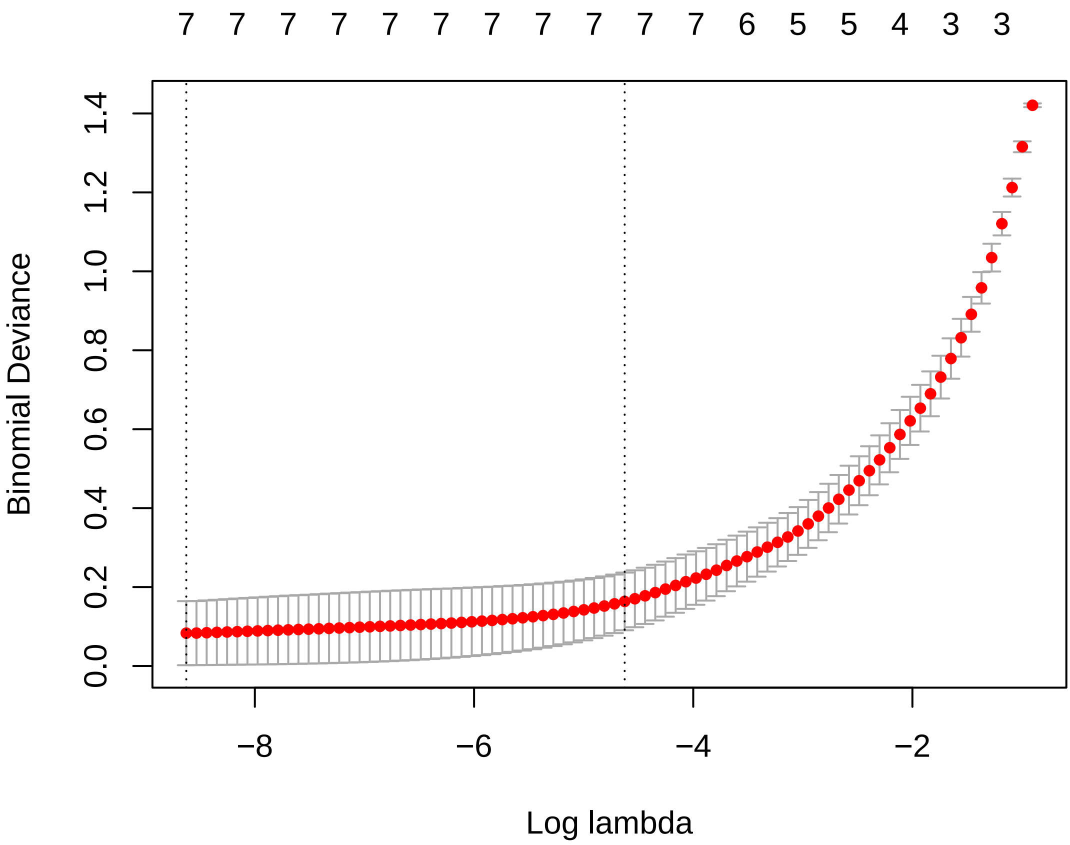

7 7 7 7 7 7 7 7 7 7 7 6 5 5 4 3 3
Binomial Deviance
0.0
0.2
0.4
0.6
0.8
1.0
1.2
1.4
−8
−6
−4
−2
Log lambda
