# Supplementary material for: Exploration of acetylation-related biomarkers in osteoarthritis through bioinformatics analysis
Source: PLoS One. 2026 Aug 28;21(8):e0357231. doi: 10.1371/journal.pone.0357231 (PMC13524278; doi:10.1371/journal.pone.0357231)
Supplement: Supplementary file 9 [file pone.0357231.s006.zip › S6. File. Machine-learning algorithm screening for hub genes/output/RF.pdf]

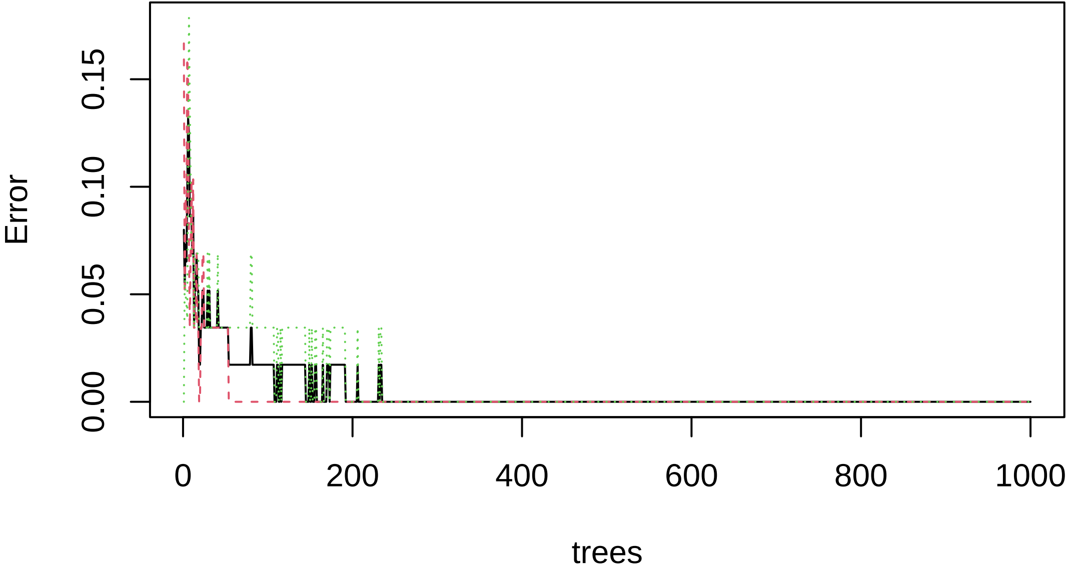
Error
0.00
0.05
0.10
0.15
0
200
400
600
800
1000
trees
