# Supplementary material for: Exploration of acetylation-related biomarkers in osteoarthritis through bioinformatics analysis
Source: PLoS One. 2026 Aug 28;21(8):e0357231. doi: 10.1371/journal.pone.0357231 (PMC13524278; doi:10.1371/journal.pone.0357231)
Supplement: Supplementary file 9 [file pone.0357231.s006.zip › S6. File. Machine-learning algorithm screening for hub genes/output/RF_gene.pdf]

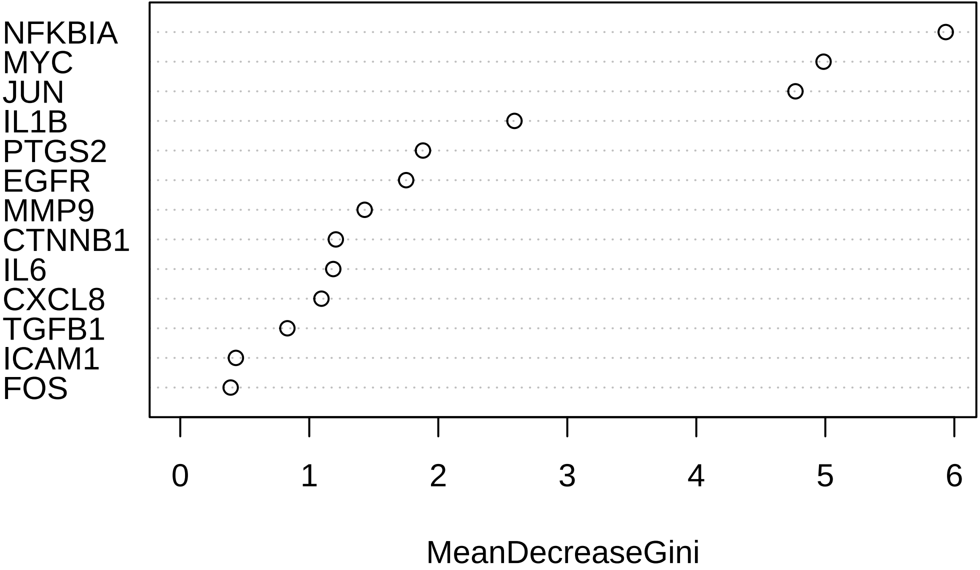
NFKBIA
MYC
JUN
IL1B
PTGS2
EGFR
MMP9
CTNNB1
IL6
CXCL8
TGFB1
ICAM1
FOS
0
1
2
3
4
5
6
MeanDecreaseGini
