# Supplementary material for: Exploration of acetylation-related biomarkers in osteoarthritis through bioinformatics analysis
Source: PLoS One. 2026 Aug 28;21(8):e0357231. doi: 10.1371/journal.pone.0357231 (PMC13524278; doi:10.1371/journal.pone.0357231)
Supplement: Supplementary file 9 [file pone.0357231.s006.zip › S6. File. Machine-learning algorithm screening for hub genes/output/XGB_importance.pdf]

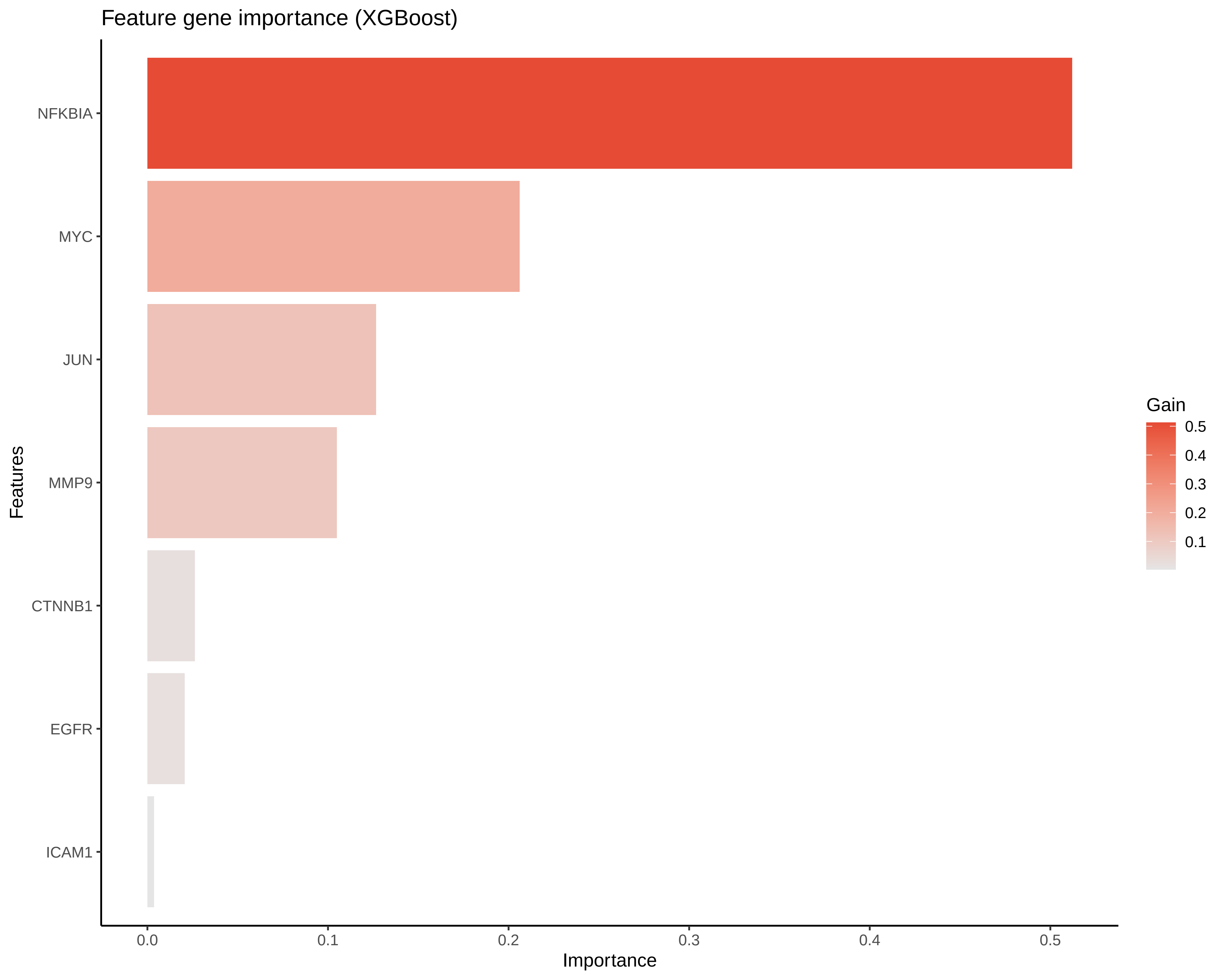
Feature gene importance (XGBoost)
NFKBIA
MYC
JUN
MMP9
CTNNB1
EGFR
ICAM1
Features
0.0
0.1
0.2
0.3
0.4
0.5
Importance
Gain
0.5
0.4
0.3
0.2
0.1
