# Supplementary material for: Exploration of acetylation-related biomarkers in osteoarthritis through bioinformatics analysis
Source: PLoS One. 2026 Aug 28;21(8):e0357231. doi: 10.1371/journal.pone.0357231 (PMC13524278; doi:10.1371/journal.pone.0357231)
Supplement: Supplementary file 9 [file pone.0357231.s006.zip › S6. File. Machine-learning algorithm screening for hub genes/output/XGB_shap_summary.pdf]

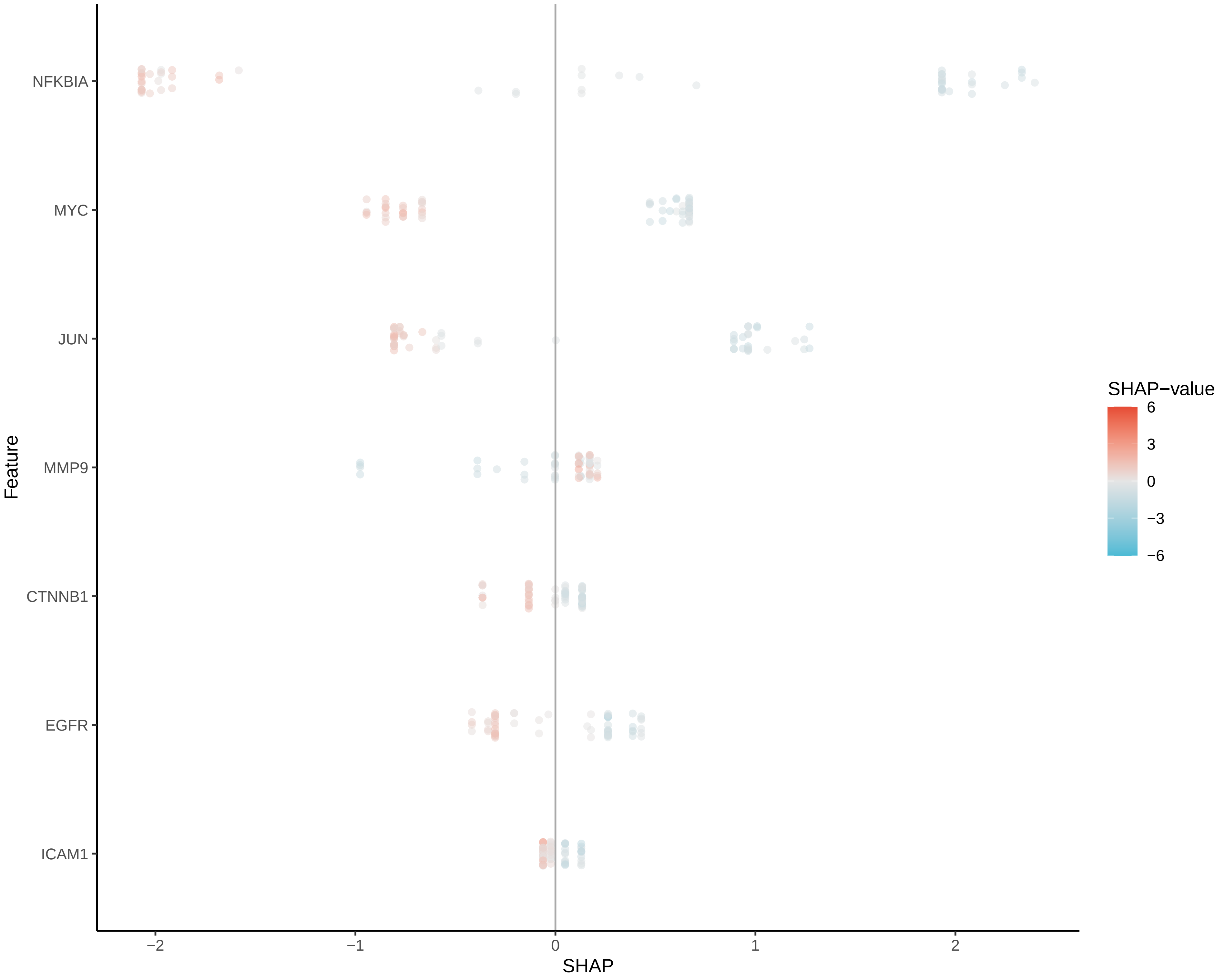
NFKBIA
MYC
JUN
MMP9
CTNNB1
EGFR
ICAM1
Feature
−2
−1
0
1
2
SHAP
SHAP−value
6
3
0
−3
−6
