# Supplementary material for: Exploration of acetylation-related biomarkers in osteoarthritis through bioinformatics analysis
Source: PLoS One. 2026 Aug 28;21(8):e0357231. doi: 10.1371/journal.pone.0357231 (PMC13524278; doi:10.1371/journal.pone.0357231)
Supplement: Supplementary file 10 [file pone.0357231.s007.zip › S7. File. Nomogram construction/5A_diag_nomogram.pdf]

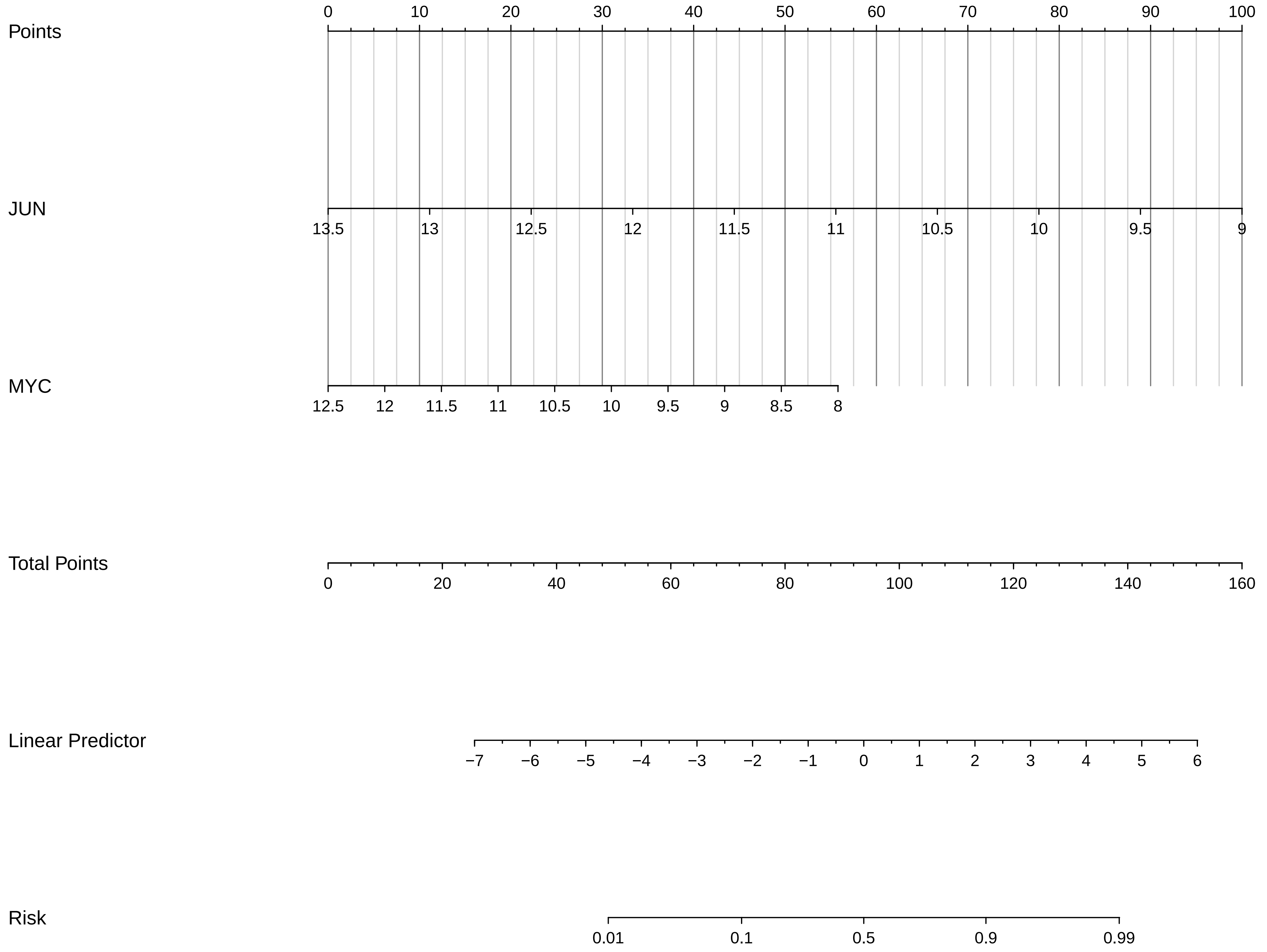

Points
0
10
20
30
40
50
60
70
80
90
100
JUN
13.5
13
12.5
12
11.5
11
10.5
10
9.5
9
MYC
12.5
12
11.5
11
10.5
10
9.5
9
8.5
8
Total Points
0
20
40
60
80
100
120
140
160
Linear Predictor
−7
−6
−5
−4
−3
−2
−1
0
1
2
3
4
5
6
Risk
0.01
0.1
0.5
0.9
0.99
