# Supplementary material for: Exploration of acetylation-related biomarkers in osteoarthritis through bioinformatics analysis
Source: PLoS One. 2026 Aug 28;21(8):e0357231. doi: 10.1371/journal.pone.0357231 (PMC13524278; doi:10.1371/journal.pone.0357231)
Supplement: Supplementary file 10 [file pone.0357231.s007.zip › S7. File. Nomogram construction/5D_CIC.pdf]

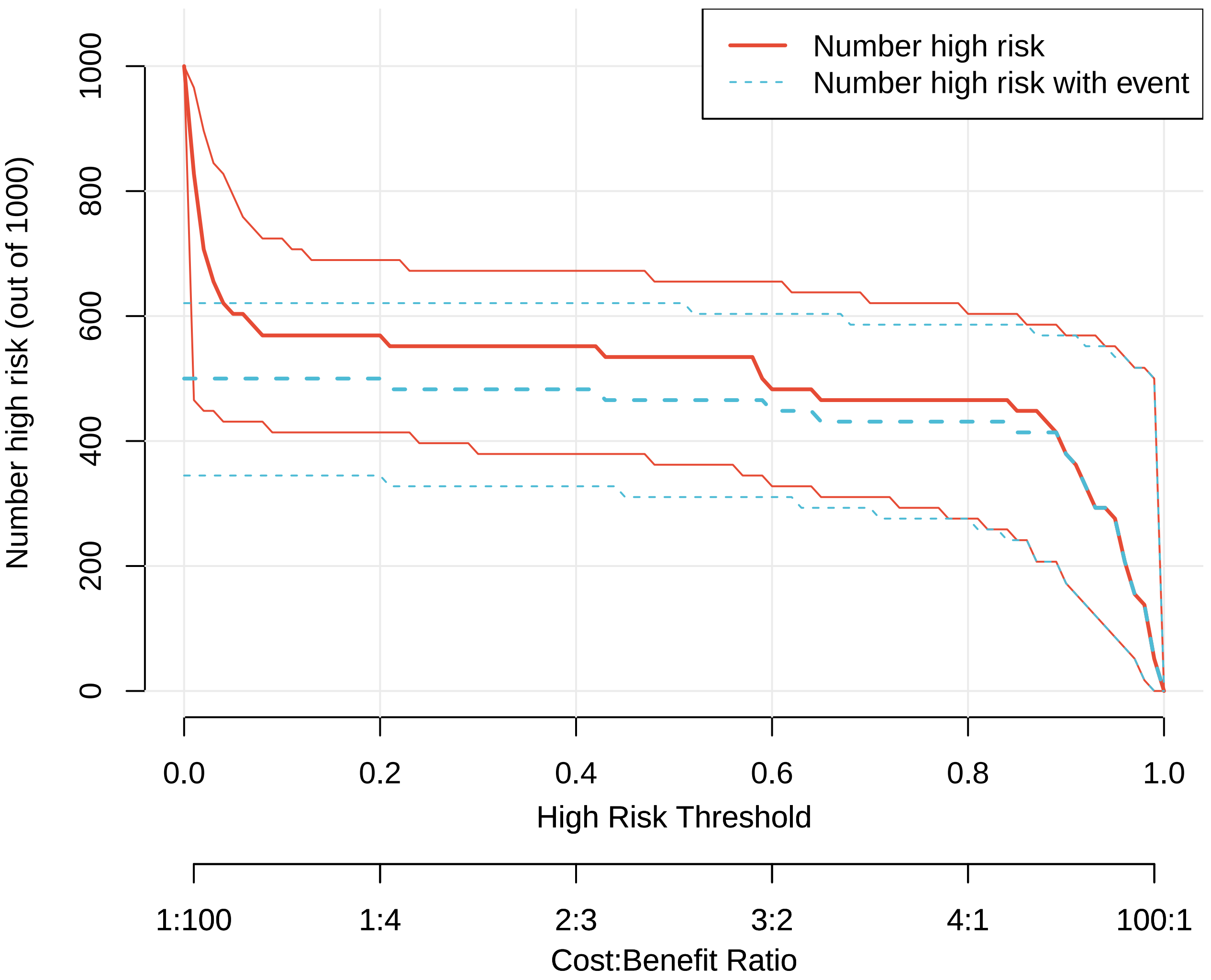
Number high risk
Number high risk with event
Number high risk (out of 1000)
0
200
400
600
800
1000
0.0
0.2
0.4
0.6
0.8
1.0
High Risk Threshold
1:100
1:4
2:3
3:2
4:1
100:1
Cost:Benefit Ratio
