# Supplementary material for: Exploration of acetylation-related biomarkers in osteoarthritis through bioinformatics analysis
Source: PLoS One. 2026 Aug 28;21(8):e0357231. doi: 10.1371/journal.pone.0357231 (PMC13524278; doi:10.1371/journal.pone.0357231)
Supplement: Supplementary file 10 [file pone.0357231.s007.zip › S7. File. Nomogram construction/5E_ROC_nomo.pdf]

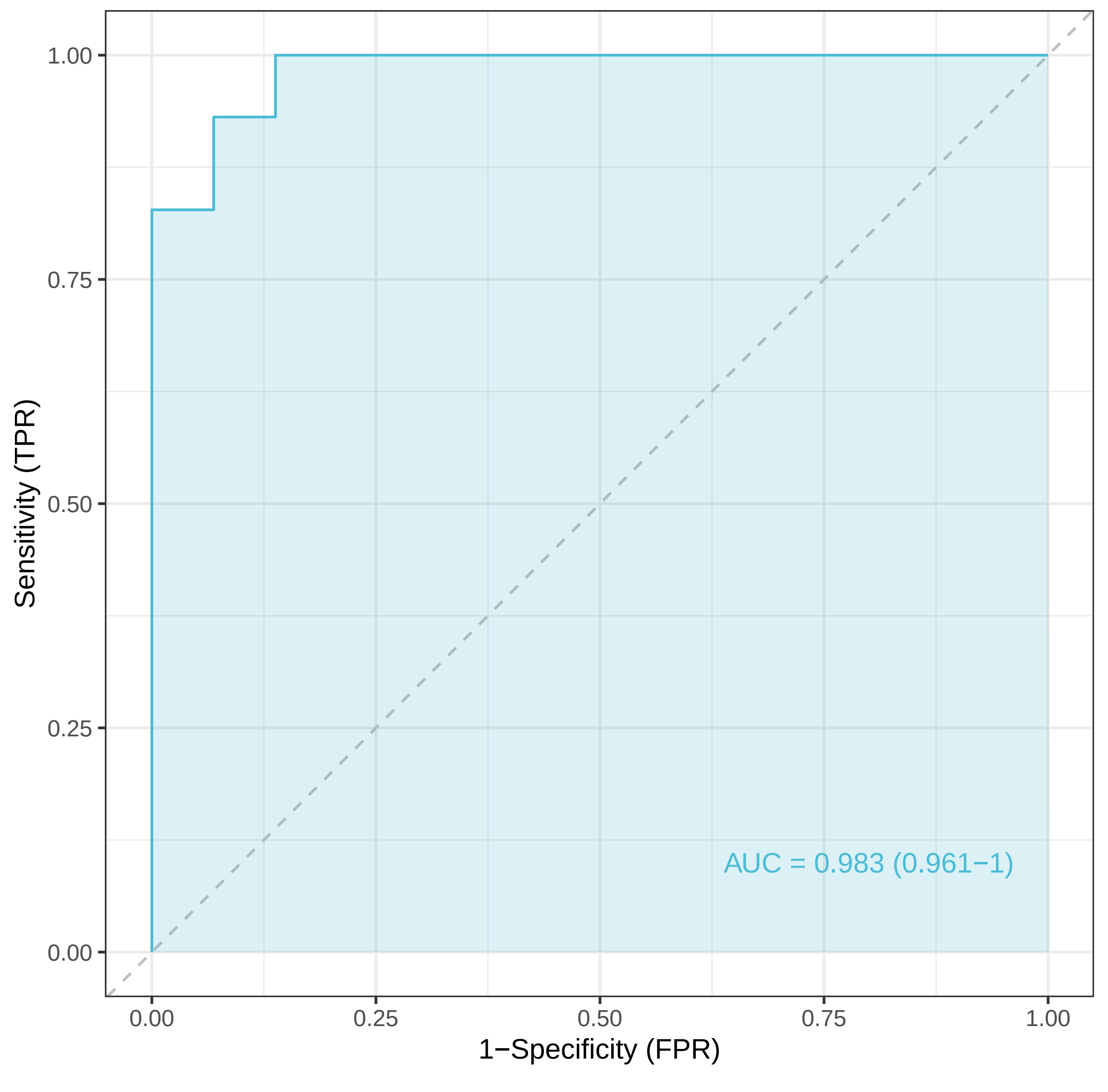
1.00
0.75
0.50
0.25
0.00
Sensitivity (TPR)
0.00
0.25
0.50
0.75
1.00
1−Specificity (FPR)
AUC = 0.983 (0.961−1)
