# Supplementary material for: Exploration of acetylation-related biomarkers in osteoarthritis through bioinformatics analysis
Source: PLoS One. 2026 Aug 28;21(8):e0357231. doi: 10.1371/journal.pone.0357231 (PMC13524278; doi:10.1371/journal.pone.0357231)
Supplement: Supplementary file 10 [file pone.0357231.s007.zip › S7. File. Nomogram construction/Logistic_uni_forestplot.pdf]

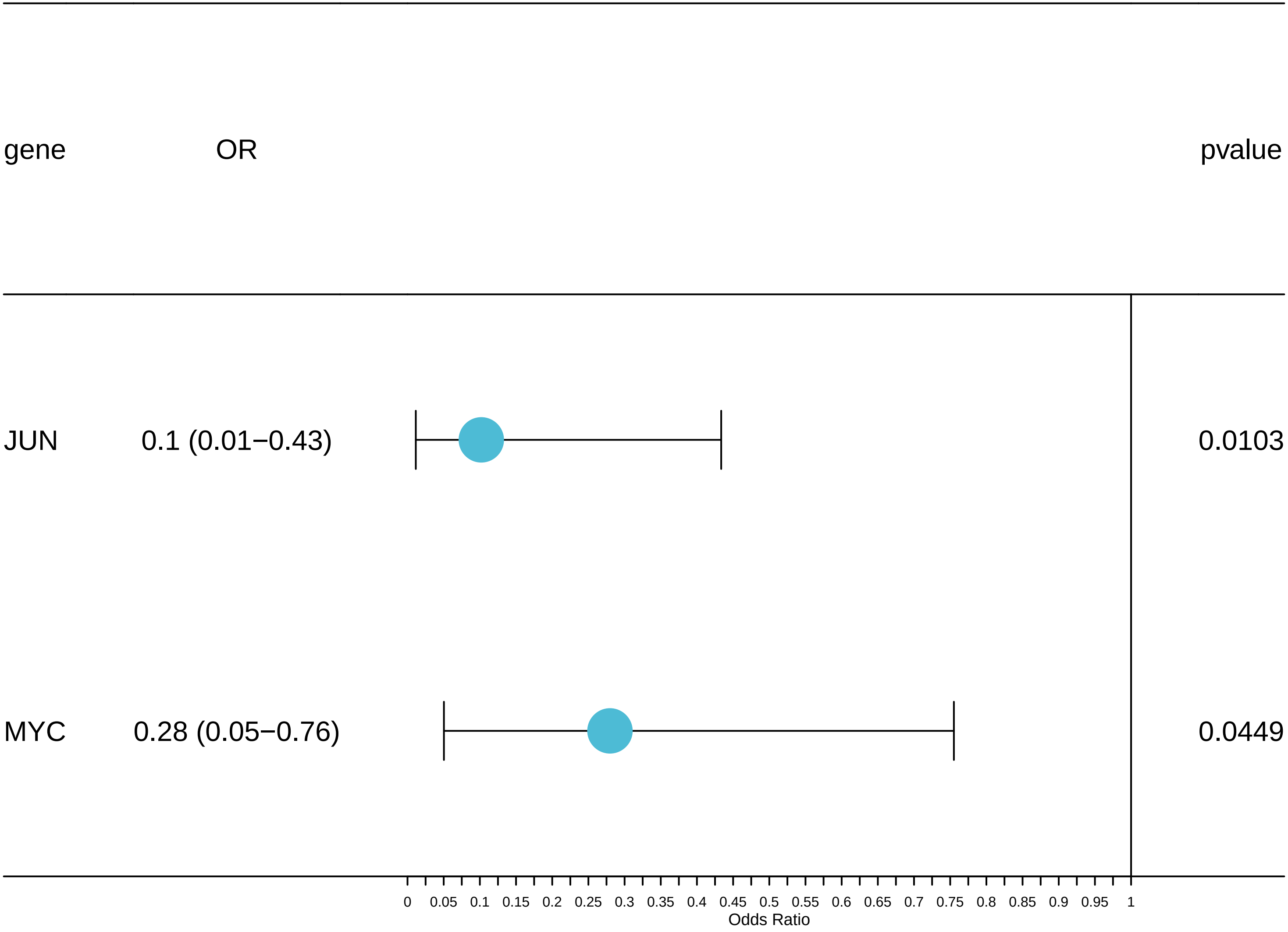
gene
OR
pvalue
JUN
0.1 (0.01−0.43)
0.0103
MYC
0.28 (0.05−0.76)
0.0449
0
0.05
0.1
0.15
0.2
0.25
0.3
0.35
0.4
0.45
0.5
0.55
0.6
0.65
0.7
0.75
0.8
0.85
0.9
0.95
1
Odds Ratio
