# Supplementary material for: Exploration of acetylation-related biomarkers in osteoarthritis through bioinformatics analysis
Source: PLoS One. 2026 Aug 28;21(8):e0357231. doi: 10.1371/journal.pone.0357231 (PMC13524278; doi:10.1371/journal.pone.0357231)
Supplement: Supplementary file 10 [file pone.0357231.s007.zip › S7. File. Nomogram construction/output/5A_diag_nomogram.pdf]

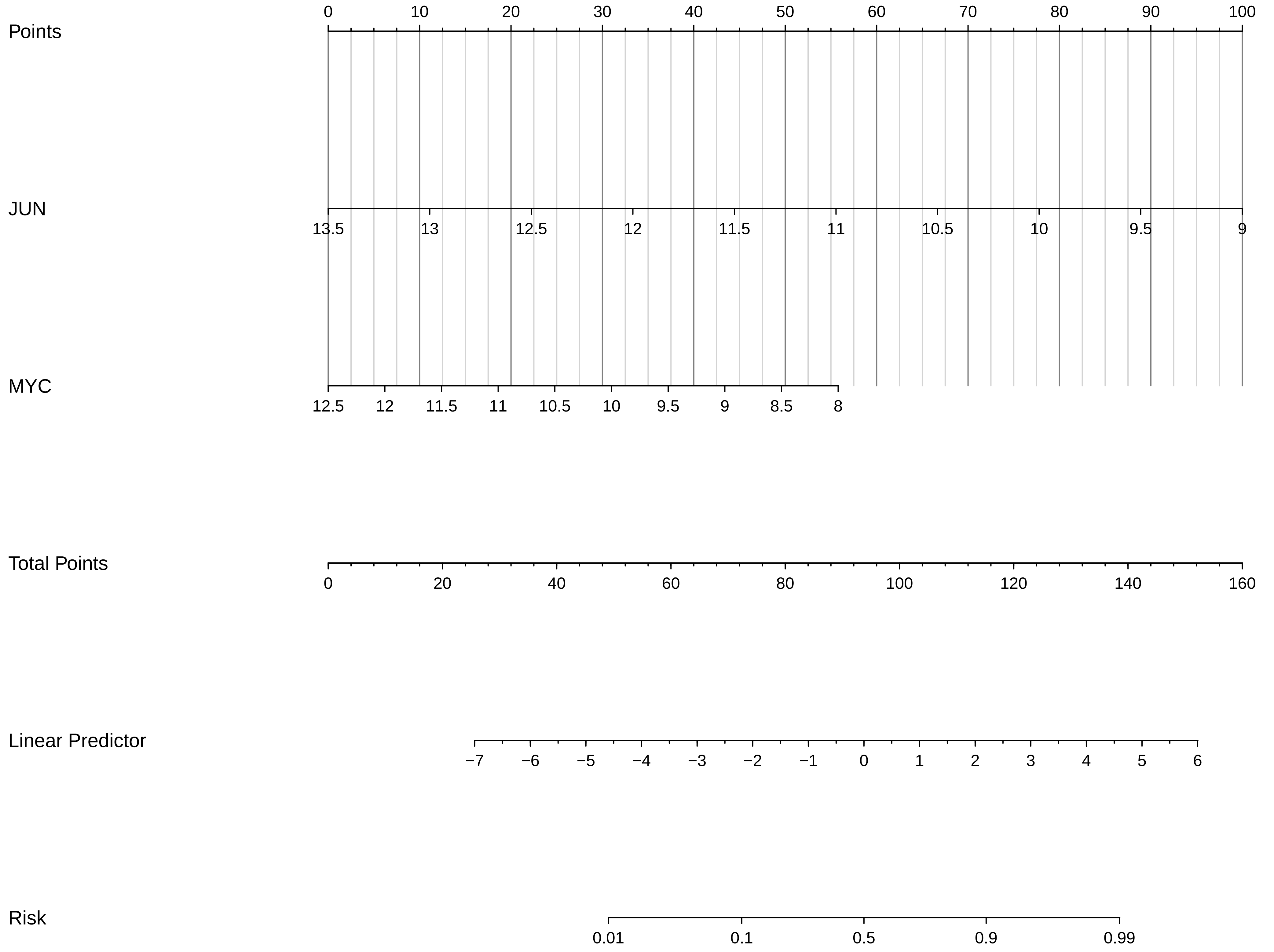

Points
0 10 20 30 40 50 60 70 80 90 100
JUN
13.5 13 12.5 12 11.5 11 10.5 10 9.5 9
MYC
12.5 12 11.5 11 10.5 10 9.5 9 8.5 8
Total Points
0 20 40 60 80 100 120 140 160
Linear Predictor
−7 −6 −5 −4 −3 −2 −1 0 1 2 3 4 5 6
Risk
0.01 0.1 0.5 0.9 0.99
