# Supplementary material for: Exploration of acetylation-related biomarkers in osteoarthritis through bioinformatics analysis
Source: PLoS One. 2026 Aug 28;21(8):e0357231. doi: 10.1371/journal.pone.0357231 (PMC13524278; doi:10.1371/journal.pone.0357231)
Supplement: Supplementary file 12 [file pone.0357231.s009.zip › S9. File. Correlation analysis/corr_heatmap_ssGSEA_cell_cell.pdf]

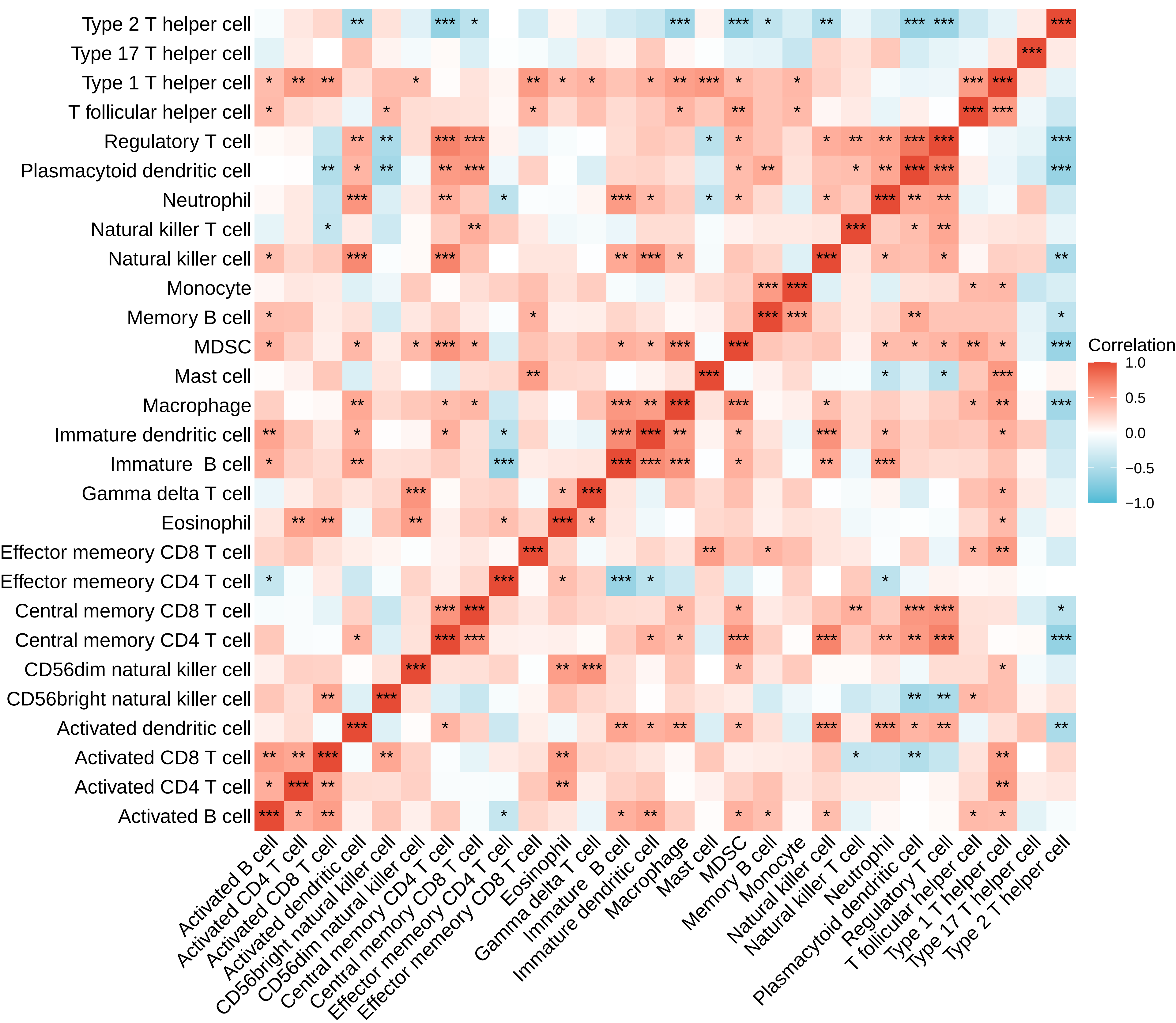
Type 2 T helper cell
Type 17 T helper cell
Type 1 T helper cell
T follicular helper cell
Regulatory T cell
Plasmacytoid dendritic cell
Neutrophil
Natural killer T cell
Natural killer cell
Monocyte
Memory B cell
MDSC
Mast cell
Macrophage
Immature dendritic cell
Immature B cell
Gamma delta T cell
Eosinophil
Effector memeory CD8 T cell
Effector memeory CD4 T cell
Central memory CD8 T cell
Central memory CD4 T cell
CD56dim natural killer cell
CD56bright natural killer cell
Activated dendritic cell
Activated CD8 T cell
Activated CD4 T cell
Activated B cell
Correlation
1.0
0.5
0.0
−0.5
−1.0
