# Supplementary material for: Exploration of acetylation-related biomarkers in osteoarthritis through bioinformatics analysis
Source: PLoS One. 2026 Aug 28;21(8):e0357231. doi: 10.1371/journal.pone.0357231 (PMC13524278; doi:10.1371/journal.pone.0357231)
Supplement: Supplementary file 12 [file pone.0357231.s009.zip › S9. File. Correlation analysis/output/scatter2_JUN_Activated CD4 T cell.pdf]

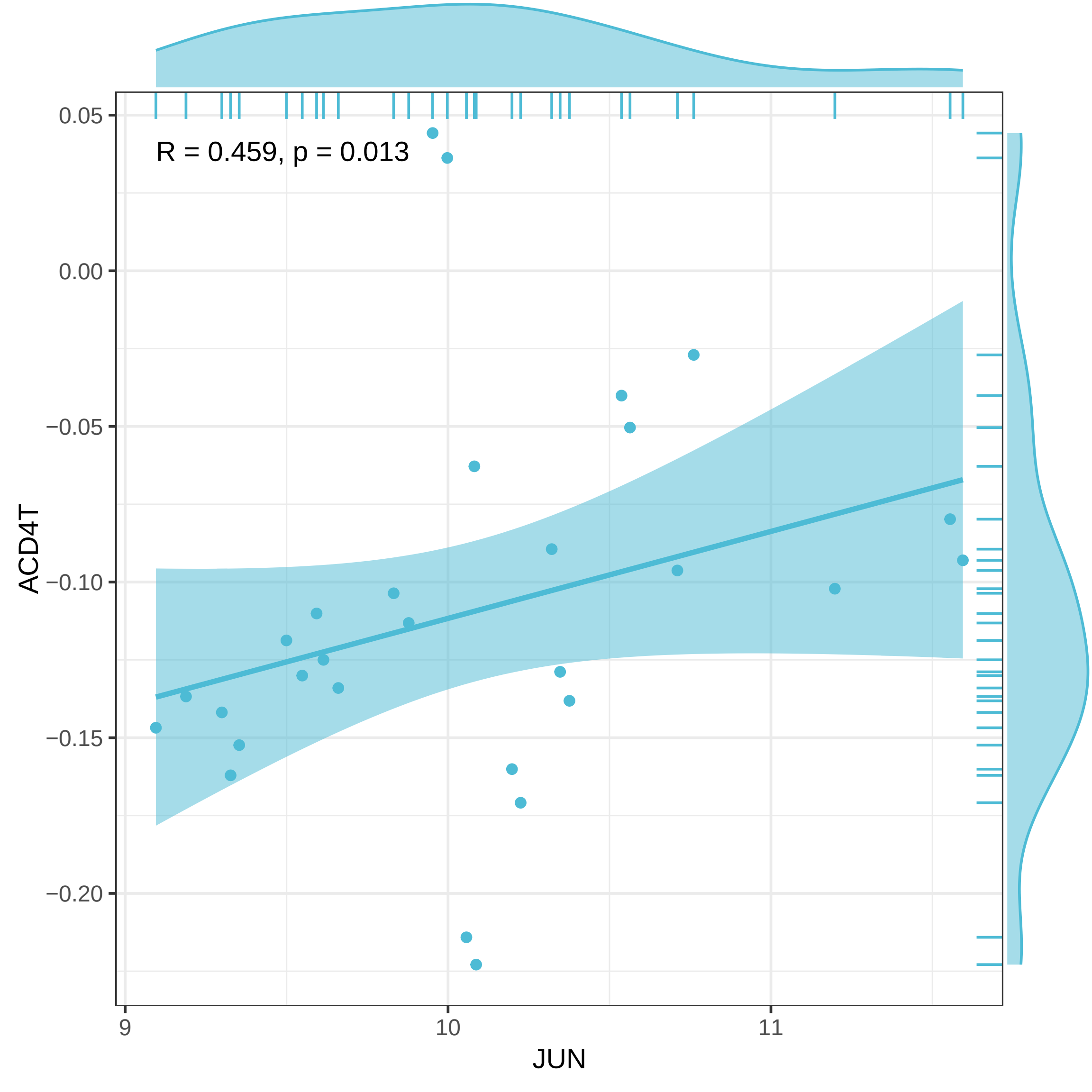

R = 0.459, p = 0.013
0.05
0.00
−0.05
−0.10
−0.15
−0.20
ACD4T
9
10
11
JUN
