# Supplementary material for: Exploration of acetylation-related biomarkers in osteoarthritis through bioinformatics analysis
Source: PLoS One. 2026 Aug 28;21(8):e0357231. doi: 10.1371/journal.pone.0357231 (PMC13524278; doi:10.1371/journal.pone.0357231)
Supplement: Supplementary file 12 [file pone.0357231.s009.zip › S9. File. Correlation analysis/output/scatter2_MYC_Monocyte.pdf]

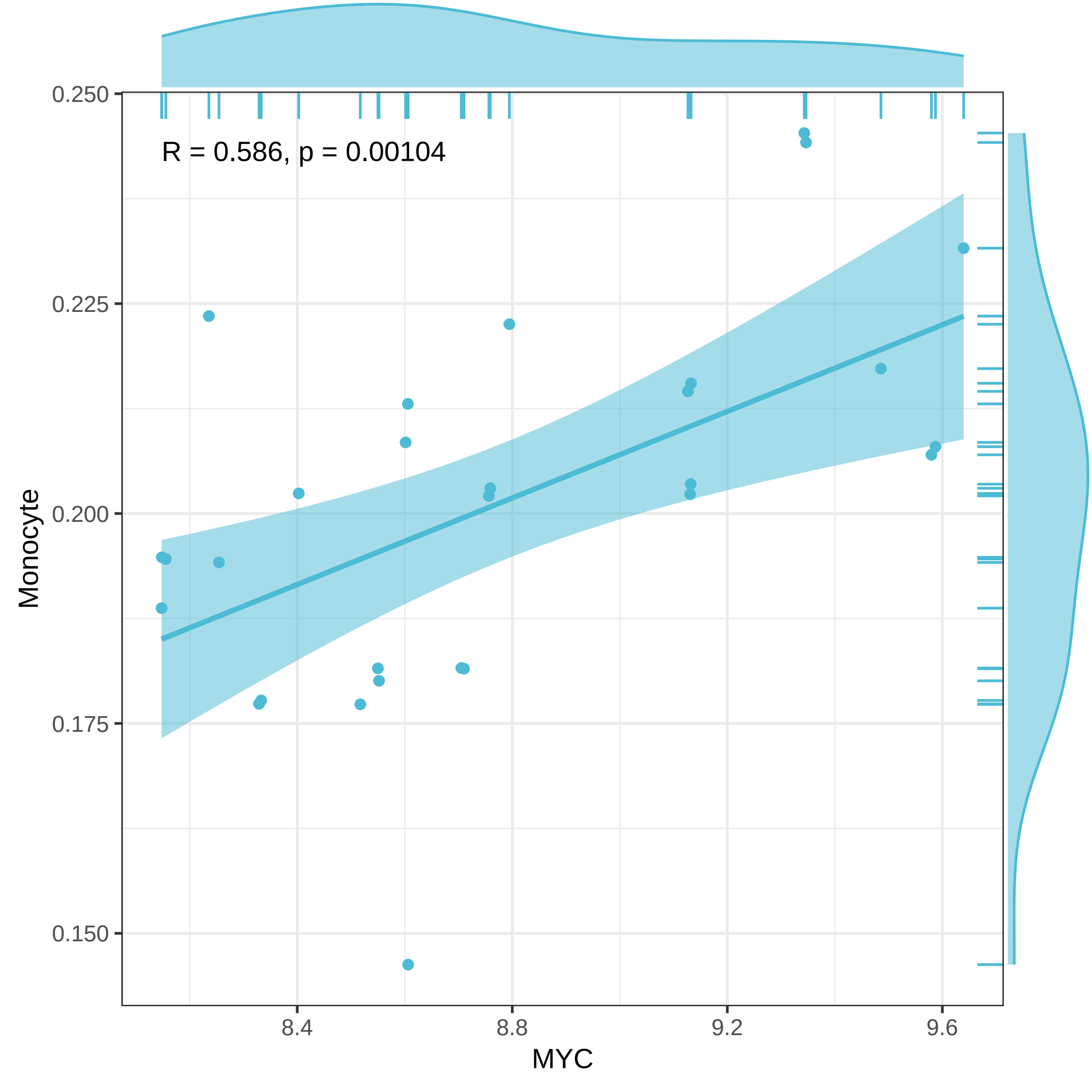
R = 0.586, p = 0.00104
0.250
0.225
0.200
0.175
0.150
Monocyte
8.4
8.8
9.2
9.6
MYC
