# Supplementary material for: Exploration of acetylation-related biomarkers in osteoarthritis through bioinformatics analysis
Source: PLoS One. 2026 Aug 28;21(8):e0357231. doi: 10.1371/journal.pone.0357231 (PMC13524278; doi:10.1371/journal.pone.0357231)
Supplement: Supplementary file 13 [file pone.0357231.s010.zip › S10. File. Gene set enrichment analysis/output/gsea_down_JUN.pdf]

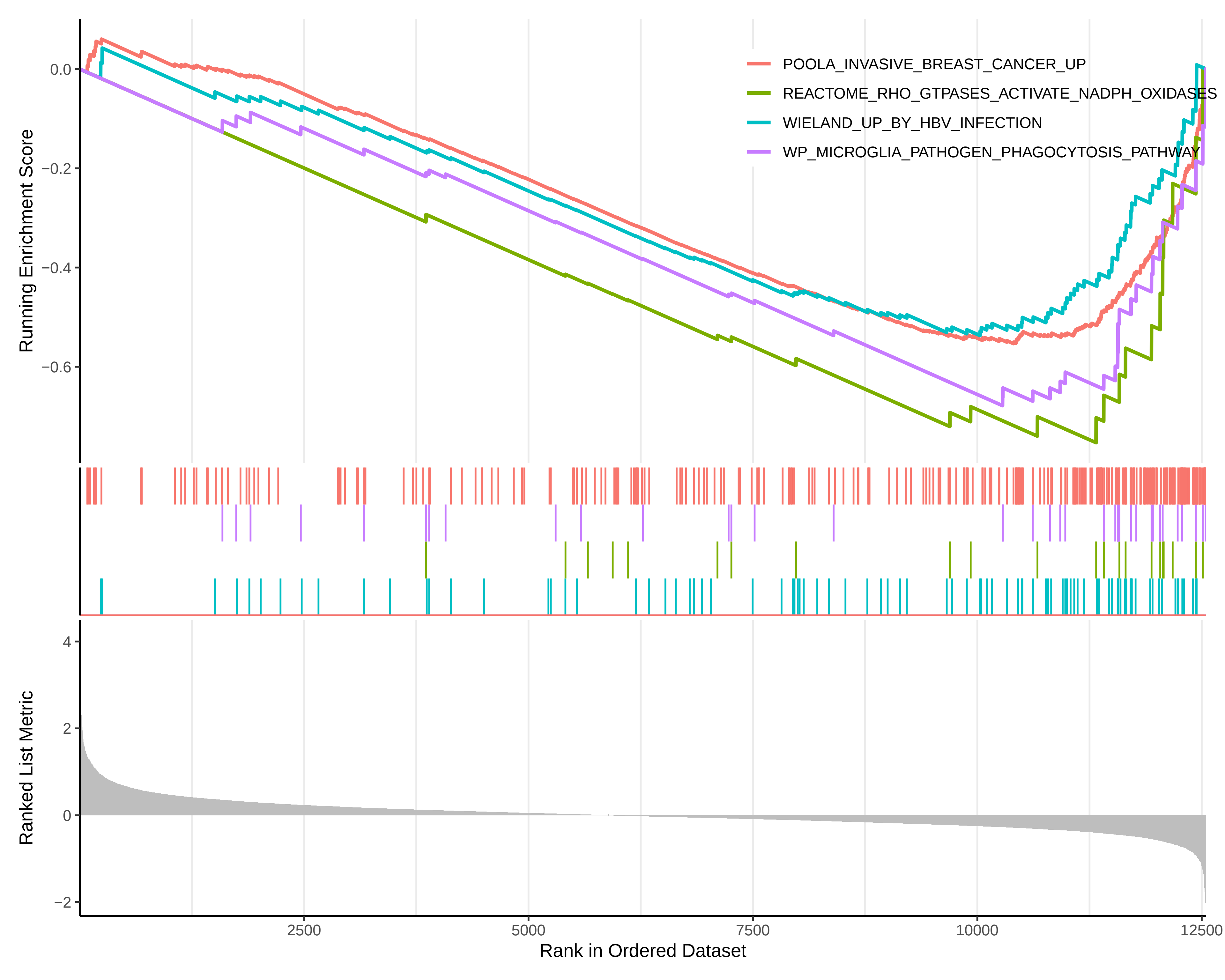
POOLA_INVASIVE_BREAST_CANCER_UP
REACTOME_RHO_GTPASES_ACTIVATE_NADPH_OXIDASES
WIELAND_UP_BY_HBV_INFECTION
WP_MICROGLIA_PATHOGEN_PHAGOCYTOSIS_PATHWAY
Running Enrichment Score
0.0
−0.2
−0.4
−0.6
Ranked List Metric
4
2
0
−2
2500
5000
7500
10000
12500
Rank in Ordered Dataset
