# Supplementary material for: Exploration of acetylation-related biomarkers in osteoarthritis through bioinformatics analysis
Source: PLoS One. 2026 Aug 28;21(8):e0357231. doi: 10.1371/journal.pone.0357231 (PMC13524278; doi:10.1371/journal.pone.0357231)
Supplement: Supplementary file 13 [file pone.0357231.s010.zip › S10. File. Gene set enrichment analysis/output/gsea_down_MYC.pdf]

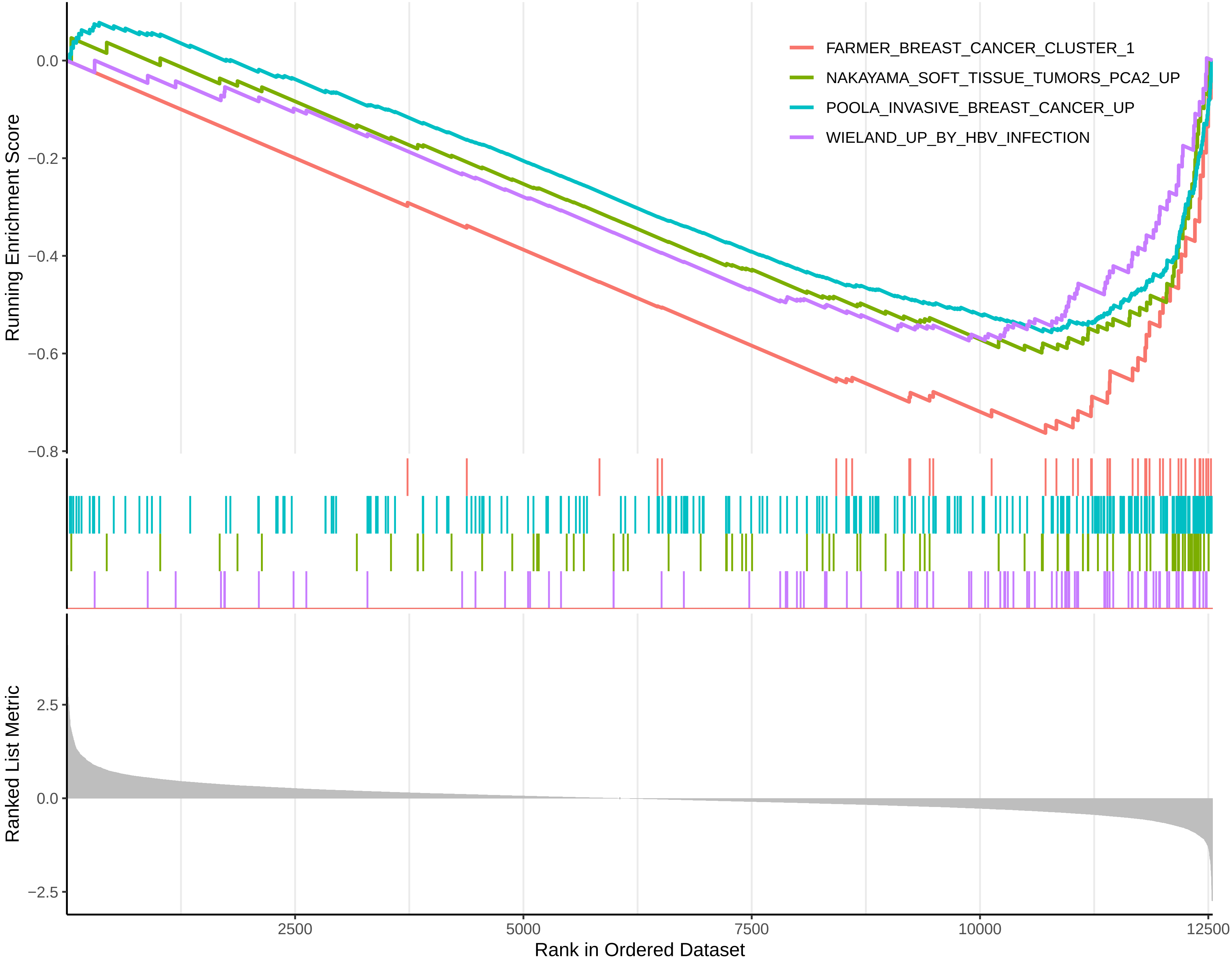

FARMER_BREAST_CANCER_CLUSTER_1
NAKAYAMA_SOFT_TISSUE_TUMORS_PCA2_UP
POOLA_INVASIVE_BREAST_CANCER_UP
WIELAND_UP_BY_HBV_INFECTION
Running Enrichment Score
0.0
−0.2
−0.4
−0.6
−0.8
Ranked List Metric
2.5
0.0
−2.5
2500
5000
7500
10000
12500
Rank in Ordered Dataset
