# Supplementary material for: Exploration of acetylation-related biomarkers in osteoarthritis through bioinformatics analysis
Source: PLoS One. 2026 Aug 28;21(8):e0357231. doi: 10.1371/journal.pone.0357231 (PMC13524278; doi:10.1371/journal.pone.0357231)
Supplement: Supplementary file 13 [file pone.0357231.s010.zip › S10. File. Gene set enrichment analysis/output/gsea_up_JUN.pdf]

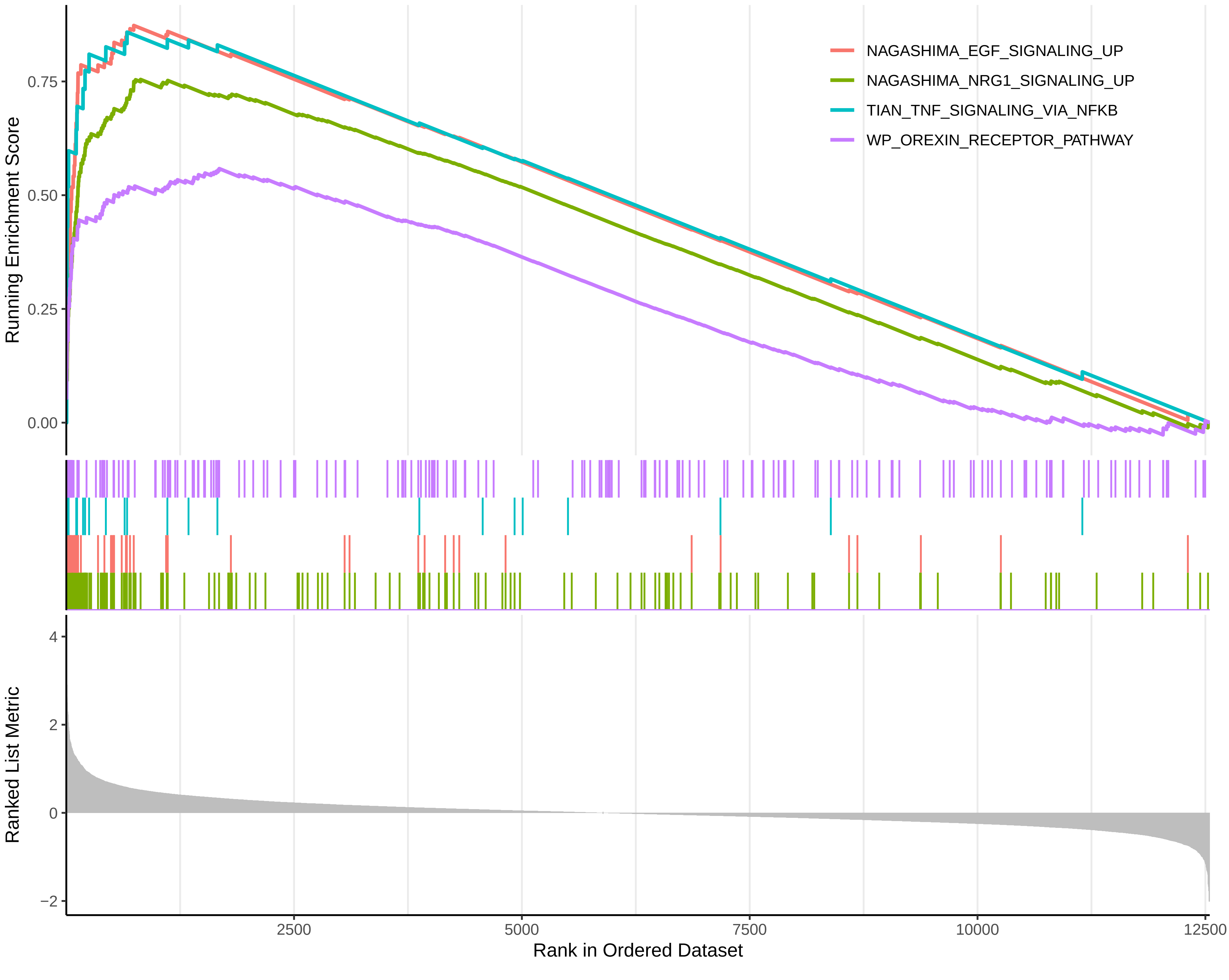

NAGASHIMA_EGF_SIGNALING_UP
NAGASHIMA_NRG1_SIGNALING_UP
TIAN_TNF_SIGNALING_VIA_NFKB
WP_OREXIN_RECEPTOR_PATHWAY
Running Enrichment Score
0.75
0.50
0.25
0.00
Ranked List Metric
4
2
0
−2
2500
5000
7500
10000
12500
Rank in Ordered Dataset
