# Supplementary material for: Exploration of acetylation-related biomarkers in osteoarthritis through bioinformatics analysis
Source: PLoS One. 2026 Aug 28;21(8):e0357231. doi: 10.1371/journal.pone.0357231 (PMC13524278; doi:10.1371/journal.pone.0357231)
Supplement: Supplementary file 15 [file pone.0357231.s013.docx]

Dear Dr. Kurt and the Reviewers,

Thank you very much for the opportunity to revise our manuscript. We sincerely appreciate the time and effort you have dedicated to providing constructive and insightful comments, which have significantly helped us improve the rigor and clarity of our work. We have carefully addressed each point raised below. All revisions in the manuscript are clearly marked using the “Track Changes” feature, and line numbers refer to the revised manuscript file.

Response to Journal Requirements

1. **Please ensure that your manuscript meets PLOS ONE's style requirements, including those for file naming.**

Author response: We have carefully reviewed the provided style templates and adjusted our manuscript accordingly. This includes figure captions, and file names to fully comply with PLOS ONE guidelines.

2. **Please note that PLOS One has specific guidelines on code sharing for submissions in which author-generated code underpins the findings in the manuscript. In these cases, we expect all author-generated code to be made available without restrictions upon publication of the work.**

Author response: Our study is a bioinformatics analysis based entirely on publicly available datasets (GEO,) and open-source R packages. we have provided the full R code for the reproducibility of our analysis pipeline in a supporting Information.

3. **We note that the grant information you provided in the ‘Funding Information’ and ‘Financial Disclosure’ sections do not match.**

Author response: We apologize for this oversight. We have now carefully cross-checked and ensured that the grant numbers listed in both the ‘Funding Information’ and ‘Financial Disclosure’ sections within the submission system are identical and correctly reflect the awards received for this study.

4. **Please note that funding information should not appear in any section or other areas of your manuscript. We will only publish funding information present in the Funding Statement section of the online submission form. Please remove any funding-related text from the manuscript.**

Author response: We have thoroughly reviewed the manuscript and removed any funding-related statements from the main text.

5. **PLOS ONE now requires that authors provide the original uncropped and unadjusted images underlying all blot or gel results reported in a submission’s figures or Supporting Information files. This policy and the journal’s other requirements for blot/gel reporting and figure preparation are described in detail at https://journals.plos.org/plosone/s/figures#loc-blot-and-gel-reporting-requirements and https://journals.plos.org/plosone/s/figures#loc-preparing-figures-from-image-files. When you submit your revised manuscript, please ensure that your figures adhere fully to these guidelines and provide the original underlying images for all blot or gel data reported in your submission.**

Author response: We have carefully reviewed the journal’s guidelines on blot and gel reporting and figure preparation. In the revised manuscript, we have included all original, uncropped, and unadjusted blot/gel images as part of the Supporting information (Supplementary S17 File).

6. **Your ethics statement should only appear in the Methods section of your manuscript. If your ethics statement is written in any section besides the Methods, please delete it from any other section.**

Author response: We have reviewed the manuscript and confirm that the ethics statement appears only in the Methods section (lines154-156). Any mention of ethics in other sections has been removed. The statement now complies with PLOS ONE’s policy.

7. **Please include captions for your Supporting Information files at the end of your manuscript, and update any in-text citations to match accordingly.**

Author response:Thank you for this important suggestion. We have now added a dedicated “Supporting Information” section at the end of the manuscript (after the main text and before the references), which includes the full captions for all supplementary files. In addition, we have carefully reviewed and updated all in-text citations to ensure they match the corresponding file labels and captions. These changes are marked in the revised manuscript with track changes.

8. **If the reviewer comments include a recommendation to cite specific previously published works, please review and evaluate these publications to determine whether they are relevant and should be cited. There is no requirement to cite these works unless the editor has indicated otherwise.**

Author response: We have followed the reviewers' suggestions and carefully reviewed all the references cited of the manuscript. After a comprehensive assessment, we have replaced some references that were not directly related to osteoarthritis with more appropriate and closely related studies.

**Response to Reviewer #1**

**Comment 1: Overly lenient DEG screening criteria: The authors used very loose thresholds (|log2FC| > 0 and p < 0.05), resulting in over 5,300 DEGs. This is unusually permissive and may introduce substantial noise.**

Author response: We fully agree that the original threshold was too permissive. In the revised manuscript, we have adopted a more stringent and widely accepted criterion: |log2FC| > 0.5 and p-value < 0.05 (lines 96-97). This reduced the number of DEGs from >5,300 to 2,402, significantly reducing background noise while retaining biologically meaningful signals.

**Comment 2: Language and presentation: The manuscript contains numerous grammatical errors, awkward phrasing, and repetitive sentences (especially in the Discussion). Professional English language editing is strongly recommended before resubmission.**

Author response: We appreciate the reviewer’s feedback. The manuscript has been thoroughly revised for language quality. We engaged a professional English editing service to correct grammatical errors, improve phrasing, and eliminate repetitive content, particularly in the Discussion section. The revised manuscript now reads more clearly and concisely.

**Concern #3: Machine Learning Approach: Only LASSO and Random Forest were used. Adding at least one more algorithm (e.g., SVM-RFE) and showing consensus would improve robustness.**

Author response: We agree with the reviewer’s suggestion. We added eXtreme Gradient Boosting (XGBoost) as a third machine learning algorithm. The consensus across LASSO, Random Forest, and XGBoost identified the same key acetylation-related biomarkers (JUN, MYC, and NFKBIA). This strengthened the robustness of our findings, and the updated methodology and results are included in the revised manuscript.

**Concern #4: Figures and Tables: Figure legends and in-text descriptions need improvement for clarity and completeness.**

Author response: We have revised figure legends and in-text descriptions to improve clarity and completeness. At the same time, the referenced content in the text has been updated to better guide the readers.

**Concern #5: References: A few citations appear loosely related or irrelevant (e.g., schizophrenia or lung adenocarcinoma papers). Please verify relevance and accuracy.**

Author response: We carefully reviewed all references and removed those that were not directly relevant to our study, including the schizophrenia and lung adenocarcinoma citations. We replaced them with more appropriate references related to osteoarthritis, and bioinformatics. The reference list has been updated and verified for accuracy.

**#2 reviewer**

**Concern #1: Line 22: Please include the completed version of the non-standard acronyms in all first instances.**

Author response: We have ensured that all non-standard acronyms are defined at their first appearance in both the Abstract and the main text (e.g., “(WGCNA)” is now written as “weighted gene co‑expression network analysis (WGCNA)” in the first instance (line 22).

**Concern #2: Line 65: Since JUN and MYC were chosen because of their relevance in analyses, please remove them from the introduction.**

Author response: We removed the specific mention of JUN and MYC from the introduction.

**Concern #3: Line 130-131: This is a result item and should be removed from the methodology section: “Calibration curves indicated a strong alignment between the predicted risks of OA progression and the actual observations.”**

Author response: We removed this sentence from the Methods section.

**Concern #4: Line 263: The computation shows patterns of immune infiltration that are consistent with upstream analyses and should be phrased that way.**

Author response: We rephrased this sentence to state that the immune infiltration patterns were consistent with upstream analyses. The revised text reads: “The computation shows patterns of immune infiltration that are consistent with upstream analyses.” (lines 261-262).

**Concern #5: Line 283-296: There’s no need to rehash the whole introduction on the discussion, please revise to include only the relevant discussion points.**

Author response: We revised the discussion section to remove redundant introductory content. The discussion now focuses only on interpreting our results, comparing them with previous studies, and discussing their implications, without repeating background material.

**Concern #6: Line 310: Please compare these DEGs with that of previous studies.**

Author response: We added a comparison of our identified DEGs with those reported in prior OA studies. This is included in the revised discussion (lines 300-303).

**Concern #7: Line 312: It’s an inflammatory pattern, not an observed increased inflammation. Please write it that way. Please also discuss the nuances of having a possible inflammatory response mediated by plasma cells, M0 macrophages, and resting mast cells. Compare it to previous studies.**

Author response: We rephrased this to describe the results as an “inflammatory pattern” rather than “increased inflammation (lines 306-309).” We added a detailed discussion of the potential roles of plasma cells, M0 macrophages, and resting mast cells in OA immune infiltration, and compared these findings with previous studies (lines 315-319).

**Concern #8: Line 323: the validation of the nomogram only shows a clinical possibility rather than an applicability.**

Author response: We revised the text to clarify that the nomogram validation demonstrates a clinical possibility rather than direct applicability. The sentence now reads: “The validation of the predictive nomogram model incorporating JUN and MYC, with an AUC exceeding 0.7, suggests its potential clinical possibility for early OA diagnosis and risk stratification.” (lines 326-328).

**Concern #9: Line 335: The interconnected pathways could mean a lot of different things, and it’s a big jump to suggest that therapies can be repurposed.**

Author response: We toned down this statement. Instead of suggesting that therapies can be repurposed, we now state: “The retention of cross-disease pathways suggests that existing therapies targeting these pathways may be worth investigating for repurposing in OA treatment, potentially accelerating the use of research findings into clinical practice.” (lines 337-339).

**Concern #10: Line 339: Please elaborate in detail about the discrepancy between mRNA and protein levels.**

Author response: We expanded this discussion to elaborate on the discrepancy between mRNA and protein levels, citing translational efficiency, protein degradation, and post-translational modifications as potential reasons. (lines 342-344).

We believe these comprehensive revisions fully address all the concerns raised. We hope the manuscript is now suitable for publication in PLOS ONE.

Sincerely,

Jun Yao, PhD

Bone and Joint Surgery, The First Affiliated Hospital of Guangxi Medical University, Nanning, Guangxi, China

E-mail:yaojun800524@126.com,

2026-7-8
