# Supplementary material for: Exploration of acetylation-related biomarkers in osteoarthritis through bioinformatics analysis
Source: PLoS One. 2026 Aug 28;21(8):e0357231. doi: 10.1371/journal.pone.0357231 (PMC13524278; doi:10.1371/journal.pone.0357231)
Supplement: Peer review history

# Peer review: Decision Letter 0

**DOI:** 10.1371/journal.pone.0357231.r001

**Authors:** Zeyneb Kurt

8 Jun 2026

Dear Dr. Yao,

Thank you for submitting your manuscript to PLOS ONE. After careful consideration, we feel that it has merit but does not fully meet PLOS ONE’s publication criteria as it currently stands. Therefore, we invite you to submit a revised version of the manuscript that addresses the points raised during the review process.

Please submit your revised manuscript by Jul 23 2026 11:59PM. If you will need more time than this to complete your revisions, please reply to this message or contact the journal office at plosone@plos.org. When you're ready to submit your revision, log on to https://www.editorialmanager.com/pone/ and select the 'Submissions Needing Revision' folder to locate your manuscript file.

- A letter that responds to each point raised by the academic editor and reviewer(s). You should upload this letter as a separate file labeled 'Response to Reviewers'.
- A marked-up copy of your manuscript that highlights changes made to the original version. You should upload this as a separate file labeled 'Revised Manuscript with Track Changes'.
- An unmarked version of your revised paper without tracked changes. You should upload this as a separate file labeled 'Manuscript'.

If you would like to make changes to your financial disclosure, please include your updated statement in your cover letter. Guidelines for resubmitting your figure files are available below the reviewer comments at the end of this letter.

If applicable, we recommend that you deposit your laboratory protocols in protocols.io to enhance the reproducibility of your results. Protocols.io assigns your protocol its own identifier (DOI) so that it can be cited independently in the future. For instructions see: https://journals.plos.org/plosone/s/submission-guidelines#loc-laboratory-protocols. Additionally, PLOS ONE offers an option for publishing peer-reviewed Lab Protocol articles, which describe protocols hosted on protocols.io. Read more information on sharing protocols at https://plos.org/protocols?utm_medium=editorial-email&utm_source=authorletters&utm_campaign=protocols.

As the corresponding author, your ORCID iD is verified in the submission system and will appear in the published article. PLOS supports the use of ORCID, and we encourage all coauthors to register for an ORCID iD and use it as well. Please encourage your coauthors to verify their ORCID iD within the submission system before final acceptance, as unverified ORCID iDs will not appear in the published article. *Only*  the individual author can complete the verification step; PLOS staff *cannot*  verify ORCID iDs on behalf of authors.

We look forward to receiving your revised manuscript.

Kind regards,

Zeyneb Kurt, PhD

Academic Editor

PLOS One

**Journal Requirements:**

When submitting your revision, we need you to address these additional requirements.

1. Please ensure that your manuscript meets PLOS ONE's style requirements, including those for file naming. The PLOS ONE style templates can be found at

https://journals.plos.org/plosone/s/file?id=wjVg/PLOSOne_formatting_sample_main_body.pdf and

https://journals.plos.org/plosone/s/file?id=ba62/PLOSOne_formatting_sample_title_authors_affiliations.pdf

2. Please note that PLOS One has specific guidelines on code sharing for submissions in which author-generated code underpins the findings in the manuscript. In these cases, we expect all author-generated code to be made available without restrictions upon publication of the work. Please review our guidelines at https://journals.plos.org/plosone/s/materials-and-software-sharing#loc-sharing-code and ensure that your code is shared in a way that follows best practice and facilitates reproducibility and reuse.

3. We note that the grant information you provided in the ‘Funding Information’ and ‘Financial Disclosure’ sections do not match.

When you resubmit, please ensure that you provide the correct grant numbers for the awards you received for your study in the ‘Funding Information’ section.

4. Please note that funding information should not appear in any section or other areas of your manuscript. We will only publish funding information present in the Funding Statement section of the online submission form. Please remove any funding-related text from the manuscript.

5. PLOS ONE now requires that authors provide the original uncropped and unadjusted images underlying all blot or gel results reported in a submission’s figures or Supporting Information files. This policy and the journal’s other requirements for blot/gel reporting and figure preparation are described in detail at https://journals.plos.org/plosone/s/figures#loc-blot-and-gel-reporting-requirements and https://journals.plos.org/plosone/s/figures#loc-preparing-figures-from-image-files. When you submit your revised manuscript, please ensure that your figures adhere fully to these guidelines and provide the original underlying images for all blot or gel data reported in your submission. See the following link for instructions on providing the original image data: https://journals.plos.org/plosone/s/figures#loc-original-images-for-blots-and-gels.

In your cover letter, please note whether your blot/gel image data are in Supporting Information or posted at a public data repository, provide the repository URL if relevant, and provide specific details as to which raw blot/gel images, if any, are not available. Email us at plosone@plos.org if you have any questions.

6. Your ethics statement should only appear in the Methods section of your manuscript. If your ethics statement is written in any section besides the Methods, please delete it from any other section.

7. Please include captions for your Supporting Information files at the end of your manuscript, and update any in-text citations to match accordingly. Please see our Supporting Information guidelines for more information: http://journals.plos.org/plosone/s/supporting-information.

8. If the reviewer comments include a recommendation to cite specific previously published works, please review and evaluate these publications to determine whether they are relevant and should be cited. There is no requirement to cite these works unless the editor has indicated otherwise.

[Note: HTML markup is below. Please do not edit.]

Reviewers' comments:

Reviewer's Responses to Questions

**Comments to the Author**

1. Is the manuscript technically sound, and do the data support the conclusions?

Reviewer #1: Yes

Reviewer #2: Yes

**********

2. Has the statistical analysis been performed appropriately and rigorously?

Reviewer #1: Yes

Reviewer #2: Yes

**********

3. Have the authors made all data underlying the findings in their manuscript fully available?

The PLOS Data policy

Reviewer #1: Yes

Reviewer #2: Yes

**********

4. Is the manuscript presented in an intelligible fashion and written in standard English?

Reviewer #1: No

Reviewer #2: Yes

**********

Reviewer #1: 1. Overly lenient DEG screening criteria: The authors used very loose thresholds (|log2FC| > 0 and p < 0.05), resulting in over 5,300 DEGs. This is unusually permissive and may introduce substantial noise.

2. Language and presentation: The manuscript contains numerous grammatical errors, awkward phrasing, and repetitive sentences (especially in the Discussion). Professional English language editing is strongly recommended before resubmission.

3. Machine Learning Approach: Only LASSO and Random Forest were used. Adding at least one more algorithm (e.g., SVM-RFE) and showing consensus would improve robustness.

4. Figures and Tables: Figure legends and in-text descriptions need improvement for clarity and completeness.

5. References: A few citations appear loosely related or irrelevant (e.g., schizophrenia or lung adenocarcinoma papers). Please verify relevance and accuracy.

Reviewer #2: Line 22: Please include the completed version of the non-standard acronyms in all first instances.

Line 65: Since JUN and MYC were chosen because of their relevance in analyses, please remove them from the introduction.

Line 130-131: This is a result item and should be removed from the methodology section: “Calibration curves indicated a strong alignment between the predicted risks of OA progression and the actual observations.”

Line 263: The computation shows patterns of immune infiltration that are consistent with upstream analyses and should be phrased that way.

Line 283-296: There’s no need to rehash the whole introduction on the discussion, please revise to include only the relevant discussion points

Line 310: Please compare these DEGs with that of previous studies.

Line 312: It’s an inflammatory pattern, not an observed increased inflammation. Please write it that way. Please also discuss the nuances of having a possible inflammatory response mediated by plasma cells, M0 macrophages, and resting mast cells. Compare it to previous studies.

Line 323: the validation of the nomogram only shows a clinical possibility rather than an applicability.

Line 335: The interconnected pathways could mean a lot of different things, and it’s a big jump to suggest that therapies can be repurposed.

Line 339: Please elaborate in detail about the discrepancy between mRNA and protein levels.

**********

what does this mean?). If published, this will include your full peer review and any attached files.

If you choose “no”, your identity will remain anonymous but your review may still be made public.

**Do you want your identity to be public for this peer review?**  For information about this choice, including consent withdrawal, please see our Privacy Policy

Reviewer #1: **Yes:**  Zahra Ziaastani

Reviewer #2: No

**********

[NOTE: If reviewer comments were submitted as an attachment file, they will be attached to this email and accessible via the submission site. Please log into your account, locate the manuscript record, and check for the action link "View Attachments". If this link does not appear, there are no attachment files.]

To ensure your figures meet our technical requirements, please review our figure guidelines: https://journals.plos.org/plosone/s/figures

You may also use PLOS’s free figure tool, NAAS, to help you prepare publication quality figures: https://journals.plos.org/plosone/s/figures#loc-tools-for-figure-preparation.

NAAS will assess whether your figures meet our technical requirements by comparing each figure against our figure specifications.

# Author comment: Author response to Decision Letter 1

**DOI:** 10.1371/journal.pone.0357231.r002

12 Jul 2026

Please refer to the attached file “Response to Reviewers” for our detailed responses to all specific reviewer and editor comments. All revisions are clearly marked in the "Revised Manuscript with Track Changes".

Attachment Submitted filename: Response to Reviewers.docx

# Editor report: Decision Letter 1

**DOI:** 10.1371/journal.pone.0357231.r003

**Authors:** Zeyneb Kurt

23 Jul 2026

Dear Dr. Yao,

Thank you for submitting your manuscript to PLOS ONE. After careful consideration, we feel that it has merit but does not fully meet PLOS ONE’s publication criteria as it currently stands. Therefore, we invite you to submit a revised version of the manuscript that addresses the points raised during the review process.

Please submit your revised manuscript by Sep 05 2026 11:59PM. If you will need more time than this to complete your revisions, please reply to this message or contact the journal office at plosone@plos.org. When you're ready to submit your revision, log on to https://www.editorialmanager.com/pone/ and select the 'Submissions Needing Revision' folder to locate your manuscript file.

- A letter that responds to each point raised by the academic editor and reviewer(s). You should upload this letter as a separate file labeled 'Response to Reviewers'.
- A marked-up copy of your manuscript that highlights changes made to the original version. You should upload this as a separate file labeled 'Revised Manuscript with Track Changes'.
- An unmarked version of your revised paper without tracked changes. You should upload this as a separate file labeled 'Manuscript'.

If you would like to make changes to your financial disclosure, please include your updated statement in your cover letter. Guidelines for resubmitting your figure files are available below the reviewer comments at the end of this letter.

If applicable, we recommend that you deposit your laboratory protocols in protocols.io to enhance the reproducibility of your results. Protocols.io assigns your protocol its own identifier (DOI) so that it can be cited independently in the future. For instructions see: https://journals.plos.org/plosone/s/submission-guidelines#loc-laboratory-protocols. Additionally, PLOS ONE offers an option for publishing peer-reviewed Lab Protocol articles, which describe protocols hosted on protocols.io. Read more information on sharing protocols at https://plos.org/protocols?utm_medium=editorial-email&utm_source=authorletters&utm_campaign=protocols.

As the corresponding author, your ORCID iD is verified in the submission system and will appear in the published article. PLOS supports the use of ORCID, and we encourage all coauthors to register for an ORCID iD and use it as well. Please encourage your coauthors to verify their ORCID iD within the submission system before final acceptance, as unverified ORCID iDs will not appear in the published article. *Only*  the individual author can complete the verification step; PLOS staff *cannot*  verify ORCID iDs on behalf of authors.

We look forward to receiving your revised manuscript.

Kind regards,

Zeyneb Kurt, PhD

Academic Editor

PLOS One

Journal Requirements:

If the reviewer comments include a recommendation to cite specific previously published works, please review and evaluate these publications to determine whether they are relevant and should be cited. There is no requirement to cite these works unless the editor has indicated otherwise.

Please review your reference list to ensure that it is complete and correct. If you have cited papers that have been retracted, please include the rationale for doing so in the manuscript text, or remove these references and replace them with relevant current references. Any changes to the reference list should be mentioned in the rebuttal letter that accompanies your revised manuscript. If you need to cite a retracted article, indicate the article’s retracted status in the References list and also include a citation and full reference for the retraction notice.

Additional Editor Comments:

-There are some repetitive parts/text in Discussion e.g., lines 287-88 & 290-91; Lines 298-99 & 301-04. Please remove any redundancy.

-Reviewer-2's comment 'Please compare these DEGs with that of previous studies' was not fully addressed in my opinion; please discuss some further relevant studies on the shortlisted DEGs.

-R scripts that are provided in separate suppl files could have been provided as a single file (or maybe on a GitHub or zenodo link) where a proper comment section is added for every step e.g. dataset curation, merging, processing, DGE analysis, and so on and please write complementary code comments in English. Though you can keep them as separate files, please provide comments in R scripts in English.

[Note: HTML markup is below. Please do not edit.]

**********

[NOTE: If reviewer comments were submitted as an attachment file, they will be attached to this email and accessible via the submission site. Please log into your account, locate the manuscript record, and check for the action link "View Attachments". If this link does not appear, there are no attachment files.]

To ensure your figures meet our technical requirements, please review our figure guidelines: https://journals.plos.org/plosone/s/figures

You may also use PLOS’s free figure tool, NAAS, to help you prepare publication quality figures: https://journals.plos.org/plosone/s/figures#loc-tools-for-figure-preparation.

NAAS will assess whether your figures meet our technical requirements by comparing each figure against our figure specifications.

# Author comment: Author response to Decision Letter 2

**DOI:** 10.1371/journal.pone.0357231.r004

5 Aug 2026

Response to Journal Requirements

1.If the reviewer comments include a recommendation to cite specific previously published works, please review and evaluate these publications to determine whether they are relevant and should be cited. There is no requirement to cite these works unless the editor has indicated otherwise.

Author response: We have carefully reviewed the reviewer comments and confirm that no specific previously published works were recommended for citation. Nevertheless, we re-evaluated all references cited in our manuscript to ensure their relevance and accuracy.

2. Please review your reference list to ensure that it is complete and correct. If you have cited papers that have been retracted, please include the rationale for doing so in the manuscript text, or remove these references and replace them with relevant current references. Any changes to the reference list should be mentioned in the rebuttal letter that accompanies your revised manuscript. If you need to cite a retracted article, indicate the article’s retracted status in the References list and also include a citation and full reference for the retraction notice.

Author response: We have thoroughly verified the entire reference list. During this verification process, we identified that reference #25 required correction; we have accordingly replaced it with a more appropriate and relevant citation that directly supports the corresponding statement in the text. The change to the reference list are clearly marked in the revised manuscript using track changes.

Additional Editor Comments

Concern 1: There are some repetitive parts/text in Discussion e.g., lines 287-288 & 290-291; Lines 298-299 & 301-04. Please remove any redundancy.

Author response: We thank the Editor for identifying the redundant passages. We have carefully revised the Discussion to remove duplicate statements:

The overlapping text at lines 287–288 and 290–291 has been merged into one concise statement. The revised Discussion now presents each point only once, with improved clarity. These changes are located at lines 287–289 in the revised manuscript.

In the revised manuscript, we have deleted the redundant text at lines 301–304, as it repeated information already presented in lines 296–298.

Concern 2: Please compare these DEGs with that of previous studies' was not fully addressed in my opinion; please discuss some further relevant studies on the shortlisted DEGs.

Author response: Thank you for your constructive comment. We agree that a direct comparison between our identified DEGs and those from previous studies is essential to strengthen the manuscript. Accordingly, we have revised the Discussion section to further discuss relevant studies on the shortlisted DEGs (JUN and MYC).

Specifically, we now cite previous studies demonstrating the involvement of JUN in chondrocyte apoptosis and extracellular matrix degradation via the AP-1 transcription factor complex, and MYC in regulating chondrocyte proliferation and differentiation, with aberrant expression linked to OA progression. We also emphasize that both genes are subject to acetylation modifications, which adds an epigenetic layer to their regulation in OA. Importantly, we note that several bioinformatics studies have also reported downregulation of JUN and MYC in OA tissues, which is consistent with our qRT-PCR and Western blotting results. In addition, we now discuss other acetylation-related DEGs identified in our study (FOS, EGR1, and ATF3) that have been associated with OA pathology, reinforcing the reliability of our analytical pipeline.

These revisions are marked in the revised manuscript (lines 300–315). We hope this adequately addresses your concern.

Concern #3: R scripts that are provided in separate suppl files could have been provided as a single file (or maybe on a GitHub or zenodo link) where a proper comment section is added for every step e.g. dataset curation, merging, processing, DGE analysis, and so on and please write complementary code comments in English. Though you can keep them as separate files, please provide comments in R scripts in English.

Author response: Thank you for your valuable suggestion regarding the organization of our R scripts. We fully understand the importance of clear, reproducible code. However, given that our bioinformatics analysis consists of several relatively independent analytical modules , we have chosen to keep the scripts organized in separate folders corresponding to each analytical step. This modular structure allows readers to navigate and execute specific analyses more easily, without having to run the entire pipeline as a single block of code.

In accordance with the journal’s guidelines, we have ensured that all R scripts are now accompanied by detailed explanatory comments written in English.

Thank you again for this helpful comment. We hope the revised scripts are now acceptable.

Concern #4: To ensure your figures meet our technical requirements, please review our figure guidelines: https://journals.plos.org/plosone/s/figures

You may also use PLOS’s free figure tool, NAAS, to help you prepare publication quality figures: https://journals.plos.org/plosone/s/figures#loc-tools-for-figure-preparation.

NAAS will assess whether your figures meet our technical requirements by comparing each figure against our figure specifications.

Author response: We have carefully reviewed the PLOS ONE figure guidelines at the provided link. We have checked and adjusted all figures in our manuscript to meet the required technical specifications, including resolution (minimum 300 dpi for grayscale and color), file format (TIFF ), and proper labeling.

We have also utilized the PLOS’s NAAS tool as recommended. We uploaded all figures to NAAS, and based on its assessment, we made necessary corrections to ensure each figure meets the technical specifications. All figures now pass the NAAS quality check.

Attachment Submitted filename: Response_to_Reviewers_auresp_2.docx

# Editor report: Decision Letter 2

**DOI:** 10.1371/journal.pone.0357231.r005

**Authors:** Zeyneb Kurt

13 Aug 2026

Exploration of acetylation-related biomarkers in osteoarthritis through bioinformatics analysis

PONE-D-26-06855R2

Dear Dr. Yao,

We’re pleased to inform you that your manuscript has been judged scientifically suitable for publication and will be formally accepted for publication once it meets all outstanding technical requirements.

Within one week, you’ll receive an e-mail detailing the required amendments. When these have been addressed, you’ll receive a formal acceptance letter and your manuscript will be scheduled for publication.

An invoice will be generated when your article is formally accepted. Please note, if your institution has a publishing partnership with PLOS and your article meets the relevant criteria, all or part of your publication costs will be covered. Please make sure your user information is up-to-date by logging into Editorial Manager at Editorial Manager® and clicking the ‘Update My Information' link at the top of the page. For questions related to billing, please contact billing support.

If your institution or institutions have a press office, please notify them about your upcoming paper to help maximize its impact. If they’ll be preparing press materials, please inform our press team as soon as possible -- no later than 48 hours after receiving the formal acceptance. Your manuscript will remain under strict press embargo until 2 pm Eastern Time on the date of publication. For more information, please contact onepress@plos.org.

Kind regards,

Zeyneb Kurt, PhD

Academic Editor

PLOS One

Additional Editor Comments (optional):

Reviewers' comments:

# Editor report: Acceptance letter

**DOI:** 10.1371/journal.pone.0357231.r006

**Authors:** Zeyneb Kurt

PONE-D-26-06855R2

PLOS One

Dear Dr. Yao,

I'm pleased to inform you that your manuscript has been deemed suitable for publication in PLOS One. Congratulations! Your manuscript is now being handed over to our production team.

At this stage, our production department will prepare your paper for publication. This includes ensuring the following:

* All references, tables, and figures are properly cited

* All relevant supporting information is included in the manuscript submission,

* There are no issues that prevent the paper from being properly typeset

You will receive further instructions from the production team, including instructions on how to review your proof when it is ready. Please keep in mind that we are working through a large volume of accepted articles, so please give us a few days to review your paper and let you know the next and final steps.

Lastly, if your institution or institutions have a press office, please let them know about your upcoming paper now to help maximize its impact. If they'll be preparing press materials, please inform our press team within the next 48 hours. Your manuscript will remain under strict press embargo until 2 pm Eastern Time on the date of publication. For more information, please contact onepress@plos.org.

You will receive an invoice from PLOS for your publication fee after your manuscript has reached the completed accept phase. If you receive an email requesting payment before acceptance or for any other service, this may be a phishing scheme. Learn how to identify phishing emails and protect your accounts at https://explore.plos.org/phishing.

If we can help with anything else, please email us at customercare@plos.org.

Thank you for submitting your work to PLOS One and supporting open access.

Kind regards,

PLOS ONE Editorial Office Staff

on behalf of

Dr. Zeyneb Kurt

Academic Editor

PLOS One
